# Genomic signatures of past and present chromosomal instability in Barrett’s esophagus and early esophageal adenocarcinoma

**DOI:** 10.1038/s41467-023-41805-6

**Published:** 2023-10-04

**Authors:** Chunyang Bao, Richard W. Tourdot, Gregory J. Brunette, Chip Stewart, Lili Sun, Hideo Baba, Masayuki Watanabe, Agoston T. Agoston, Kunal Jajoo, Jon M. Davison, Katie S. Nason, Gad Getz, Kenneth K. Wang, Yu Imamura, Robert Odze, Adam J. Bass, Matthew D. Stachler, Cheng-Zhong Zhang

**Affiliations:** 1 Department of Medical Oncology, Dana-Farber Cancer Institute, 450 Brookline Ave, Boston, MA 02215 USA; 2 Department of Data Science, Dana-Farber Cancer Institute, 450 Brookline Ave, Boston, MA 02215 USA; 3 Department of Pathology, Brigham and Women’s Hospital, 75 Francis St, Boston, MA 02115 USA; 4 Cancer Program, Broad Institute of MIT and Harvard, 415 Main St, Cambridge, MA 02142 USA; 5 Department of Biomedical Informatics, Blavatnik Institute of Harvard Medical School, 10 Shattuck St, Boston, MA 02115 USA; 6 Single-Cell Sequencing Program, Dana-Farber Cancer Institute, 450 Brookline Ave, Boston, MA 02215 USA; 7 Department of Gastroenterological Surgery, Graduate School of Medical Sciences, Kumamoto University, 2 Chome-40-1 Kurokami, Chuo Ward, Kumamoto, Japan; 8 Department of Gastroenterological Surgery, Cancer Institute Hospital of Japanese Foundation of Cancer Research, 3-8-31 Ariake, Koto, Tokyo, Japan; 9 Division of Gastroenterology, Department of Medicine, Brigham and Women’s Hospital, 75 Francis St, Boston, MA 02115 USA; 10 Department of Pathology, University of Pittsburgh School of Medicine, 200 Lothrop Street, Pittsburgh, PA 15213 USA; 11 Department of Surgery, Baystate Medical Center, University of Massachusetts Medical School, 759 Chestnut St, Springfield, MA 01107 USA; 12 Division of Gastroenterology and Hepatology, Mayo Clinic, 200 1st St SW, Rochester, MN 55905 USA; 13 Department of Pathology and Lab Medicine, Tufts University School of Medicine, 145 Harrison Ave, Boston, MA 02111 USA; 14 Department of Pathology, University of California, San Francisco. 513 Parnassus Ave, San Francisco, CA 94143 USA; 15 Present Address: Novartis Institutes for Biomedical Research, Cambridge, MA USA

**Keywords:** Cancer genomics, Tumour heterogeneity, Oesophageal cancer, Genomic instability

## Abstract

The progression of precancerous lesions to malignancy is often accompanied by increasing complexity of chromosomal alterations but how these alterations arise is poorly understood. Here we perform haplotype-specific analysis of chromosomal copy-number evolution in the progression of Barrett’s esophagus (BE) to esophageal adenocarcinoma (EAC) on multiregional whole-genome sequencing data of BE with dysplasia and microscopic EAC foci. We identify distinct patterns of copy-number evolution indicating multigenerational chromosomal instability that is initiated by cell division errors but propagated only after p53 loss. While abnormal mitosis, including whole-genome duplication, underlies chromosomal copy-number changes, segmental alterations display signatures of successive breakage-fusion-bridge cycles and chromothripsis of unstable dicentric chromosomes. Our analysis elucidates how multigenerational chromosomal instability generates copy-number variation in BE cells, precipitates complex alterations including DNA amplifications, and promotes their independent clonal expansion and transformation. In particular, we suggest sloping copy-number variation as a signature of ongoing chromosomal instability that precedes copy-number complexity. These findings suggest copy-number heterogeneity in advanced cancers originates from chromosomal instability in precancerous cells and such instability may be identified from the presence of sloping copy-number variation in bulk sequencing data.

## Abstract (web summary)

Genome complexity is a distinguishing feature of advanced cancers in contrast to precancerous conditions. Here, by analysing chromosomal copy-number evolution in early cancers and precancerous lesions of the oesophagus, the authors reveal signatures of ongoing chromosomal instability and its role in promoting tumour progression.

## Introduction

Large-scale chromosomal rearrangements and copy-number alterations are prevalent in cancer and generally attributed to genomic or chromosomal instability of cancer cells^1–3^. Although much is known about the patterns of genomic rearrangements in fully formed cancers^4,5^ and the biological mechanisms of genome instability^6–8^, little is understood about what mechanisms are active during cancer evolution and how they generate complex cancer genomes.

Genomic analyses of normal tissues have revealed clonally expanded point mutations but not large structural chromosomal aberrations^9,10^. Early-stage precancerous lesions also show significantly less genome complexity than late-stage dysplasia^11–15^ or cancer^4,16,17^. These observations have led to the prevailing view that most chromosomal rearrangements arise late during cancer progression in an episodic manner^18,19^, in contrast to the gradual accumulation of short sequence variants (single-nucleotide substitutions or short insertions/deletions)^20,21^. However, the apparently simple genomes of precancerous lesions at the clonal level does not exclude genome instability or complexity at the cellular level. Cells with unstable genomes will generate copy-number variation in the progeny^22,23^, but such variation is invisible at the population level due to counterbalancing of random copy-number gains and losses in single cells in the absence of selection (i.e., neutral evolution). Genetic variation is also suppressed by positive selection (e.g., for oncogene amplifications) or negative selection (against large DNA deletions or aneuploidy in general^24^). Based on these considerations, the footprint of genome instability in somatic cells should be most visible in small precancerous lesions with in situ clonal expansion of copy-number variation. This idea further suggests that the origin of genome complexity in advanced cancers may be revealed by analyzing genetic variation and evolution in precancer conditions.

Barrett’s esophagus (BE)^25–27^ is the only known precursor of esophgeal adenocarcinoma (EAC) and estimated to be present in 60-90% of newly diagnosed EAC cases^28^. In contrast to fully formed EACs with complex chromosomal changes^29^, BE tissue can contain lesions of different histopathological states with varying genomic complexity^30,31^. In this study, by analyzing copy-number alterations in concurrent BE (both non-dysplastic and dysplastic) and early EAC (either intramucosal or T1) lesions, we reveal copy-number heterogeneity in BE cells before transformation, relate copy-number evolution patterns in BE cells to those derived from experimental models of chromosomal instability^32–38^, and provide mechanistic insight into the evolution of EAC genome complexity. We find that both copy-number heterogeneity and complexity can predate the appearance of cancers or dysplastic lesions and are present in both single BE cells and BE subclones with intact p53. Loss of p53 enables episodic but multigenerational genome evolution initiated by catastrophic events such as whole-genome duplication^32,33^, chromothripsis^34–36^, and dicentric chromosome formation^37,38^, which can precipitate copy-number heterogeneity and complex copy-number gains in BE cells. We further present examples of copy-number patterns that reflect ongoing chromosomal instability, including progressive DNA deletions in BE cells that result in sloping copy-number variation and distinct oncogenic amplifications in independently transformed cancers within a single BE field. Together, these findings elucidate how genome instability drives copy-number evolution to promote tumor progression.

## Results

### Copy-number heterogeneity suggests early onset of chromosomal instability in precancer BE cells

**Fig. 1:**
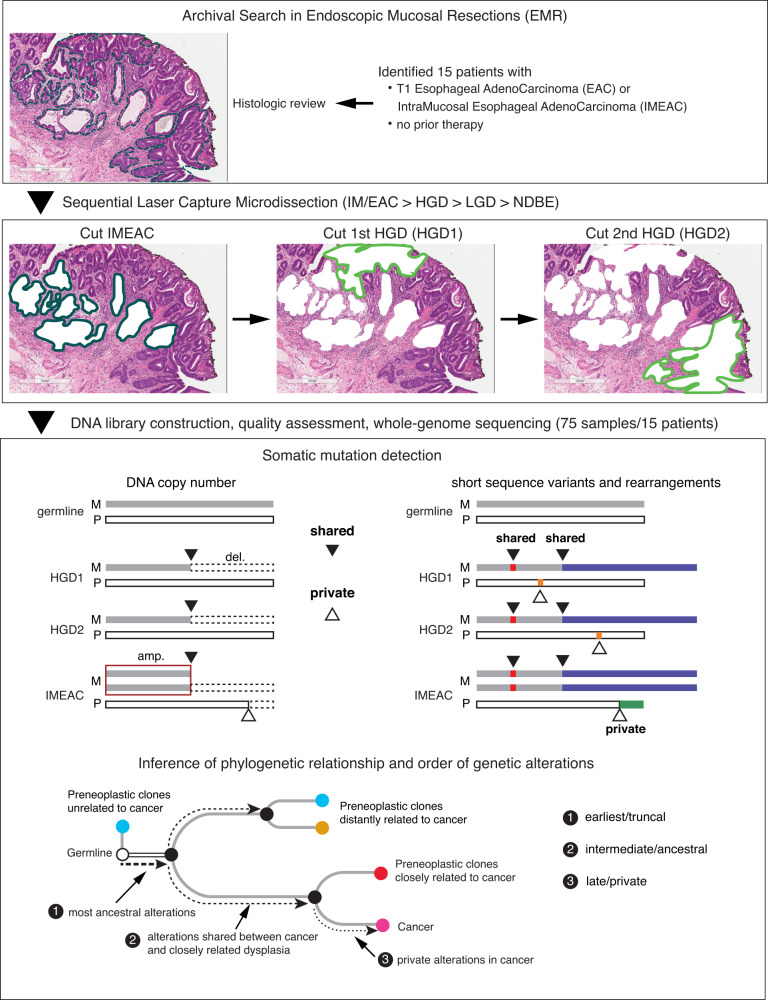
Overview of experimental design and bioinformatic analysis. Top: 15 patients whose Barrett’s esophagus tissue samples presented early invasive esophageal adenocarcinomas (EAC) were selected. Middle: After histological review, 75 samples of early cancer (EAC) and precancerous lesions, including non-dysplastic Barrett’s esophagus (NDBE), low-grade dysplasia (LGD), and high-grade dysplasia (HGD), were collected via laser capture microdissection and subjected to whole-genome sequencing. See Supplementary Data 1 for a complete list of samples from each individual. Bottom: We perform joint variant detection on samples from each patient and then determine their phylogeny based on genetic alterations shared by two or more samples (filled triangles). Based on the phylogeny, we then infer the timing and evolution of copy-number alterations (both shared and private) on each parental chromosome (maternal or parental), including distinct copy-number changes on a single parental chromosome in related BE/EAC genomes generated by branching evolution.

Endoscopic mucosal resection (EMR) is routinely performed in patients with dysplastic BE. In reviewing more than 500 formalin-fixed, paraffin-embedded (FFPE) EMR samples, we identified 14 cases showing unexpected microscopic foci of invasive cancers and one case (patient 1) with an early cancer removed via esophagectomy. All cancers were either intramucosal or T1 and all samples were collected before treatment. Following independent pathologic re-review by two or more pathologists to confirm the diagnoses (“Methods” section), we delineated and performed laser capture microdissection (LCM) to isolate regions corresponding to distinct histopathological states^27^ (Fig. 1), including non-intestinalized columnar metaplasia (COLME), non-dysplastic BE (NDBE), BE indefinite for dysplasia (IND), BE with low-grade dysplasia (LGD) or high-grade dysplasia (HGD), and intramucosal (IMEAC) or early EAC (Supplementary Fig. 1**)**. We further isolated normal tissue from benign FFPE regions that was used as germline reference.

Due to the limited quantity of FFPE DNA from small tissue sections and their lesser quality compared to DNA from fresh or frozen cells, we first performed low-pass whole-genome sequencing (WGS) at ~0.1x mean depth to select libraries with sufficient complexity and then performed deeper sequencing ~20x. The final cohort consisted of 75 BE/EAC (21 COLME/NDBE/IND, 7 LGD, 23 HGD, and 24 IM/EAC) and 15 reference samples from 15 patients (Supplementary Data 1). FFPE libraries harbor various technical artifacts that limit the accuracy of variant calls generated by standard tools (Supplementary Information. For single-nucleotide variants (both somatic and germline), short insertions/deletions, and rearrangements, we performed joint variant detection on all samples from each patient to improve variant detection accuracy (Fig. 1 and “Methods” section). Although the joint analysis is sufficient to detect mutations shared by multiple samples, false negative mutation detection in individual samples due to sequencing dropout still confounds phylogenetic inference. To bypass this challenge, we focused on somatic copy-number alterations (SCNA) for which better accuracy could be achieved and used point mutations to independently validate the phylogeny inference (Supplementary Information).

We determined chromosome-specific DNA copy number and copy-number changepoints based on haplotype-specific sequence coverage (“Methods” section and Supplementary Information). Parental haplotypes were first inferred by statistical phasing using a reference haplotype panel^39^ and then refined based on allelic imbalance across all samples from each patient. We used haplotype-specific sequence coverage to first validate the estimated ploidies (i.e., average chromosomal copy number) and clonal fractions of aneuploid BE/EAC clones and then calculate the integer DNA copy number of both parental chromosomes. The determination of long-range parental haplotype both enabled phasing of SCNAs to each parental chromosome and ensured the accuracy of SCNA detection. We further performed segmentation of haplotype-specific DNA copy number and used copy-number changepoints to refine the list of rearrangements. For data presentation clarity, the copy-number plots in the main and supplementary figures only show data of the altered homolog, except where stated. The haplotype-specific sequence coverage and copy number of both homologs are available in the Online Data Repository (see Data Availability).

**Fig. 2:**
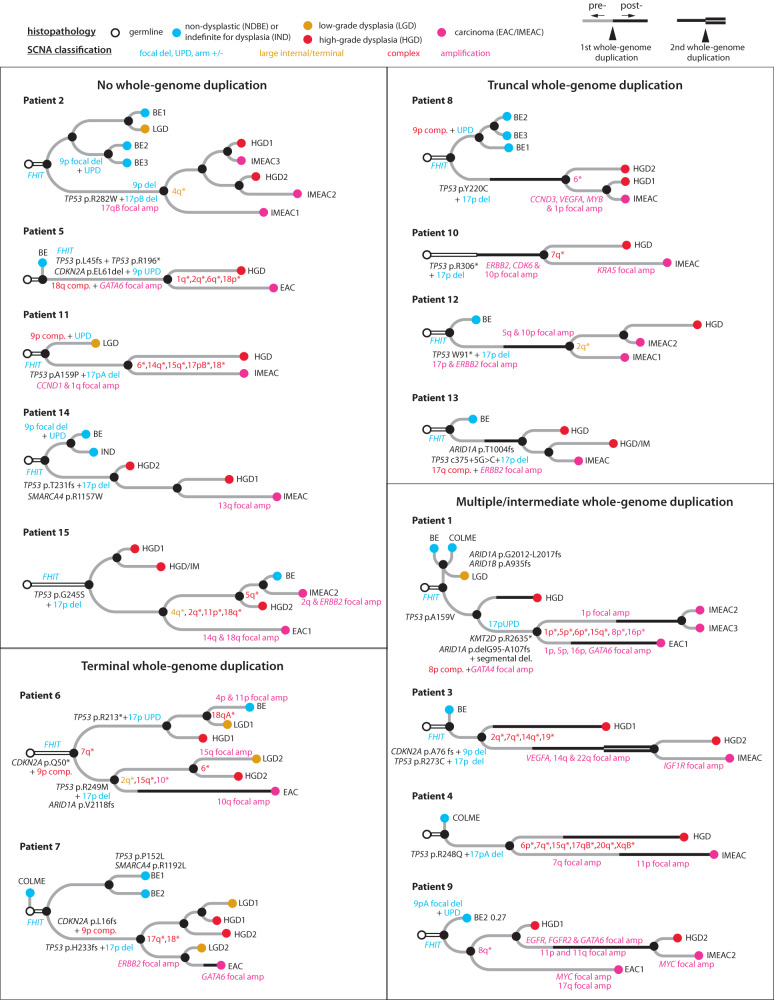
Phylogeny of early EAC and precursor BE lesions within a single BE field from each patient determined by haplotype-specific copy-number alterations. For a comparison against single-nucleotide-mutation derived phylogeny, see Supplementary Fig. 2 and Supplementary Information. Phylogenetic trees are grouped based on the timing of whole-genome duplication (WGD, thick solid line). Samples are colored based on their histopathology grading: blue for non-dysplastic BE (BE) or BE indefinite for dysplasia (IND), orange for low-grade dysplasia (LGD), red for high-grade dysplasia (HGD), and magenta for carcinoma (EAC or IMEAC). Samples with both HGD and IMEAC features are annotated as HGD/IM. The branch length (horizontal distance between nodes) approximately reflects the number of altered chromosomes. For a complete list of alterations along each evolutionary branch, see Supplementary Data 2. Annotated alterations include: (1) recurrent alterations or those affecting known EAC drivers; (2) focally amplified regions or oncogenes (magenta); (3) chromosomes or chromosome arms (with asterisks) with divergent copy-number alterations in more than one progeny clones. Note that patient 13 contained a splice-site mutation (c.375+5 G > C) in *TP53* that was assessed to produce truncated p53^64^ and also reported to be a recurrent mode of p53 inactivation in cancers^65^. The colors of annotated chromosomes reflect the complexity of copy-number alterations: simple deletion/duplication, uniparental disomy, arm-level gain/loss (blue), large segmental (terminal or internal) copy-number changes or their combinations (orange), complex copy-number alterations (red), focal amplifications (magenta). For classification of copy-number alterations, see Supplementary Fig. 4.

We determined the phylogenetic tree of samples from each patient (Fig. 2) based on haplotype-specific copy-number alterations (“Methods” section). SCNAs were first identified independently in each sample and then assigned to phylogenetic branches based on their presence or absence in all samples. The branch length (horizontal distance between nodes) approximately reflects the SCNA burden estimated using the number of altered chromosomes. SCNAs on each branch (labeled in Supplementary Fig. 2) are summarized in Supplementary Data 2; SCNAs that affect esophageal cancer genes or identified more than once in the current cohort are annotated in Fig. 2. In all but two patients (13 and 14), we identified SCNAs in related BE/EAC genomes affecting a single parental chromosome but having distinct changepoints that suggest branching evolution of ancestral chromosomes; these chromosomes are labeled with asterisks near the inferred common ancestor. Whole-genome duplication (WGD) was inferred based on the number of homologous chromosomes with more than one copy^40^ and assigned to evolutionary branches based on the WGD status of individual samples. For SCNAs on branches with WGD, their timing relative to WGD was inferred based on the integer copy-number states. Finally, we confirmed the consistency and genetic similarities between SCNA-derived and somatic SNV-derived phylogenetic trees (Supplementary Fig. 2). The few instances of discrepancy are discussed in Supplementary Information.

The phylogenetic trees of EAC and precursor BE lesions show several recurrent patterns. First, bi-allelic *TP53* inactivation is a truncal event of the evolutionary branches of cancer or high-grade BE lesions (14/15 patients). By contrast, focal deletion near *FHIT* (a common fragile site) is often ancestral to all BE and EAC lesions; bi-allelic inactivation of *CDKN2A* (a frequently inactivated tumor suppressor) can be truncal to either cancer/HGD lesions (patient 3, 5, 6, and 7) or NDBE/LGD lesions (patients 2, 8, 9, 11, and 14). Second, evolutionary branches with the highest SCNA burdens are frequently associated with WGD, which is itself also a frequent event (10/15 patients). Third, high-grade dysplastic BE lesions and cancer lesions from the same patient often harbor distinct SCNA breakpoints on single parental chromosomes (13/15 patients) or distinct regions of focal amplification (10/15 patients), indicating copy-number heterogeneity prior to the emergence of aneuploid BE/EAC clones. Finally, we identified more than one early cancer lesion in five patients (patients 1, 2, 9, 12, and 15): The distinct cancer foci from each patient often displayed significant genomic divergence but were individually accompanied by precancerous lesions in close proximity (patients 1, 9,12, and 15) and/or showing more genomic similarity (patients 2, 9, 12, and 15). The last observation strongly suggests that the cancer foci had evolved independently from distinct BE cells within the same BE field, i.e., independent malignant transformation.

**Fig. 3:**
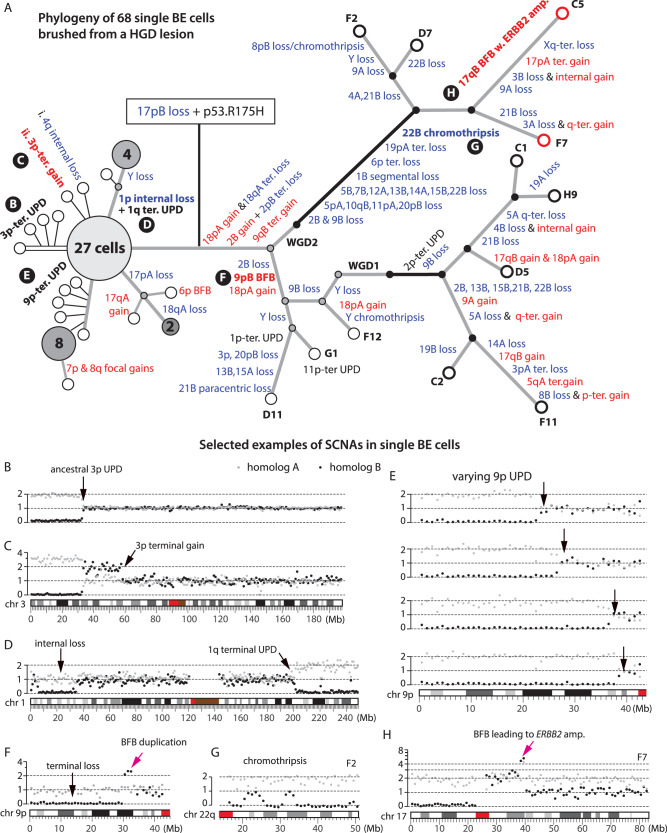
Copy-number evolution in 56 near diploid and 12 aneuploid BE cells from a high-grade dysplastic Barrett’s esophagus determined by single-cell sequencing. **A** Phylogenetic tree with annotated haplotype-specific copy number alterations (blue for losses, red for gains). Each open circle represents a single cell; large filled circles represent subclones (with annotated cell counts) with identical copy number; small filled circles represent inferred intermediate states (gray for pre-WGD, black for post-WGD). Aneuploid cells are separated into two branches each inferred to have undergone an independent whole-genome duplication (WGD) event (black solid line). **B**–**H** Examples of copy-number alterations before (**B**–**E**) and after (**F**–**H**) p53 inactivation. Gray and black dots represent haplotype-specific DNA copy number of parental chromosomes. **B** Ancestral 3p uniparental disomy (UPD) shared by all but four cells. **C** Sporadic 3p terminal gain after 3p UPD in one cell. **D** Large paracentric deletion on 1p and uniparental disomy (UPD) at the 1q-terminus shared by five cells. **E** Progressive 9p UPD in a subclone of 14 cells. Only four cells are shown, see ”Online Data” for the others. **F** Terminal duplication adjacent to terminal deletion on 9p shared by cell G1 and D11 that is consistent with two rounds of breakage-fusion-bridge cycles. **G** Chromothripsis of chr22q shared by cell C5, F2, and F7. **H** Focal amplification spanning the *ERBB2* gene on chr17q (~40 Mb) in cell C5 and F7 (red circles) that displays the signature copy-number pattern of breakage-fusion-bridge cycles. For a detailed list of alterations in each cell, see Supplementary Data 3.

The observation of significant SCNA diversity in BE and EAC subclones suggests highly dynamic copy-number evolution in precancerous BE cells and predicts copy-number diversity at the single-cell level. We directly tested this prediction by performing whole-genome sequencing on 68 single cells isolated from a patient with known HGD by endoscopic cytology brushing immediately before radiofrequency ablation. We performed haplotype-specific copy-number analysis and phylogenetic inference using the same strategy as for bulk samples (“Methods” section). We identified 12 cells with aneuploid genomes and 56 cells with near diploid genomes. Their phylogeny and selected examples of SCNAs in single BE cells or subclones are shown in Fig. 3**;** SCNAs in each cell are listed in Supplementary Data 3 and DNA copy-number plots of all cells are available in the Online Data Repository. All the aneuploid cells share biallelic *TP53* inactivation (through a pathogenic R175H mutation and loss-of-heterozygosity generated by 17p loss) but show significant heterogeneity of chromosomal copy-number changes. The onset of genomic heterogeneity in precancer BE cells following biallelic *TP53* inactivation recapitulates the pattern seen in bulk samples and provides direct evidence of dynamic precancer genome evolution driven by chromosomal instability. We next discuss specific patterns of copy-number evolution and their mechanistic implications.

### *TP53* inactivation and the onset of genome instability initiates BE genome evolution

**Fig. 4:**
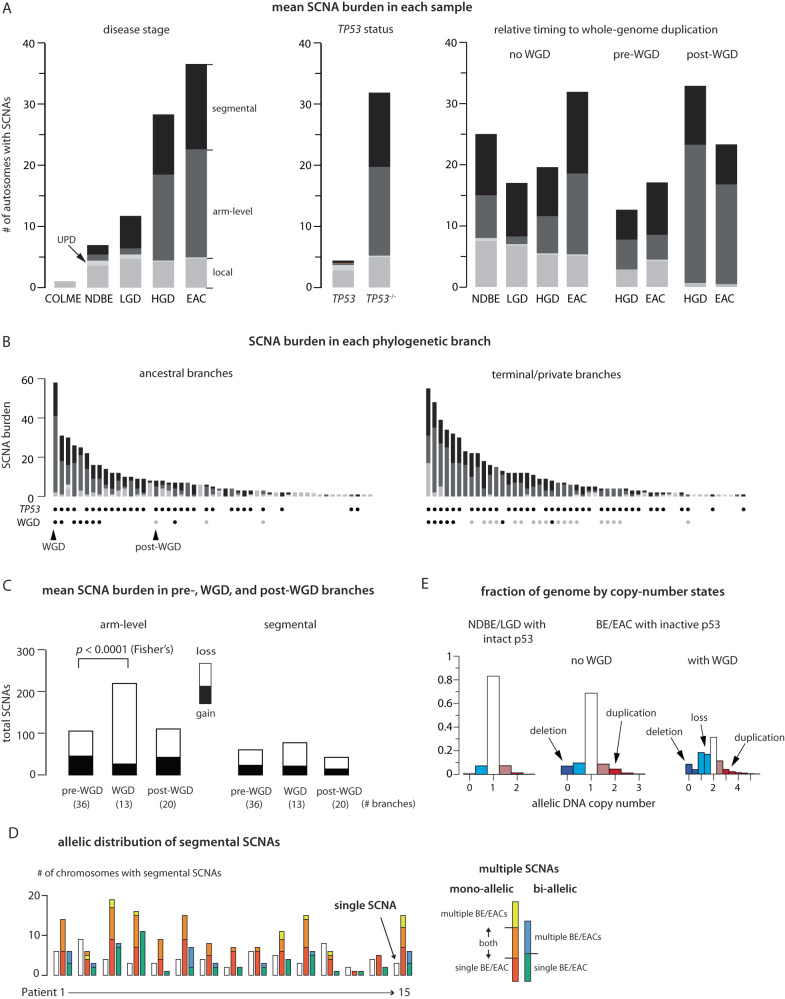
Landscape of somatic copy-number alterations (SCNA) in BE and EACs. **A** Mean SCNA burden in samples grouped by disease stage (left), *TP53* mutation status (*middle*), and timing relative to whole-genome duplication (right). The SCNA burden is measured by the total number of altered autosomes (both parental homologs, maximum 44) and subdivided into local deletions or duplications (gray), uniparental disomies (light gray), arm-level SCNAs (dark gray), and segmental SCNAs (black). See Supplementary Fig. 3A–D for the SCNA burden in each sample and of subcategories of segmental SCNAs. In the middle panel, the ‘intact’ *TP53* group (“*TP53*”) only includes NDBE/LGD samples without detectable *TP53* alterations, but not HGD/EAC samples. See Supplementary Fig. 3C for the SCNA burden in HGD/EAC samples without p53 inactivation. **B** SCNA burden along ancestral (having more than one progeny clone) and terminal (only one progeny clone) phylogenetic branches. The bottom shows the *TP53* mutation status and the relative timing to WGD of each branch. **C** Total counts of arm-level (left) and segmental (right) SCNAs (filled bars for gains, open bars for losses) in evolutionary branches preceding, concurrent with, or after WGD. Segmental SCNAs only include large internal/terminal SCNAs but not complex SCNAs that can generate both DNA gain and loss. The significantly higher burden of arm-level SCNAs in WGD-concurrent branches than pre-WGD branches (Mann-Whitney *p* = 3 × 10^-6^; 95% Confidence Interval: 7-16; Effect Size: 0.68) is dominated by chromosome losses (Two-sided Fisher’s test *p* = 10^−9^; 95% Confidence Interval: 0.10–0.33; Effect Size: 0.18,), consistent with chromosome losses after tetraploidization. WGD is also associated with a modest but significant increase of segmental SCNA burden (WGD-concurrent vs pre-WGD: Mann–Whitney *p* = 0.0071; 95% confidence interval: 1–5; effect size: 0.43) and of arm-level SCNAs (post-WGD vs pre-WGD: Mann-Whitney *p* = 0.0032; 95% confidence interval: 1–4; effect size: 0.40). **D** Allelic distribution of segmental SCNAs identified in all samples from each patient. Shown are the number of chromosomes (Chrs.1-22 and X) with single SCNAs (open bars), multiple SCNAs affecting a single parental homolog (‘mono-allelic’), or multiple SCNAs affecting both homologs (‘bi-allelic’). Mono-allelic and bi-allelic SCNAs with multiple breakpoints are further divided into subcategories based on whether SCNA breakpoints are found in a single BE/EAC genome, or in multiple related BE/EAC genomes. See Supplementary Fig. 3E. **E** Fraction of the germline genome at different copy-number states (from 100kb-level allelic copy number). Deletion (dark blue), subclonal deletion/loss (light blue), subclonal gain (light red), or duplication (dark red).

We observed increasing SCNA burden with disease progression (Fig. 4A, left and Supplementary Fig. 3A, B), but this correlation is mostly attributed to *TP53* mutation status. Samples with *TP53* inactivation show significantly higher SCNA burdens than samples without *TP53* inactivation (Fig. 4A, middle and Supplementary Fig. 3C). In particular, two NDBE samples from patients 6 and 15 and four LGD samples from patients 6 and 7 with bi-allelic *TP53* inactivation show similar SCNA burdens and complexity as HGD and EAC samples; by contrast, NDBE and LGD samples without *TP53* inactivation show fewer SCNAs (Supplementary Fig. 3A). These data and the contrasting SCNA burdens in single BE cells with and without intact p53 (Fig. 3A) both reinforce the association between p53 loss and SCNA evolution^11,31^.

Prior analyses of ageing esophageal tissues^9,10^ by bulk sequencing revealed uniparental disomy (UPD), or copy-neutral loss-of-heterozygosity, as the only large segmental SCNA. Consistent with this observation, we observed frequent UPDs in both single BE cells (Supplementary Data 3) and clones (Supplementary Data 4) prior to p53 loss, but only sporadic segmental gains or losses in single BE cells (Fig. 3C, D) and almost none in BE clones. Remarkably, we identified UPDs on the 9p terminus with varying boundaries in a subclone of 14 near-diploid BE cells (Fig. 3E and “Online Data”). As this variation does not alter total DNA copy number, it can only be revealed by haplotype-resolved copy-number analysis. The varying boundaries of terminal UPD in different cells (arrows in Fig. 3E) bear an intriguing similarity to our prior observation of varying terminal deletions attributed to ongoing breakage-fusion-bridge cycles^38^ (see Supplementary Fig. 7 that will be discussed later). The similarity between varying terminal UPDs and varying terminal deletions suggests a plausible common origin from chromosomes with unprotected broken ends^23^, with deletions resulting from translocations involving other broken ends and UPDs resulting from homology-dependent invasion of broken ends into the intact homolog followed by a half crossover resolution^41^ (Supplementary Fig. 4, top).

In contrast to the simple SCNA landscape in BE cells with intact p53 is the prevalence of arm-level and complex SCNAs in BE cells and clones after p53 loss. Loss of p53 does not directly cause aneuploidy or chromosomal instability in human cells^42^, but abolishes p53-dependent arrest after DNA damage^43^ or prolonged mitosis^44^. The burst of SCNA complexity after p53 loss is therefore more likely to reflect an increased frequency of SCNA clonal expansion than an increased rate of SCNA acquisition. Moreover, the observation of sporadic large SCNAs, especially UPDs, in single BE cells with intact p53 indicates that BE cells do acquire DNA breaks, but these breaks do not lead to complex copy-number alterations as seen in BE cells or clones with inactive p53. We next focus on BE cells or clones with inactive p53 and provide evidence supporting that the accumulation of SCNA complexity reflects multigenerational chromosomal instability that is precipitated by sporadic cell division errors but only propagated after p53 inactivation.

### Whole-genome duplication triggers rapid accumulation of arm-level copy-number changes

The most dramatic change in BE cells is whole-genome duplication (WGD). WGD is inferred to be a frequent event in many epithelial cancers^45,46^ and thought to define a particular EAC evolution trajectory^31^. We inferred 15 WGD events in bulk BE/EAC lesions from 10/15 patients, including independent WGD occurrences in distinct HGD/EACs from patients 1,3, and 4 (Fig. 2). We further inferred two independent WGDs in single BE cells without presence of cancer (Fig. 3A). These observations suggest that WGD may occur frequently during BE progression before the appearance of cancer.

Despite the prevalence of WGD in human cancers^45,46^ and its tumor-promoting capacity^47,48^, how WGD impacts tumorigenesis remains incompletely understood. One proposal is that tetraploidization (the event that causes WGD) can precipitate additional genome instability including multipolar cell division or chromosome missegregation^6,32,33^ that leads to aneuploidy. Consistent with this model, we inferred that more SCNAs in BE/EAC genomes were acquired after WGD than before WGD (Fig. 4A, right), and evolution branches with WGD acquisition had significantly higher SCNA burdens (30 events/branch) than non-WGD branches (pre-WGD: 7.5/branch; post-WGD: 8.8/branch) (Fig. 4B and Supplementary Data 2). Moreover, a majority of post-WGD SCNAs are arm-level changes (302 out of 428 events) and dominated by losses (256/302, Fig. 4C), a pattern also seen in single aneuploid BE cells (Fig. 3A).

The preponderance of chromosome losses after WGD has two implications. First, this pattern cannot be solely explained by increased rates of random chromosome missegregation^32^ that generates reciprocal gains and losses between daughter cells. This pattern could reflect a lower fitness of cells with larger chromosome number due to more frequent mitotic delays and defects^46^. It could also arise from multipolar cell divisions that generate three or more progeny cells with predominantly chromosome losses^33^ (Supplementary Fig. 5A). Future work is needed to test these hypotheses. Second, extensive chromosome losses after WGD may significantly reduce the number of duplicated chromosomes and cause underestimation of WGD incidence in cancer development, especially in cancers with highly aneuploid genomes. Together, our analysis of arm-level SCNAs in BE cells both confirms WGD as a precursor to aneuploidy^49–51^ and highlights the diversity of copy-number outcomes^5^ generated by post-WGD events including multipolar cell division^33^.

### Segmental copy-number alterations display signatures of dicentric chromosome evolution

In contrast to the prevalence of post-WGD arm-level SCNAs, we inferred a similar number of segmental SCNAs in BE/EAC genomes to have occurred prior to (135) and after WGD (126) in samples with WGD acquisition. The fractions of segmental DNA loss and DNA gain are also comparable among pre-, post-, and WGD branches (Fig. 4C, right), although branches with WGD acquisition have a higher average SCNA burden (5.9 events) than pre- (1.6) or post-WGD (2.1) branches. These observations indicate that segmental SCNA acquisition is promoted by WGD but also occurs independent of WGD.

Segmental SCNAs in BE genomes further display two features of non-randomness. First, SCNA breakpoints are often concentrated on a few chromosomes with complex deletions (chromothripsis) or duplications. Second, distinct SCNAs in related BE/EAC genomes more frequently originate from a single parental chromosome (‘mono-allelic’) than affect both parental chromosomes (‘bi-allelic’) (Fig. 4D and Supplementary Fig. 3E). Both features are more consistent with one-off or successive SCNA acquisition on individual unstable chromosomes than independent SCNA acquisition on both parental chromosomes. The connection between segmental SCNA acquisition and chromosomal instability is further supported by the observation of larger fractions of deletions (allelic copy number = 0) or duplications (allelic copy number ≥2 in non-WGD samples and ≥ 3 in WGD samples) in samples with inactive p53 than in samples with intact p53 (Fig. 4E). Finally, we recognized that many segmental SCNA patterns in BE/EAC genomes are consistent with the outcomes of chromosomal instability from abnormal nuclear structures including micronuclei^34^ (Supplementary Fig. 5B) and chromosome bridges (Supplementary Fig. 5C)^38^. We sought to use the genomic signatures of in vitro chromosomal instability to deconvolute segmental copy-number complexity in BE/EAC genomes.

**Fig. 5:**
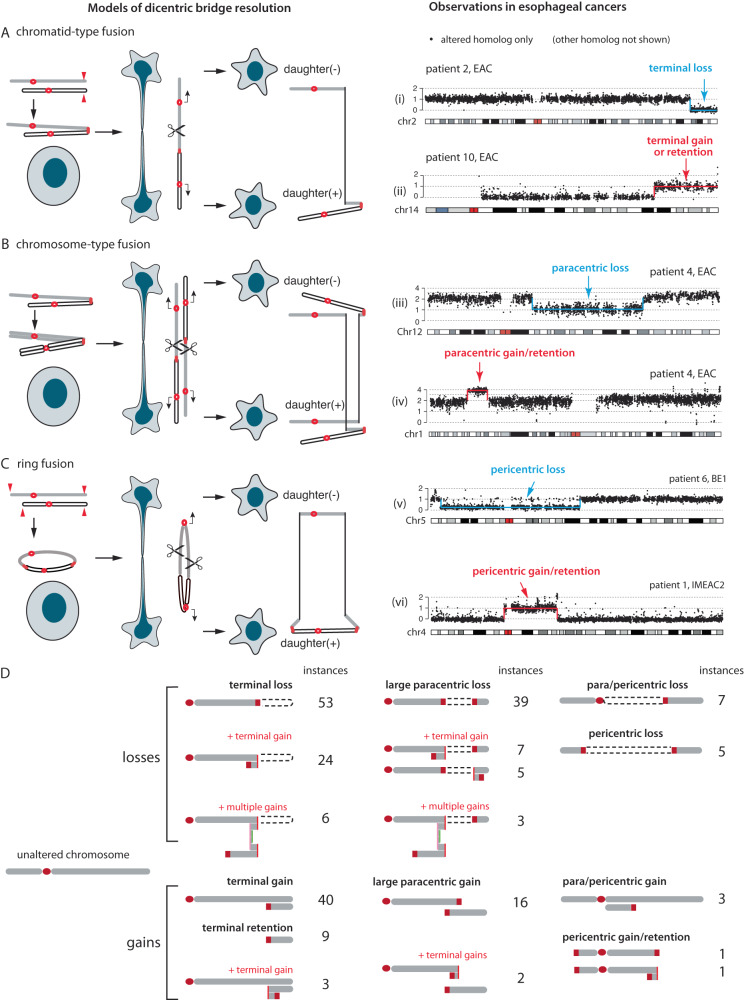
Segmental copy-number alterations in BE/EAC genomes that match the outcomes of dicentric chromosome bridge resolution. **A**–**C** (Left) Different types of dicentric chromosome breakage and their copy-number outcomes: (**A**) terminal; (**B**) paracentric; or (**C**) pericentric segmental copy number changes. The open and filled chromatids may be sister chromatids or different chromosomes. Both **A** and **B** were demonstrated in vitro in ref. ^38^. The model that pericentric copy-number changes may arise from broken dicentric ring chromosomes (**C**) or multicentric chromosomes (when the p- and q-termini of a chromosome are fused to two other chromosomes) has not been demonstrated in vitro but is plausible as telomere crisis can lead to multiple shortened telomeres that generate dicentric rings. (Right) Examples of SCNAs in BE/EAC genomes that recapitulate the predicted SCNA outcomes of bridge resolution. The allelic copy-number plots (25 kb bins) show the DNA copy number of the altered chromosome; the intact homolog is not shown. Examples of gain and loss in each group are unrelated. See “Online Data” for the copy-number plots of both homologs in each sample. **D** Summary of terminal/internal SCNAs in BE/EAC genomes, including copy-number patterns consistent with different combinations of successive BFB cycles with SCNA outcomes shown in **A**–**C**. Numbers denote instances of each pattern. See Supplementary Data 5 for the complete list.

The most frequent SCNAs in BE/EAC genomes are gain or loss of large terminal (i.e., spanning a telomere) or internal (with two non-telomeric breakpoints) segments; these alterations are consistent with the outcomes of dicentric chromosome breakage (Fig. 5). Dicentric chromosomes can result from either end-to-end chromosome fusion or incomplete decatenation of sister chromatids^38^ and lead to a ‘bridge’ between daughter nuclei when the two centromeres segregate to different daughter nuclei. Although dicentric chromosomes can be generated by a variety of mechanisms, the genomic consequences are primarily determined by the formation and breakage of chromosome bridges^37,38^. Breakage of a single dicentric chromosome (‘chromatid-type’ bridges) will generate reciprocal gain and loss of a telomeric segment (‘terminal’ SCNAs) (Fig. 5A). If both sister dicentric chromatids are part of the bridge (‘chromosome-type’ bridges), their breakage can give rise to large segmental gain or loss within a chromosome arm, hereafter referred to as ‘paracentric’ SCNAs (Fig. 5B). Both of these outcomes were directly demonstrated in single-cell experiments^38^ but originally described by McClintock (summarized in ref. ^52^) We further observed large SCNAs spanning centromeres (‘pericentric’ SCNAs) that can result from broken ring chromosomes (Fig. 5C, first described by McClintock in ref. ^53^) or multicentric chromosomes. The instances of terminal and large internal SCNAs in our BE/EAC cohort are summarized in Fig. 5D and listed in Supplementary Data 5. In total, these events account for ~50% of segmental SCNAs.

Although chromosome bridge resolution provides a simple mechanism for single-copy gain or loss of large segments, similar copy-number outcomes may be generated by other processes. For example, terminal deletion or duplication could result from simple chromosomal translocations followed by whole-chromosome losses or gains (Supplementary Fig. 6A). This model, however, produces an equal number of terminal gains (including retentions) and losses, and cannot explain the disparity between terminal gains and losses seen in most samples (Supplementary Fig. 6B). Moreover, as broken bridge chromosomes can form new dicentrics and undergo breakage-fusion-bridge (BFB) cycles that generate a variety of compound copy-number outcomes, the identification of these compound copy-number patterns in BE/EAC genomes provides stronger evidence of chromosome bridges being involved in BE copy-number evolution.

**Fig. 6:**
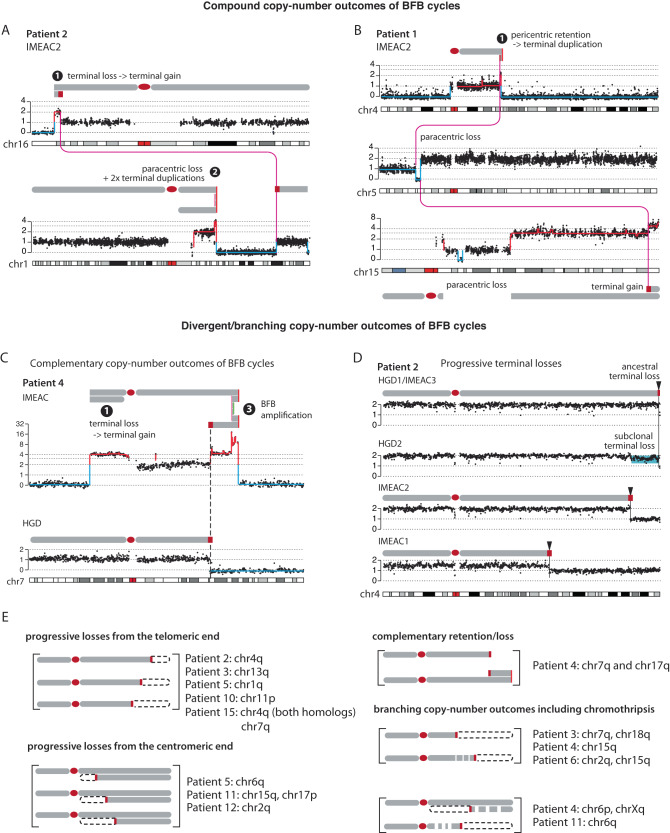
Segmental copy-number patterns in BE/EAC genomes consistent with the outcomes of multigenerational breakage-fusion-bridge cycles. Arabic numbers represent outcomes of different sequences of BFB evolution as labeled in Supplementary Fig. 5D. Schematic diagrams of altered chromosomes are drawn according to the segmental DNA copy number. **A** (Top) Terminal deletion -> terminal duplication; (bottom) paracentric deletion -> two duplications near the centromeric break end. **B** (Top) Pericentric retention -> duplication at the q-terminus; (middle) paracentric deletion -> whole-chromosome duplication of the centromeric segment; (bottom) whole-chromosome duplication -> pericentric loss + terminal gain -> whole-genome duplication. Magenta lines represent joining between broken fragments. See Supplementary Fig. 6 for more examples. **C** Complementary copy-number gain and loss at a single breakpoint (dashed line) in HGD and IMEAC reflect two broken pieces of a single dicentric chromosome. The focally amplified region on the telomeric end in IMEAC is consistent with BFB amplifications either preceding or after the breakage event. **D** A series of terminal deletions on the same parental chromosome present in five lesions from patient 2. The proximal boundaries of the subclonal DNA loss near the 4q-terminus in HGD2 and clonal DNA loss in IMEAC2 suggest that IMEAC2 may have evolved from a subclone in HGD2. See Supplementary Fig. 7 for examples of the same pattern revealed in experimental BFB evolution. **E** Summary of SCNAs in related BE/EAC genomes reflecting divergent/branching BFB outcomes. See Supplementary Data 6 and “Online Data” for the copy-number plots of each instance.

The most common outcome of successive BFB cycles is the presence of DNA duplications near the boundaries of large segmental deletions (Fig. 6A, B) or large segmental gains. Instances of these patterns in BE/EAC genomes are listed in Supplementary Data 5 and also summarized in Fig. 5D. The identification of interchromosomal rearrangements between both simple and compound SCNA breakpoints (Fig. 6A, B and Supplementary Fig. 6C, D) also suggests that these broken ends were generated simultaneously, most likely from the resolution of multichromosomal bridges as seen in experimental models of telomere crisis^37^ or chromosome bridge resolution^38^.

Successive DNA duplications at the broken ends of chromosomes can generate focal amplifications (Fig. 6C, top). Remarkably, the amplification on 7q in IMEAC (spanning the *MET* oncogene) shares a common SCNA boundary with the terminal deletion in HGD. (The same pattern of reciprocal DNA retention and loss is also seen in 17q of these two clones.) This pattern of reciprocal DNA retention and deletion directly recapitulates the outcome of broken bridge chromosomes between daughter nuclei (Fig. 5A) that is only visible by multiregional sequencing. Based on this observation, we inferred the ancestors of the HGD and the IMEAC clones may be traced to a pair of daughters each having inherited a broken piece of a dicentric chr7.

Besides DNA duplications at broken termini, BFB cycles can also generate progressive DNA losses from either sequential breakage or deficient replication of bridge chromatin^38^. As each new deletion erases the boundary of preceding deletions, progressive DNA losses can only be revealed in different progeny clones (Supplementary Fig. 7) but not in a single clone. We observed 11 instances of terminal or paracentric SCNAs with distinct breakpoints in different BE/EAC lesions from the same patient that are consistent with progressive DNA losses (Supplementary Data 6). One example of varying 4q-terminal losses (boundaries marked by black arrows) in five lesions from patient 2 is shown in Fig. 6D.

In summary, we identified frequent duplications or deletions of large terminal, paracentric, and pericentric segments in BE genomes and attributed them to the formation and breakage of dicentric chromosomes (Fig. 5). This mechanistic association is further supported by the observation of (1) additional duplications or progressive DNA losses at SCNA boundaries (Fig. 6) reflecting successive BFB cycles (Supplementary Fig. 7); and (2) interchromosomal translocations between SCNA boundaries indicating simultaneous generation of broken chromosome ends. In particular, the observation of reciprocal DNA loss and gain in distinct BE/EAC clones from the same patient that directly recapitulate the outcome of dicentric bridge resolution between daughter cells (Fig. 6C) provides the most compelling evidence of BFB cycles during BE evolution.

### Contemporaneous chromothripsis and BFB cycles generate EAC copy-number complexity

Besides simple DNA loss and gain, dicentric chromosomes can also undergo DNA fragmentation^37,38^ either from chromosome bridge resolution or in micronuclei from chromosome missegregation. These processes generate chromothripsis with different footprints. For chromothripsis from bridge resolution, fragmentation of the bridge chromatin creates oscillating copy number in a fraction of the chromosome arm that was in the bridge, and the region with oscillating copy number is usually adjacent to the boundaries of large terminal or internal SCNAs corresponding to termini of broken bridge chromosomes (Supplementary Fig. 8A). We inferred 34 instances of chromothripsis were consistent with this pattern (Supplementary Data 7: Table 1, ‘direct’ or ‘likely direct’ in Column N) and show representative examples in Supplementary Fig. 8B. For chromothripsis resulting from fragmentation of broken bridge chromosomes in downstream micronuclei, the oscillating copy-number pattern should span a centromeric or telomeric segment (Supplementary Fig. 8C, D), or an entire chromosome arm (Supplementary Fig. 8E–G). We inferred 26 instances of chromothripsis were consistent with this evolution sequence (Supplementary Data 7: Table 1, ‘downstream’ or ‘possibly downstream’ in Column N). We note that chromosome bridges may contain entire chromosomes and generate chromothripsis that is indistinguishable from the outcome of downstream micronucleation of the broken chromosome^38^; therefore, direct or downstream chromothripsis from bridge resolution may not be strictly distinguishable. We also identified 7 instances of regional chromothripsis without a definitive relationship to large terminal/internal SCNAs. Finally, we identified 40 instances of chromothripsis spanning entire chromosomes or arms that are consistent with micronucleation.

We further analyzed DNA rearrangements related to chromothripsis but restricted this analysis to ancestral chromothripsis shared by three or more samples for which joint rearrangement detection can achieve good accuracy. We identified two examples of chromothripsis involving sub-chromosomal regions (including arms) from multiple chromosomes (Supplementary Fig. 8F, H) that are consistent with multichromosomal bridge resolution. In two instances of chromothripsis, we further identified clustered rearrangement breakpoints near single SCNA boundaries (Supplementary Fig. 8D, H) that resemble the tandem-short-templates rearrangement pattern observed in chromothripsis from bridge resolution^38^ and micronucleation^34^. These rearrangement patterns provide additional evidence supporting the connection between chromothripsis and chromosomal bridges or subsequent micronuclei.

**Fig. 7:**
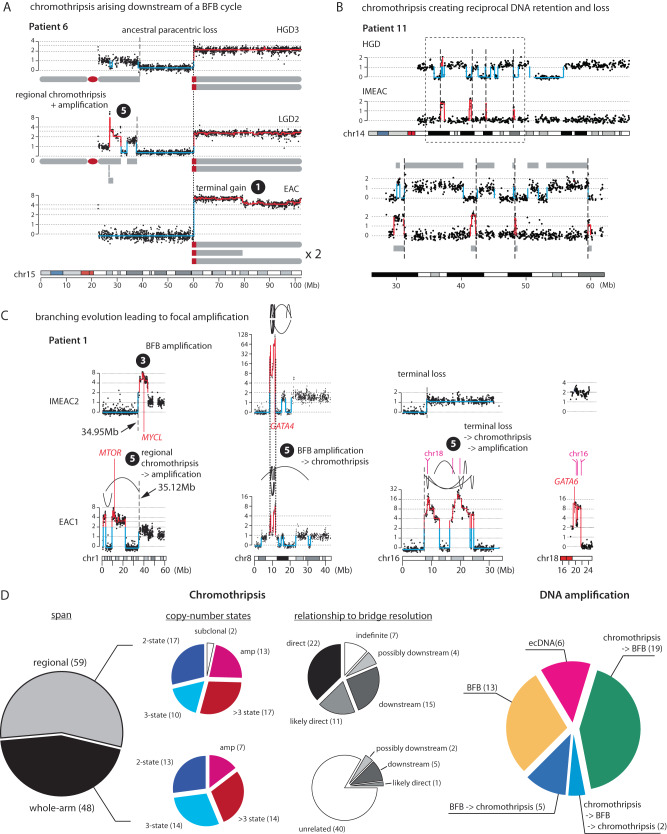
Complex segmental copy-number patterns in BE/EAC genomes indicating successive chromothripsis and BFB cycles. Arabic numbers correspond to the outcomes of different sequences of BFB evolution involving chromothripsis as labeled in Supplementary Fig. 5D. **A** Chromothripsis (in LGD2) and terminal duplication (EAC) occurring downstream of an ancestral paracentric deletion in patient 6. The dotted line represents the ancestral breakpoint shared by all three genomes; dashed lines represent private SCNA breakpoints. **B** Reciprocal distribution of segments of 14q in HGD and IMEAC lesions from patient 11. The bottom shows an enlarged view of the outlined region (dashed box). Except for a small segment near 30 Mb, all the other segments retained in the IMEAC genome are lost from the HGD genome. Dashed lines denote SCNA breakpoints with opposite retention and loss in the two genomes. The retention of the segment near 30 Mb in both genomes may arise from the distribution of DNA fragments from a partially replicated broken chromosome from a micronucleus [Zhang et al.^34^ and Umbreit et al.^38^]. **C** Four subchromosomal regions with distinct high-level DNA amplifications in IMEAC2 (same in IMEAC3) and EAC1 from patient 1. For 8p, we infer the SCNAs evolved from a single unstable ancestor chromosome based on shared SCNA breakpoints (dotted lines). For 1p and 16p, the SCNAs are related by deletions with adjacent boundaries (dashed lines). The amplified regions on 16p in EAC1 are joined to the amplified region on 18q spanning *GATA6*. The order of chromothripsis and amplification is determined based on whether the amplified regions are interrupted by deletions (indicating chromothripsis before amplification) or peppered with DNA losses (indicating chromothripsis after amplification). **D** Summary of chromothripsis and DNA amplification instances grouped by copy-number features and the inferred evolutionary sequences. The inference of chromothripsis arising either directly from or downstream of dicentric chromosome breakage is based on the span of oscillating copy-number pattern relative to entire chromosomes; instances with less certainty are annotated accordingly (“possibly downstream” or “likely direct”). See Supplementary Figs. 8 and 9 for more examples and Supplementary Data 7 for more information.

The comparison of SCNAs in related BE/EAC genomes provides further evidence for BFB cycles in BE genome evolution. In the example shown in Fig. 7A, the ancestral paracentric deletion shared by all three genomes (LGD2/HGD3/EAC) was followed by regional chromothripsis and amplifications near the centromeric break end in the LGD2 clone and a terminal duplication near the telomeric break end in the EAC clone; both downstream alterations likely arose from secondary BFB cycles after the ancestral paracentric deletion. In the example shown in Fig. 7B, the (mostly) non-overlapping segments retained by the HGD and IMEAC genomes are consistent with a random distribution of DNA fragments from a single micronuclear chromosome into a pair of daughter cells^34^. (These patterns could also have arisen from an ancestral chromothripsis followed by distinct downstream deletions.) Other examples of chromothripsis as one of the branching outcomes of BFB cycles are listed in Supplementary Data 6 and Fig. 6E.

The combination of chromothripsis and successive DNA duplications in BFB cycles can generate complex segmental gains and amplifications. Whereas simple BFB cycles generate duplications flanked by large segmental deletions (Fig. 5D and Supplementary Fig. 9A), BFB cycles following chromothripsis generate segmental gains or amplifications with interspersed DNA deletions (Supplementary Fig. 9B). Several copy-number patterns in patient 1 suggest contemporaneous chromothripsis and BFB amplifications (Fig. 7C). On both chr1p and chr16p, the oscillation between DNA deletion and amplification in EAC1 suggests an evolution sequence of ancestral chromothripsis followed by BFB amplifications; the same regions in IMEAC2 display terminal duplications (chr1p) and a simple terminal deletion (chr16p). The presence of adjacent copy-number breakpoints on chr1p and on chr16p between the EAC1 and IMEAC2 genomes are most parsimoniously explained as divergent evolutionary outcomes of a single ancestral broken chromosome. Interestingly, the amplified regions on 16p in the EAC1 genome do not contain known oncogenes but are co-amplified with a region on 18q containing *GATA6*, a recurrently amplified EAC oncogene. By contrast, the IMEAC2 genome harbors neither amplification but has more amplified *GATA4* on chr8p. Moreover, the shared boundaries of amplified regions on 8p in both EAC1 and IMEAC2 indicates that the *GATA4* amplification was ancestral to both genomes but underwent different downstream evolution. The distinct *GATA4* and *GATA6* amplifications in these two genomes, likely reflective of positive selection for their combined expression^54^, highlights how persistent chromosomal instability can rapidly generate copy-number heterogeneity and fuel the acquisition of oncogenic amplifications.

As DNA amplification is only one out of many possible outcomes of multigenerational copy-number evolution (we operationally defined focally amplified regions to have allelic copy number ≥ 8 that can be attained with at least three rounds of duplications), clonally fixated amplifications are likely reflective of positive selection and expected to contain oncogenes. Among 45 focally amplified regions each spanning one or multiple loci on a chromosome (Supplementary Data 7), 24 encompass putative oncogenes and 29 overlap with regions that are recurrently amplified in cancer. The significance of focal amplification as a mechanism of oncogenic activation during EAC transformation^30,31^ is further supported by the observations of recurrent amplifications of EAC oncogenes, including *ERBB2* on 17q (5/15 patients) (Supplementary Fig. 9A, B) and *GATA6* on 18q (4/15 patients), distinct amplifications in different cancers from the same patient (Supplementary Fig. 9C, D), and sporadic oncogene amplifications that are exclusive to cancer lesions but not their precursors, including *IGF1R* (patient 3), *MET* (patient 4, Fig. 6C), and *KRAS* (patient 10 and Supplementary Fig. 9E). Notably, amplification can be either intra- or extra-chromosomal (Supplementary Fig. 9C,D) and can be clonally present even in non-dysplastic BEs after p53 loss (patient 6 and Supplementary Fig. 9F).

In summary, we found that many complex segmental copy-number alterations in BE/EAC genomes, including focal amplifications, can be deconvoluted into different evolution sequences of sequence duplications generated by BFB cycles and chromothripsis from DNA fragmentation (Fig. 7D). Together with observations of terminal/internal SCNAs reflecting simple copy-number outcomes of BFB cycles, these data provide in vivo evidence for the involvement of abnormal nuclear structures including micronuclei^34–36^ and chromosome bridges^37,38^ in the generation of EAC genome complexity.

### Chromosomal instability generates continuous copy-number variation prior to discrete changes

Our analysis of BE/EAC genomes reveals both copy-number complexity and copy-number heterogeneity in BE subclones that indicate multigenerational evolution of unstable chromosomes. Importantly, copy-number variation in single BE cells should precede copy-number complexity in BE subclones. We wondered whether such heterogeneity in single BE cells can be discerned prior to copy-number complexity in BE subclones.

**Fig. 8:**
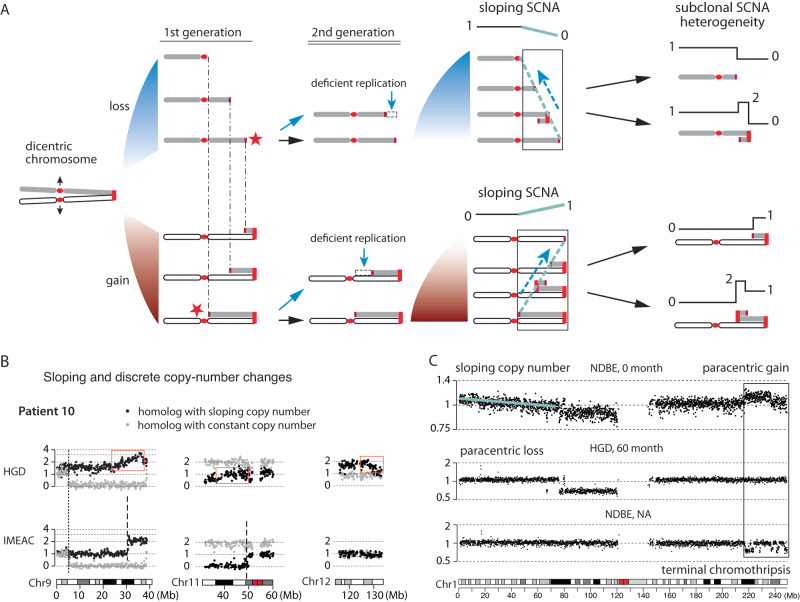
Chromosomal instability creates copy-number heterogeneity prior to copy-number complexity. **A** Successive BFB cycles can generate progressive DNA losses at the broken ends of chromosomes resulting in a gradual attenuation (sloping) of DNA copy number towards either telomeric (top) or centromeric (bottom) boundaries. Individual broken ends in single cells may acquire terminal duplications that become visible after clonal expansion, but the population average will accrue DNA loss due to deficient DNA replication. **B** Sloping DNA copy number on Chrs.9, 11, and 12 (black dots) in the HGD sample from patient 10. The constant DNA copy number of the other homolog is shown in gray. In the regions of sloping copy-number variation on Chrs.9 and 11 in HGD, we observe clonal copy-number changepoints in IMEAC, suggesting clonal expansion of a subclone/single cell in the HGD sample. **C** Copy-number evolution revealed in longitudinal BE sequencing data published by Killcoyne et al. (2020)^55^. In this patient (patient 86), the NDBE sample in the first biopsy (0 month) displays sloping (1p terminus) and subclonal (1q terminus) copy-number variation. A subsequent biopsy with HGD (at 60 months) from the same patient shows a (sub)clonal paracentric loss on 1p with regional copy-number oscillation near the telomeric boundary; another NDBE lesion (timing unspecified) shows chromothripsis at the 1q-terminus in the same region of subclonal copy-number gain in the NDBE lesion at 0 month. Both examples indicate copy-number heterogeneity in the ancestral NDBE lesion. See Supplementary Fig. 11 for additional examples.

If chromosome breakage only generates reciprocal DNA retention and loss between sibling cells, such changes are not visible at the clonal level as there is not net DNA gain or loss. However, we previously demonstrated that chromosomes in both micronuclei and bridges undergo deficient DNA replication leading to net DNA losses^34,38^. If broken chromosomes remain mitotically unstable for multiple generations, successive under-replication of the broken termini can generate varying terminal losses in the progeny population that result in ‘sloping’ copy number variation (Supplementary Fig. 7 and Fig. 8A). We identified sloping copy-number variation on three chromosomes in the HGD sample from patient 10 (Fig. 8B). The constant DNA copy number of the intact homolog (gray) establishes that the sloping copy-number pattern reflects genetic variation instead of technical variability (e.g., due to FFPE DNA degradation). Moreover, the observation of clonal (‘discrete’) copy-number changes on both chr9 and chr11 in the IMEAC genome within the same regions of sloping copy number in HGD suggests that the IMEAC ancestor was a subclone of HGD. Although the IMEAC genome does not show clonal copy-number alterations on 12q that would have been derived from an HGD subclone with varying 12q loss, it contains a high-level amplification spanning *KRAS* on the 12p arm (Supplementary Fig. 9E). The amplification was inferred to have originated from the same parental chromosome with sloping copy number variation on the 12q-terminus in HGD. It is tempting to speculate that the *KRAS* amplification had evolved from an unstable chr12 missing the q-terminus by chromothripsis and subsequent duplications.

To further explore the possibility that sloping copy-number variation in early-stage BE samples precedes clonal SCNAs in late-stage BE subclones, we analyzed the sequencing data of longitudinal BE samples released in a recent study^55^ (Supplementary Fig. 10A). We first confirmed the presence of large segmental SCNAs in both non-dysplastic and dysplastic BE samples prior to transformation and the presence of distinct copy-number alterations in aneuploid BE or early cancer clones indicating copy-number evolution (Supplementary Fig. 10B, 11 and Supplementary Data 8). The observation of frequent copy-number evolution in longitudinal BE samples provides orthogonal evidence of persistent chromosomal instability in BE cells that complements the observation of widespread copy-number heterogeneity in multifocal BE samples. We further identified sloping copy-number variation in 9 patients. (Due to the limited sequencing depth, this inference was based on total DNA sequence coverage instead of haplotype-specific coverage.) In patient 86, we observed sloping copy-number variation on the 1q arm in the NDBE sample indicating varying terminal gains (Fig. 8C, top); the same region shows a clonal terminal retention in a late-stage HGD sample (Fig. 8C, middle). In contrast to the sloping DNA copy number of 1p, the 1q arm contains a subclonal paracentric gain that may be related to the chromothripsis at the same 1q-terminal region in another NDBE lesion (Fig. 8C, bottom). Together, the observations in both longitudinal and multifocal BE samples suggest ongoing evolution of unstable BE genomes prior to the emergence of EAC clones. As sloping copy-number variation precedes clonal SCNAs, it may ultimately serve as a prognostic marker of BE progression or ongoing genome instability.

## Discussion

We here studied precancer genome evolution in a unique cohort of incipient esophageal adenocarcinomas and adjacent Barrett’s esophagus lesions by haplotype-specific copy-number analysis. We identified recurrent copy-number evolutionary patterns related to both gross karyotype changes and complex segmental alterations including focal amplifications that indicate continuous genome instability in BE cells.

We find that arm-level copy-number changes often accumulate in episodic bursts and are consistent with the outcome of whole-genome duplication (WGD) with downstream events including multipolar cell division and micronucleation^32,33^. WGD is frequently followed by extensive chromosome losses, giving rise to highly aneuploid genomes, but can also generate near complete genome duplication. For example, the EAC genome in patient 7 is a near complete duplication of the LGD2 genome (with odd copy-number states on 4q, 5, and 9q indicating post-WGD losses); the D5 cell in the single-cell collection is close to a complete duplication of the F12 cell (with odd copy-number states on 2p, 9q and post-WGD gains of 17q and 18p). When and how duplicated genomes re-establish stable karyotypes in vitro and in vivo require further investigation.

We find several patterns of segmental copy-number alterations in BE/EAC genomes that are consistent with an origin from dicentric chromosome breakage and evolution^38^. These include simple segmental copy-number gains and losses consistent with the outcome of a single BFB cycle (Fig. 5), compound copy-number gains consistent with successive BFB cycles (Fig. 6A-C), and distinct copy-number alterations to a single parental chromosome in related BE/EAC genomes that are consistent with copy-number variation generated by multigenerational BFB cycles (Fig. 6C-E). The mechanistic association between BE/EAC genome complexity and BFB cycles is further supported by the presence of regional or arm-level chromothripsis (Fig. 7A, C and Supplementary Fig. 8), interchromosomal translocations (Fig. 6A, B and Supplementary Fig. 8F, H), and tandem-short-templates rearrangements (Supplementary Fig. 8F, H), all of which were previously identified in vitro^37,38^. Finally, the patterns of progressive DNA deletions (Fig. 6D) and sloping copy-number variation (Fig. 8B, C) provide strong evidence for ongoing BFB cycles^38^ in BE cells. This pattern of polyclonal copy-number variation may be regarded as a signature of ongoing or ‘present’ genome instability that precedes clonal SCNAs that indicate ‘past’ genome instability (Fig. 8A).

We observe nearly ubiquitous bi-allelic *TP53* inactivation preceding the emergence of aneuploid BE cells or BE clones. This result reinforces prior observations in BE cells^50^ or from comparative studies of BEs and late EACs^11,30,31,56^. However, cells with intact p53 do occasionally acquire large copy-number alterations. This is demonstrated by the observation of infrequent arm-level or large segmental SCNAs in single BE cells (Fig. 3) and even instances of chromothripsis in BE clones (e.g., on chr9p in patient 8 BE1-3, patient 11 LGD, and patient 6, all samples) inferred to have occurred prior to *TP53* inactivation. In comparison to BE cells with intact p53, the most distinguishing features of p53-null BE cells include (1) massive aneuploidy including whole-genome duplication; and (2) complex segmental gains (with copy-number states above two) that require multiple generations of chromosome breakage and recombination. This observation suggests that the dominant tumor suppressive mechanism of p53 may be the suppression of cell proliferation after chromosome missegregation^44^.

**Fig. 9:**
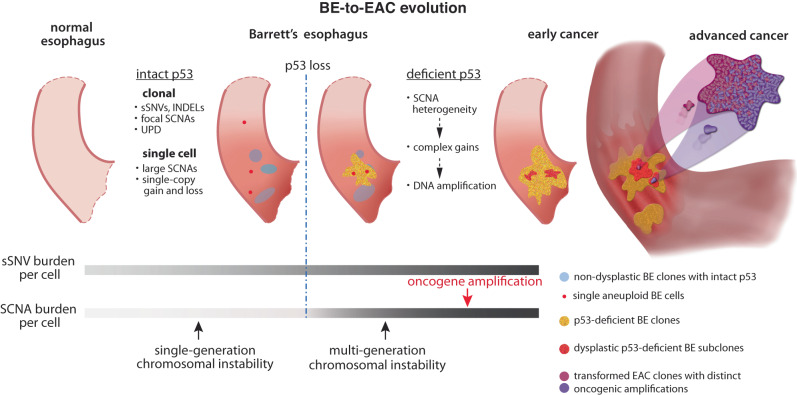
Evolutionary dynamics of local sequence changes (single-nucleotide mutations, short sequence deletions/duplications) and chromosomal structural aberrations during esophageal cancer evolution. Prior to p53 loss, the suppression of cell division after chromosome missegregation events or the acquisition of DNA damage inhibits clonal expansion of chromosomal structural alterations; therefore, only alterations that neither result from nor lead to chromosome missegregation or instability (local sequence changes, focal deletions/duplications, or uniparental disomies) are detectable at the clonal level. After p53 loss, there is a rapid increase of SCNA burden per cell that is due to both the clonal expansion of ancestral SCNAs generated by chromosome missegregation and secondary SCNAs accumulated during the downstream evolution of unstable chromosomes, the latter generating both copy-number heterogeneity and DNA duplications. Although the average mutational burden per cell (of both local and structural alterations) and the total genetic diversity of the tumor clone continue to increase during cell proliferation, the acquisition of cancer drivers can cause clonal dominance or sweep that make minor subclones harder to detect by bulk or even single-cell sequencing. Therefore, analyses of precancer lesions with limited clonal expansion can reveal ancestral genetic heterogeneity that may be undetectable in advanced cancers.

The abrogation of p53-dependent cell cycle arrest after chromosome missegregation has two implications (Fig. 9). First, arm-level or large segmental SCNAs generated by chromosome missegregation events can undergo clonal expansion and become visible at the clonal level. Second, and more importantly, it allows single-cell division errors such as whole-genome duplication or chromosome bridge formation to precipitate multigenerational instability that both generates copy-number heterogeneity and fuels the acquisition of oncogenic amplifications. Therefore, even without an apparent increase in the rate of events that generate unstable chromosomes, p53 loss marks the onset of rapid accumulation of copy-number heterogeneity and complexity that contrasts with continuous SNV accumulation. This explains the significant differences between SCNAs in ageing esophagus or BEs with intact p53 and in BEs with deficient p53. Interestingly, we observed a distinct pattern of copy-number variation in BE cells with intact p53 reflecting uniparental disomy (UPD) alterations with varying boundaries (Fig. 3E). How large segmental UPDs arise in mammalian cells is unknown. The similarity of progressive DNA breakpoints in varying UPDs to those in progressive DNA losses (Fig. 6D) suggests that these two patterns may reflect different DNA repair outcomes of broken chromosomes generated by successive BFB cycles (Supplementary Fig. 4). If this model were true, it further implies that cells with intact p53 do tolerate certain types of chromosomal instability but raises the question of how p53 or other selection factors impact the rearrangement outcomes of such instability.

The early onset of genome instability during BE progression revealed in our analysis challenges the prevailing view that chromosomal aberrations are exclusive to advanced cancers or only arise late during tumor development. Analyses of advanced tumors by either bulk^5^ or single-cell^57^ sequencing usually reveal only truncal or late subclonal alterations with relatively late divergence of different cancer subclones. As late-stage cancers are often dominated by the most aggressive clones, analyses of late-stage cancers cannot reveal copy-number heterogeneity in single cells prior to transformation. By contrast, genetic diversity is more visible in precancerous lesions due to the lack of dominant clones. This explains the observation of significant copy-number differences in multifocal BE clones (Fig. 2), copy-number evolution in longitudinal BE samples (Supplementary Figs. 10 and 11), and sloping copy-number variation in single BE lesions (Fig. 8B, C). Moreover, the generation of complex copy-number gains, including focal amplifications, necessitates multigenerational chromosomal instability that invariably creates copy-number heterogeneity (Figs. 3, 6, and 7). Therefore, complex segmental gains in EACs or dysplastic BEs can be regarded as a signature of ‘past’ chromosomal instability in their ancestor cells.

Oncogenic amplifications are a hallmark of advanced EACs. Our analyses demonstrate that these events are present in both early EACs and BEs with deficient p53 (Figs. 2 and 3, and Supplementary Fig. 9E). We further identified distinct oncogenic amplifications in different dysplastic BEs or early EACs from the same patient (Figs. 2 and 7C), some of which were associated with independently transformed EAC foci. Of clinical importance, we identified multiple examples where there were two or more independent transformations into cancer, each containing unique oncogene amplifications. As the independent EAC clones may grow into each other to form a single tumor mass or seed different metastatic lesions, both intratumor and primary/metastasis oncogenic amplification heterogeneity that is seen in advanced EAC^58^ may be the inherent outcome of chromosomal instability after p53 loss that could have been initiated in precancer BE cells and persist after transformation (Fig. 9).

Our model of chromosomal-instability driven copy-number evolution makes several predictions. First, segmental copy-number complexity at the clonal level is preceded by copy-number heterogeneity at the single-cell level. This is demonstrated in our study (Figs. 3 and 8) but should be further tested by single-cell DNA sequencing of precancerous or ageing tissues. Second, p53 loss enables the accumulation of copy-number heterogeneity in precancer lesions that may differ from late-stage cancers due to the lack of clonal sweep. This prediction can be tested in other cancers with early p53 inactivation and precursor conditions, including serous ovarian cancers^16^, basal breast cancers, uterine serous endometrial cancers, pancreatic cancers^59^, and colitis-associated colorectal cancers^15^. Finally, our analysis of SCNAs in BE/EAC genomes suggests a mechanism-based classification of copy-number patterns (Fig. 3 and Supplementary Figs. 3,4,5,8). Extending this analysis to cancers both with and without *TP53* inactivation will generate new knowledge of tumor evolution dynamics with both diagnostic and therapeutic implications.

## Methods

### Sample identification, DNA extraction, and sequencing data generation

#### Sample identification

Formalin-fixed paraffin-embedded (FFPE) endoscopic mucosal resections or esophagectomy samples were identified in the pathology archives of Brigham and Women’s Hospital, the Mayo Clinic, or the University of Pittsburgh Medical Center. Both sample identification and sequencing were performed with documented informed consent and IRB approval from Brigham and Women’s Hospital, the Mayo Clinic, and the University of Pittsburgh Medical Center. The sequencing, computational analysis, and data deposition follow a study protocol established at the Broad Institute that complies with all relevant ethical regulations. Patients having received either chemo/radiotherapy or endoscopic ablation prior to resection were excluded. Hematoxylin and eosin (H&E) stained slides were reviewed by two gastrointestinal pathologists (M.S. and A.A.) to determine consensus areas of Barrett’s Esophagus (BE), BE with low-grade dysplasia (LGD), BE with high grade dysplasia (HGD), and esophageal adenocarcinoma (EAC). (See Supplementary Fig. 1 for examples.) In cases of uncertainty of pathological classification, the samples were reviewed by a third pathologist (R.O.). Any sample without a consensus diagnosis was excluded from further analysis.

#### DNA isolation and sequencing library construction

Ten 4μm sections from each FFPE block were cut sequentially onto PEN membrane frame slides (Life Technologies, Grand Island, NY) bracketed by standard slides for H&E staining. The frame slides were stained using Arcturus paradise plus stain (Life Technologies) following the manufacturer’s recommendations. The areas of interest were microdissected using the ArcturusXT laser capture microdissection Instrument (Life Technologies). When dissecting normal tissue that was used as germline reference, we avoided epithelial tissue that may contain BE or EAC cells.

DNA was isolated using the Promega (Madison, WI) FFPE DNA isolation kit following the manufacturer’s protocol with the exception that the tissue was digested with proteinase K overnight. DNA was quantified using Picogreen dsDNA Quantification Reagent. Approximately 50 ng of genomic DNA was fragmented by sonication (Covaris) to 250 bp and further purified using Agentcourt AMPure XP beads. Whole-genome DNA libraries were constructed from size-selected DNA using KAPA HTP Library Preparation Kit (Roche) and subjected to low-pass whole-genome sequencing (~0.1x). Samples with sufficient library complexity (i.e., estimated total number of unique sequencing fragments ≥ 100 million) were selected for deeper whole-genome sequencing (20-30x).

#### Sequencing data generation and processing

Multiplexed whole-genome sequencing (WGS) libraries were sequenced on NovaSeq6000 or HiSeq2500 instruments (Illumina) in paired-end mode (2 x 150 bp). Sequencing reads were aligned to the NCBI Human Reference Genome Build GRCh37/hg19 using bwa (version 0.7.7). Aligned reads were processed using the standard pipeline established by the Genomics Platform at the Broad Institute, including base-quality score recalibration, duplicate removal, and realignment near indel variants as described in the GATK best practice (https://gatk.broadinstitute.org/hc/en-us/articles/360035535912).

### Generation of single-cell sequencing data

#### Cell sorting

Cells were harvested by endoscopic cytology brushing from a region of high-grade dysplasia and then pelleted in a falcon conical tube (Stem Cell) after trypsin digestion and washing with Dulbecco’s phosphate-buffered saline (DPBS, Gibco). Freshly prepared, cold 70% ethanol (5 ml) was added drop-wise while vortexing to fix and preserve cells at -20^o^C. Cells were stained by DAPI (Life Technologies) and underwent fluorescence-activated sorting (FACS) into a skirted RNase, DNase-free 96-well plate (Eppendorf) with 5 µl DPBS added to each well before sorting. During sorting, the first (A1) well was left empty and the last well (H1) contained 100 sorted cells, both serving as controls for single-cell genome amplification. Each plate after sorting was immediately sealed and placed on dry ice before transferred to −80 °C storage.

### Library construction

Single-cell lysis and whole-genome amplification was performed using the REPLI-g Single Cell kit (Qiagen) with the following modifications: Due to having 5 µl of instead of the standard 4 µl starting solution, we added 3.5 µl (instead of 3 µl) of Buffer D2 for cell lysis and 3.5 µl (instead of 3 µl) of Stop Solution to terminate cell lysis. During genome amplification, we added 7 µl kit water instead of 9 µl into the master mix (38 µl master mix was added into each well) to match the total volume at the end of reaction. Amplified DNA was purified with ethanol and quantified by Qubit dsDNA HS Assay kit (Life Technologies). About 100 ng amplified DNA was sheared to ~350 bp DNA fragments (Covaris sonication) and processed with a KAPA HTP Library Preparation Kit (KK8234, KAPA Biosystem) for multiplexed Illumina sequencing library construction.

### Quality assessment and sequencing of single-cell libraries

A total of 95 whole-genome amplified DNA libraries (94 single cells and one 100-cell sample) were screened by low-pass MiSeq sequencing, from which we identified 24 cells with discernable arm-level copy-number changes and 45 cells with close to diploid coverage. The remaining samples showed poor coverage uniformity and were discarded. Aneuploid cells (24 total), diploid cells (45 total), and a 100-cell sample were pooled and sent for paired-end sequencing (50 bp x 2) on the NovaSeq6000 platform (S2 kit) to yield 2.9 billion read pairs (2.15 billion aligned), or ~1x mean coverage per cell. The sequencing data were aligned to the GRCh38 reference by bwa.

### Detection and filtering of somatic and germline single-nucleotide variants

#### Mutation detection with Mutect2

We first performed short variant discovery in each BE/EAC sample using GATK Mutect2 (version 4.0.1.2) and the matching germline reference as control. To filter false variants due to recurrent alignment errors, we used a ‘reference’ panel of variants detected in 125 blood samples:

gs://fc-16adb3e5-7c0a-4805-aa5e-374b579d03e1/wgs_hgx19_125_cancer_blood_normal_panel.vcf

To filter rare artifacts and germline variants that were missed in the matching germline reference, we built a germline resource consisting of >10,000 genomes from gnomAD (version 2.0.2). To remove low-confidence variants, we applied the following downstream filters: 8-oxoguanine (OxoG) artifacts, FFPE artifacts, and alignment artifacts due to sequence similarity between two or more regions in the genome.

Commands for filtering OxoG and FFPE artifacts:


gatk FilterByOrientationBias \ -V filtered.vcf.gz \ --artifact-modes 'G/T' \ -P tumor_artifact.pre_adapter_detail_metrics.txt \ -O oxog_filtered.vcf.gz


Commands for filtering alignment artifacts:


gatk FilterAlignmentArtifacts \ -R hg19.fasta -V somatic.vcf.gz \ -I somatic_bamout.bam \ --bwa-mem-index-image hg38.index_image \ -O filtered.vcf.gz


The filtered variants were annotated using Oncotator (version 1.9.9.0). We genotyped mutations detected in individual samples across all samples from each patient by running Mutect2 in the GENOTYPE_GIVEN_ALLELES mode. We considered the mutant allele to be present in a sample if there were at least three variant-supporting reads; we then used the genotype data to calculate the pairwise similarity between samples that is shown in Supplementary Fig. 2.

#### Joint variant detection by HaplotypeCaller

We used GATK HaplotypeCaller (v.4.0.12.0-6) to detect both germline heterozygous variants and somatic variants jointly from all samples from each patient. To filter false variants due to recurrent alignment errors, we imposed the following read filters:

--minimum-mapping-quality 30 (excluded reads having low mapping quality)

--read-filter MateOnSameContigOrNoMappedMateReadFilter and

--read-filter MateDifferentStrandReadFilter (discordant alignment positions)

--filter-too-short 25 (excessive clipping)

--read-filter OverclippedReadFilter (over soft-clipping)

--read-filter GoodCigarReadFilter (bad CIGAR string)

--read-filter AmbiguousBaseReadFilter (>5 percent of N bases in the sequence).

We selected only bi-allelic variants and further removed variants in low-complexity DNA sequences (https://raw.githubusercontent.com/mskcc/ngs-filters/master/data/rmsk_mod.bed), poorly mappable regions of the genome (https://raw.githubusercontent.com/mskcc/ngs-filters/master/data/wgEncodeDacMapabilityConsensusExcludable.bed), or within 100 base pairs of INDEL, multinucleotide changes, or other variants (bcftools filter --SnpGap 100:indel,mnp,other,overlap).

To select heterozygous variants for haplotype-specific copy-number calculation, we imposed the following criteria on biallelic SNVs detected by HaplotypeCaller from all samples (both germline reference and BE/EAC) in each patient: (1) variant sites were among common polymorphisms in the 1000 Genomes Project Phase 3 reference haplotype panel (only these variants were used for statistical haplotype phasing); (2) at least one sample showed the heterozygous genotype (‘0/1’); (3) at least two samples showed more than two reads of the alternate genotype; (4) at least two samples showed more than two reads of the reference genotype. We further excluded variants in autosomes that were heterozygous in >50% of samples in our cohort (8/15) based on the expectation that the frequency of heterozygotes in a population following the Hardy-Weinberg equilibrium should be <50%. All these filters served to remove homozygous variants that appeared to be heterozygous due to sequencing errors, alignment errors, or technical artifacts in FFPE libraries.

To improve the detection sensitivity of cancer gene mutations, we also ran HaplotypeCaller on the cancer gene consensus (https://cancer.sanger.ac.uk/census) plus three genes (*GATA4*, *GATA6*, *VEGFA*) that are recurrently altered in esophageal cancers. This analysis revealed recurrent loss-of-function mutations in *TP53*, *CDKN2A*, *ARID1A*, *ARID1B*, and *SMARCA4* that are annotated in Fig. 2.

### Standard copy-number analysis and estimation of sample purity and ploidy

We performed standard somatic copy number analysis using the GATK4 Somatic CNV ModelSegments pipeline (version 4.0.1.2). Briefly, read counts were collected in 5 kb genomic intervals, normalized to fractional coverage, and then corrected for GC-dependent bias. Recurrent coverage bias in FFPE libraries was then normalized using the sequence coverage of germline samples in our cohort as a reference panel. The normalized total sequence coverage and allelic ratio (estimated from allelic depths at heterozygous variant sites) were used as input to ModelSegments for smoothing and segmentation with the following changes to default parameters. To filter out low-quality data points, we increased ‘minimum-total-allele-count’ to 50 (default: 30); to avoid over-segmentation, we increased ‘number-of-changepoints-penalty-factor’ to 1.8 (default: 1.0). We further calculated average normalized sequence coverage in 25 kb genomic intervals for haplotype-specific copy-number analysis.

We estimated the clonal fraction (‘purity) and average DNA copy number (‘ploidy’) of aneuploid BE/EAC clones using ABSOLUTE (version 1.5). Input data to ABSOLUTE include: (1) normalized read depth and allelic ratio in 5 kb bins; (2) segmented copy ratio; (3) allelic frequency of somatic mutations. We manually reviewed all candidate solutions generated by ABSOLUTE to pick the optimal solution with the fewest subclonal (non-integer) copy-number states. In selecting the most likely solution, we further took into consideration the tumor cell fraction assessed from histopathological analysis. The purity and ploidy estimates were later validated independently by haplotype-specific sequence coverage. BE samples without large SCNAs were excluded from purity/ploidy estimates: Their phylogeny was inferred from sSNVs or small focal SCNAs (patient 1: COLME and BE; patient 4: COLME; patient 5:BE; patient 7:COLME). An exception was the HGD1 sample in patient 9. This sample contained no large segmental SCNAs but several regions of focal amplification: The amplified copy number was most likely due to tumor cells from the adjacent IMEAC2 lesion (see Fig. 2), which was supported by the lack of amplified DNA in HGD1 from cytogenetic analysis (Supplementary Data 7). The HGD1 sample was placed next to the IMEAC2 sample in the phylogenetic tree based on this feature.

### Haplotype-specific copy number analysis

The idea of using haplotype information to improve the accuracy of allelic fraction calculation was previously implemented for SNP array data^60^, whole-genome sequencing^61^, and whole-exome sequencing^62^. Our haplotype-specific copy-number analysis workflow (Supplementary Figs. 12) combines statistical phasing (Supplementary Fig. 13) and allelic-depth-based phasing (Supplementary Figs. 14) to extend the range of haplotype inference and SCNA phasing to entire chromosomes (or arms). The ability to identify somatic copy-number alterations on each parental chromosome (Supplementary Figs. 15 and 16) further enables us to determine the relationship between SCNA breakpoints (Supplementary Fig. 17**)** and relate copy-number evolution patterns in BE/EAC genomes to the copy-number outcomes of unstable chromosomes.

#### Identification of polymorphisms on parental chromosomes

The identification of heterozygous variant sites on parental chromosomes was described in “Joint variant detection by HaplotypeCaller” section. Because BE/EAC samples also contain DNA from normal cells, joint variant detection from both BE/EAC samples and the matching germline reference achieves better variant detection sensitivity than variant detection solely from the germline reference, especially for germline samples with low sequencing coverage (<15x in patient 8-11). The joint detection strategy consistently revealed 1.5-1.7 million common heterozygous variants (identified in the 1000-genome project cohort) in all 15 patients and 1.1-1.3 million variants in each individual sample (Supplementary Data 1). The high density of heterozygous variants (~1 per 3 kb) ensures the accuracy of allelic copy-number calculation.

#### Statistical phasing of parental haplotypes

The heterozygous genotypes in each patient were uploaded to the Sanger Imputation Server for statistical phasing using EAGLE2^39^ (version 2.0.5) and reference haplotype data from the 1000-Genome Phase 3 release (https://www.internationalgenome.org/data-portal/data-collection/phase-3). Although EAGLE2 can directly perform statistical phasing using both heterozygous and homozygous genotypes, based on benchmarking using reference haplotype data^63^, we found that the haplotype phase calculated using only heterozygous genotypes was slightly more accurate than the haplotype phase inferred from both heterozygous and homozygous genotypes. We therefore used the haplotype derived from statistical phasing applied to only high-confidence heterozygous variant sites.

#### Haplotype-specific DNA copy number calculation

A detailed presentation of the rationale, algorithmic implementation, technical benchmarking, and validation can be found in Supplementary Information.

#### SCNA Classification and evolutionary inference

We classified SCNAs on each parental chromosome based on the number of SCNA breakpoints and copy-number states. See Supplementary Fig. 4 for the criteria and examples for each SCNA category. SCNAs affecting the same parental chromosome in different samples were manually reviewed to determine their evolution history. SCNA breakpoints in two or multiple samples that were within 0.1 Mb from each other (to account for segmentation inaccuracy) and associated with the same type of copy-number change (either gain or loss) were classified as identical. SCNA breakpoints within 0.1 Mb but associated with opposite copy-number changes (i.e., copy-number gain in one sample and loss in another sample) were classified as complementary. Individual SCNAs (including complex SCNAs with multiple breakpoints) were classified as shared between two samples if all SCNA breakpoints were identical. If only a subset of SCNA breakpoints were identical, the SCNA patterns were classified as branching (i.e., initiated by a single ancestral event but having different downstream changes). Branching evolution also included examples where distinct SCNA breakpoints on the same parental chromosome can be explained by sequential or progressive DNA alterations (Fig. 6D and Supplementary Fig. 7).

The timing of SCNA relative to duplication events (both whole-chromosome and whole-genome) was determined as follows. SCNAs with more than one copy difference across changepoints were assumed to have arisen before duplication; SCNAs with single-copy changes were assumed to have arisen after duplication. For SCNAs identified in samples inferred to have undergone whole-genome duplication (WGD), their timing was further validated based on their presence or absence in related samples from the same patient without WGD acquisition. For chromosomal or arm-level SCNAs, if the final copy-number state was 1, they were assumed to have been first duplicated to two copies and then undergone whole-chromosome loss; if the final copy-number state was an odd number above 1 (3,5,…), they were assumed to have first undergone duplication to the nearest even copy-number state and then undergone either a single-copy gain or a single-copy loss, depending on the number of ancestral WGDs.

### SCNA and SNV-based phylogenetic inference

Phylogenetic inference was done independently from SCNAs and SNVs. For SCNA-based phylogenetic inference, we used haplotype-specific SCNA breakpoints as lineage markers as the breakpoints remain unaltered by downstream whole-chromosome or whole-genome duplication events. The phylogenetic tree was constructed based on the presence or absence of SCNA breakpoints shared by two or more samples. Arm-level or whole-chromosome SCNAs were also considered where there were no shared internal SCNA breakpoints. All phylogenetic trees were manually reviewed to ensure consistency and exclude confounding factors due to (1) subclonal mixture between different samples; and (2) whole-chromosome/arm-level deletion that eliminate ancestral copy-number breakpoints.

For SNV-based phylogenetic inference, we calculated genetic similarity as the percentage of shared sSNV variants between two samples normalized by the total number of sSNVs detected in each sample. The sSNV similarity was largely consistent with the SCNA-derived phylogeny with the following discrepancies: (1) HGD lesion in patient 1; (2) the lineage of HGD2, IMEAC2, IMEAC1 in patient 12; (3) the lineage of all samples in patient 15. Evidence supporting the SCNA-derived phylogeny in patients 12 and 15 was presented in Supplementary Fig. 17; in both cases, the sSNV similarity was less accurate due to false negative sSNV detection. For the HGD lesion of patient 1, the dominant clone in HGD was inferred to have undergone whole-genome duplication and contain a missense mutation in *TP53* that was shared with the cancer lesions. Presumably, the HGD lesion was a polyclonal mixture of cells that were similar to BE/COLME (based on the SNV burden) and cells that were similar to EAC1/IMEAC2/IMEAC3; the lesser similarity between HGD and the cancer lesions was also because the cancer lesions had both acquired more de novo mutations and lost ancestral mutations in their evolution from the common ancestor with HGD.

### Somatic rearrangement detection

We performed joint somatic rearrangement detection on all samples from each patient using SvABA (version 1.1.3). To improve detection sensitivity, we decreased ‘mate-lookup-min’ to 1 (default: 3) and ‘min-overlap’ to 25 bp (default: 0.4 x read length). We eliminated rearrangements with either breakpoint overlapping with known germline variants, blacklisted regions, or regions of low sequence complexity (https://data.broadinstitute.org/snowman/Submission/hg19.svaba.exclude.bed). To eliminate false rearrangements due to chimeric sequences generated in FFPE library construction, we first excluded rearrangements with breakpoints within 100 kb and then only included rearrangements with both breakpoints within 100 kb from copy-number changepoints. The copy-number filtering strategy inevitably removed true rearrangements without apparent copy-number changes but was necessary due to the high false positive rate (due to chimeric sequences) and low sensitivity (due to DNA degradation) of rearrangement detection in FFPE libraries. (See Supplementary Fig. 6 for snapshots of random chimeric sequences near true rearrangements validated by copy-number breakpoints). We therefore restricted the analysis to SCNA-related rearrangements.

### Fluorescence in situ hybridization analysis

After deparaffinization and dehydration, FFPE tissue sections were first digested in 0.1 N HCl for 20–30 min and then washed in phosphate-buffered saline (PBS) solution for 5 minutes at room temperature. Bacterial artificial chromosomes (BAC) probes against centromeric sequences (*CEN3*, *CEN4, CEN5, CEN8, CEN10, CEN11, CEN11q, CEN12, CEN13, CEN18, CEN22, CEN17*) and amplified oncogenes (*ERBB2, MYC, EGFR, KRAS, VEGFA*, and *FGFR2*) were fluorescently labeled (Chromosomescience laboratory, Sapporo, Japan). After dehydration and drying, each FISH probe was applied to each targeted area of tissue. The slides were sealed with coverslips, denatured at 90 °C for 10 minutes, and then followed by overnight hybridization at 37 °C in a wet chamber. Hybridized slides were washed in 2x saline-sodium citrate (SSC) buffer for 5 minutes and coverslips were removed gently. The slides were washed in 50% formamide/2x SSC for 20 min at 37 °C, and then kept in 1x SSC for 15 min at room temperature. The slides were counterstained with 4′,6-diamidino-2-phenylindole (DAPI). The FISH images were captured with a fluorescence microscope (BZ-X710, Keyence, Japan). The number of gene probes and corresponding centromeric probes were then manually quantified.

### Single-cell sequencing analysis

#### Calculation of total DNA copy number

We calculated total DNA copy number from the sequence coverage of each cell in four steps. (1) Read counts were calculated in 10 kb intervals and centered by the genome-wide mean. (2) The average read coverage (10 kb) in each sample was then normalized for recurrent coverage bias estimated using the median coverage across all samples. (3) GC-dependent coverage variation was normalized based on %GC in 100 kb intervals. (4) The 10 kb normalized sequence coverage was averaged over 100 bins (1 Mb) to generate local sequence coverage.

When performing step (2) above, we needed to first select a region with constant DNA copy number in each cell. We either picked the largest chromosome arm with median coverage close to the genome-wide median of arm-level median coverage (absolute deviation less than 0.05) or picked the arm with the lowest standard deviation of coverage when no arm was close to the genome-wide median (e.g., highly aneuploid genomes). Normalization of recurrent coverage bias was performed on log-transformed sequence coverage. The (log-transformed) sequence coverage in the selected region of constant DNA copy number was fitted to a cubic polynomial function of the (log-transformed) median coverage across all samples. We used the cubic function to calculate recurrent bias across the genome based on the median coverage. The recurrent coverage bias was subtracted from the (log-transformed) coverage in the original sample, which was then converted to normalized coverage by exponentiation.

#### Statistical phasing of parental haplotypes

We counted reference and alternate allelic depths in each cell using the ASEReadCounter module from GATK4 (version 4.0.1.2) at common SNP sites identified in 1000 Genomes Project Phase 3 reference haplotype panel (lifted to GRCh38). We selected variants with the minor allele observed in ≥ 5 cells as heterozygous variants for which statistical phasing was performed with EAGLE2 (version 2.4.1) using the 1000-Genome Phase 3 reference haplotype panel. The 3p-terminal region (0-33.7 Mb) was exceptional as most cells (including the 100-cell sample) showed loss-of-heterozygosity. To identify samples that were (partially) heterozygous, we first estimated the heterozygosity of each sample in this region using the fraction of minor allelic coverage at common variant sites with both genotypes observed in at least one sample. This led us to identify four cells (A2, A3, E1, and E7) with estimated heterozygosity > 0.05. We combined these four cells with the 100-cell sample (H1) and selected common variant sites with the minor genotype seen in at least two out of five samples as heterozygous variants. The parental haplotype phase at these variant sites were then derived from the major and minor genotypes from all samples instead of statistical phasing.

ASEReadCounter command:


gatk ASEReadCount -R <hg38_ref.fa> \-I <reads.bam > - O <allelic_depths.txt > -V <Variant_VCF>


with the same read filters for running HaplotypeCaller in bulk samples as described before.

EAGLE2 command:


eagle --vcfTarget = <filtered_hets.vcf.gz> \--vcfRef = <1000G_hg38_genotypes.bcf> \--geneticMapFile = <hg38_genetic_map.txt.gz> \--chrom=chr? \--outPrefix = ”phased_hets.vcf.gz” \--numThreads=12


#### Two-pass haplotype correction and allelic copy-number calculation

Compared to bulk copy number data, single-cell copy-number data have more variability but also display more significant allelic-depth differences in regions of allelic imbalance due to having only integer copy-number changes. To attenuate coverage variability due to amplification, we calculated total DNA copy number in 1 Mb intervals and allelic fractions in 50 kb intervals. The choice of 50 kb instead of 1 Mb intervals for allelic fraction calculation was because switching errors in statistical phasing occurs about once per 250 kb and will attenuate allelic depth difference in 1 Mb intervals in regions of allelic imbalance.

We performed allelic-depth-based haplotype correction in two passes. In the first pass, we aggregated allelic-depth differences in all single cells to detect recurrent allelic imbalance and correct switching errors in these regions. After the first pass, we reviewed the copy-number data and identified aneuploid samples with large segmental allelic imbalance. These samples were used for the second round of haplotype correction. The haplotype solution after the second pass was then used to calculate the final haplotype-specific DNA copy number. Details of this calculation can be found in the section Haplotype refinement using allelic imbalance in single-cell data in the Supplementary Information.

#### Determination of chromosomal copy number

To determine the integer copy-number state of each chromosome in a single-cell genome, we first normalized the average copy number of each chromosome by the median arm-level allelic copy number of both homologs across the genome. For near diploid genomes, the median allelic copy number is 1 and all copy-number states should be integers (0,1,2, …). As the minimum non-zero copy-number state is one, the presence of half integer copy-number states indicates duplication of the remaining chromosomes, i.e., whole-genome duplication; in this scenario, we multiplied the copy number by two to account for whole-genome duplication. We considered a genome to be near tetraploid if there was at least one chromosome arm with median allelic copy number between 0.2 and 0.8 and standard deviation of allelic coverage <0.25.

#### Joint mutation detection in single-cell samples

We performed joint somatic mutation detection in single cells using the same command line as described above for joint variant calling in bulk samples, with the only difference being the genome reference (GRCh38 instead of GRCh37). Variants were annotated using snpEff (http://pcingola.github.io/SnpEff) with the following command line argument: -v GRCh38.86 -ud 1000 -onlyProtein -canon.

### Longitudinal BE sequencing analysis

#### Data processing

With approval from the International Cancer Genome Consortium, we downloaded and re-aligned sequencing data from a previous study^64^ that were available from European Genome-phenome Archive (Dataset ID: EGAD00001006033) with controlled access. The cohort consisted of 773 BE/EAC (602 NDBE/IND, 109 LGD, 37 HGD, and 25 IM/EAC) samples from 88 patients. We performed normalization of 25 kb read-depth coverage in all samples using the coverage in 42 diploid NDBE samples from non-progressors (one sample from each individual) as a reference panel. Ten eigensamples generated from the reference panel by singular value decomposition were used for read-depth denoising of all samples.

#### Identification and classification of SCNAs

The low sequencing coverage does not allow haplotype-specific copy-number calculation. We manually reviewed the copy-number data and found that many samples contained a low fraction of aneuploid cells (see NDBE sample in Fig. 8C and examples in Supplementary Figs. 10 and 11). We expected that such events will likely be missed by standard copy-number segmentation algorithms and therefore manually reviewed the copy-number plot of each chromosome to identify the following SCNAs: (1) arm-level or whole-chromosome gain/loss, assessed from the genome-wide copy-number plots; (2) large segmental SCNAs (>1 Mb) that are shared by more than one sample; (3) recurrent focal deletions on 3p (near 60 Mb, spanning *FHIT*) and 9p (near 21 Mb, spanning *CDKN2A*); (4) complex SCNAs (including duplications/amplifications); (5) sloping copy-number variation. For complex SCNAs and sloping copy-number variation, we required at least part of the chromosome and most of the genome to have constant copy number to exclude false SCNA due to sequence coverage non-uniformity. We annotated SCNAs in each sample based on the evolution pattern (Supplementary Data 8, Table 1) and then generated a summary of SCNAs identified in all samples from each patient (Supplementary Data 8, Table 2); the latter was used to generate Supplementary Fig. 10.

### Reporting summary

Further information on research design is available in the Nature Portfolio Reporting Summary linked to this article.

### Supplementary information

Supplementary Information

Peer Review File

Description of Additional Supplementary Files

Supplementary Data 1-10

Reporting Summary

## Data Availability

Raw whole-genome sequencing data generated in the current study have been deposited into the database of Genotypes and Phenotypes (dbGaP) with accession code phs002706. Sequencing data are released with controlled access according to the approved IRB Protocols and the study protocol of the sequencing experiment. Data management, including approval for data access and reuse, and duration of data availability, is managed by dbGaP. Longitudinal BE sequencing data were obtained from the European Genome-phenome Archive (EGA) under accession code EGAD00001006033 through a data access agreement approved by the International Cancer Genome Consortium. The following data/results have been also uploaded to Zenodo [https://zenodo.org/record/8265676] and are publicly available: For sequencing data of BE/EAC samples in the current cohort: (1) intermediate and final haplotype-specific DNA copy number data and plots (grouped by patient and shown for each chromosome); (2) structural rearrangements; (3) somatic short sequence variants (single-nucleotide substitutions and insertion/deletions); and (4) DNA copy-number of single cells from a HGD lesion. For the longitudinal sequencing data, we only provided unphased DNA copy number plots of each chromosome in each sample from each patient.

## Code availability

All scripts and codes being used to generate the analytical results have been deposited to the same repository and are available at https://github.com/chunyangbao/NC_ESAD75.
