# Supplementary material for: Genomic signatures of past and present chromosomal instability in Barrett’s esophagus and early esophageal adenocarcinoma
Source: Nat Commun. 2023 Oct 4;14:6203. doi: 10.1038/s41467-023-41805-6 (PMC10550953; doi:10.1038/s41467-023-41805-6)
Supplement: Supplementary file 1 — Supplementary Information [file 41467_2023_41805_MOESM1_ESM.pdf]

Supplementary Information for

Genomic signatures of past and present chromosomal instability in Barrett's esophagus and early esophageal adenocarcinoma

Chunyang Bao and Cheng-Zhong Zhang

Corresponding Author: Cheng-Zhong Zhang
E-mail: cheng-zhong_zhang@dfci.harvard.edu

This PDF file includes:

Supplementary text
Figs. S1 to S22
Captions for Dataset S1 to S10
References for SI reference citations

Other supplementary materials for this manuscript include the following:

Datasets S1 to S10

Supplementary Notes

1. Analysis of somatic genome evolution in paired cancer and precancer samples

A. General goals of the analysis. The genomic analysis of paired BE and esophageal cancer lesions, or more generally, of multi-regional or longitudinal cancer samples, needs to accomplish three goals. First, we need to identify somatic alterations in these lesions. Second, we want to determine the phylogenetic relationship between these lesions (or different clones in these lesions) based on genetic alterations that are either shared or related between these lesions. (The difference between "shared" and "related" alterations will be discussed later). Finally, based on the phylogenetic relationship, we want to infer the evolutionary sequence of genetic changes, including their timing relative to disease progression. Below we discuss the bioinformatic and computational challenges related to each goal and strategies to overcome these challenges. We use examples from data in the current study to illustrate these challenges.

B. Technical challenges in the detection of genetic variants in FFPE DNA. The detection of genetic variants in FFPE DNA libraries is confounded by sequence artifacts generated during DNA extraction and library construction due to DNA degradation. These technical artifacts include: (1) base substitution errors introduced during the amplification of damaged DNA (including abasic or damaged bases) [1], which introduce false single-nucleotide (SNV) or insertion/deletion (INDEL) variants; (2) chimeric DNA (often consisting of sequences from opposite strands) generated by template-switching events during polymerase extension from nicked or single-stranded DNA templates [2], which may be falsely identified as sequence rearrangements; (3) uneven sequence coverage due to varying DNA recovery (degradation, chromatinization etc.), which can be falsely identified as copy-number alterations. Uneven sequence coverage also contributes to false negative detection of local sequence alterations including single-nucleotide variants, insertions/deletions, and rearrangements.

Besides sequencing artifacts, the limited amount of input DNA from micro-dissected tissue samples also results in low DNA library complexity that compromises variant detection sensitivity. Although the technical limitations of FFPE libraries can be ameliorated by modified experimental protocols of DNA fixation or extraction, they cannot be eliminated and will pose challenges to both variant detection and downstream analysis.

C. Analytical strategies to cope with technical limitations of FFPE sequencing. For sequence alterations including single-nucleotide variants (SNV), insertion/deletion (INDEL) events, and rearrangements, the specificity of their detection can be improved either by setting a more stringent threshold of variant read support or by using independent replicates (i.e., separate tissue specimens from the same tumor): The requirement for more variant read support may remove artificial sequence changes that are generated only on a small fraction of input DNA molecules; however, this requirement also leads to lower detection sensitivity due to uneven sequence coverage or low clonal fractions of tumor DNA.

In contrast to requiring more variant read support or increasing the mean sequencing depth, using biological replicates is a more efficient strategy for controlling sequence artifacts in our sample cohort as we are most interested in alterations that are present in more than one BE or EAC lesions and therefore informative about their phylogenetic relationship. We have therefore performed joint detection of both local variants (SNVs and INDELs) and rearrangements from all BE/EAC lesions from each patient instead of the standard tumor/normal pair analysis. (See **Joint variant detection by HaplotypeCaller** and **Somatic rearrangement detection** sections in **Methods** for more details.)

Although the joint variant detection strategy can suppress false positive variants in individual samples and achieve better sensitivity for shared variants, it has two limitations. First, it cannot distinguish between false variants and true private mutations that are only present in a single sample. It is therefore impossible to perform comprehensive genetic variant discovery in individual BE or EAC lesions, or benchmark detection accuracy. Second, joint variant detection cannot eliminate false negative variant detection (including genotyping of true variants) in individual BE/EAC lesions as the detection sensitivity depends on the quality of input DNA, the depth of sequencing, as well as the clonal fraction of tumor DNA.

As will be discussed in the next two subsections, the inadequate detection of SNVs, INDELs, and rearrangements can confound tumor phylogenetic inference based on these variants. By contrast, one can achieve more robust phylogenetic inference based on somatic copy-number alterations (SCNA) due to the better detection accuracy and the ability to phase SCNAs to parental chromosomes.

D. Computational and bioinformatic challenges related to tumor phylogenetic inference. The general rationale for tumor phylogenetic inference is that tumor clones with a more recent common ancestor should share more genetic alterations than tumor clones with an earlier ancestor. Therefore, the phylogenetic distance ("relatedness") between two tumor genomes is inversely correlated with the number of shared genetic alterations. A simple and commonly used approach is to estimate phylogenetic distance based on the number of genetic variants, including SNVs/INDELs, rearrangements, and SCNAs, that are shared between two tumor samples. However, this approach has several limitations.

First, because early/truncal mutations may be lost in progeny clones due to downstream **DNA deletions or loss-of-heterozygosity alterations**, the number of genetic variants shared between two tumor clones is not equivalent to the number of genetic alterations in their common ancestor. Consider an example of three tumor clones **A**, **B**, and **C**: **A** descended from the common ancestor, whereas **B** and **C** are expanded from sibling cells with reciprocal DNA gain and loss (such as shown in Fig. 6C and Fig. 7B). Although **B** and **C** share a more recent ancestor than **A**, there are actually more mutations shared between **A** and **B** or between **A** and **C** than between **B** and **C**, because **B** and **C** each have lost mutually exclusive subsets of ancestral mutations on the broken chromosome. This example offers a possible explanation for the discordance between phylogenetic trees determined from point mutations and from copy-number changes (see [3] for an example).

Note: We use this example only to illustrate the ambiguity of shared genetic variants as a measure of genetic relatedness when used for clustering-based phylogenetic inference. In principle, one can still use shared genetic variants to rank different phylogenetic trees (e.g., using a maximum likelihood approach) as long as variants in deleted regions are excluded from the calculation. However, the latter requires phasing of somatic variants that is usually not possible with shotgun sequencing data.

The second limitation of using the number of shared variants for phylogenetic relatedness is due to the presence of different subclones in a single tumor sample. Mutations detected in a polyclonal tumor sample may include mutually exclusive variants in different subclones. Therefore, the number of mutations shared between two tumor samples may not correspond to the number of mutations shared between specific tumor clones in each sample. Consider the following example: tumor sample 1 consists of 40% of tumor clone **A**, 10% of tumor clone **B**, and 50% stroma, and tumor sample 2 consists of 30% of tumor clone **B**, 5% of tumor clone **C**, and 65% stroma. The number of mutations shared between the two samples has no clear relationship to the genetic similarity between the tumor clones. To overcome this ambiguity, one should only consider mutations in the dominant subclone in each sample, i.e., **A** in tumor sample 1 and **B** in tumor sample 2. However, such analysis requires accurate mutant allele fraction calculation and subclonal inference that cannot be achieved with standard (30×) whole-genome sequencing.

Besides these two challenges, tumor phylogenetic inference is further confounded by false negative variant detection. Consider three tumor clones **A**, **B**, and **C** with 10,000 mutations in their common ancestor and an additional 1,000 mutations in the common ancestor of **B** and **C** (i.e., **B** and **C** diverged more recently from each other than from **A**). Assuming a false negative detection rate of 10%, we expect to detect $10,000 \times (1 - 0.1) = 9,000$ ancestral mutations in each clone, $10,000 \times 0.9^2 = 8,100$ mutations shared by two clones, and $10,000 \times 0.9^3 = 7,290$ mutations shared by all three clones. For the additional 1,000 mutations shared between **B** and **C**, we expect to detect $1,000 \times 0.9^2 = 810$ mutations in both. Adding these together, we expect to observe 7,290 truncal mutations in all three clones (not informative about their phylogeny), 810 ancestral mutations shared between **A** and **B** or between **A** and **C**, and 810 + 810 = 1620 mutations shared between **B** and **C**. Although these numbers still suggest a more recent ancestor between **B** and **C**, the additional 1,000 mutations in their ancestor is diluted by ancestral mutations that are missed in **B** or **C**, which appear as mutations shared by either **B** or **C** with **A**. If sample **C** has a lower detection sensitivity of 0.5 (e.g., due to low tumor cell fraction), then the observed number of mutations shared by **B** and **C** is approximately

$$\underbrace{11,000 \times 0.9 \times 0.5}_{\text{detected mutations shared by } \mathbf{B} \text{ and } \mathbf{C}} - \underbrace{10,000 \times 0.9^2 \times 0.5}_{\text{detected mutations shared by } \mathbf{A}, \mathbf{B}, \text{ and } \mathbf{C}} = 900.$$

This number is comparable to the number of detected mutations shared by A and B (= 810). This calculation highlights how false negative detection of ancestral mutations can confound the detection of shared mutations detected in related tumor clones.

As tumor samples (including many samples in our cohort) often contain less than 50% tumor cells (causing false negative detection of ancestral mutations) but frequently consist of more than one subclones (causing false assignment of mutations to tumor clones), we expect significant uncertainty in the phylogenetic distance estimated from the number of shared point mutations. This uncertainty is further aggravated by the prevalence of DNA losses in aneuploid BE and EAC genomes (causing irreversible loss of ancestral mutations), the presence of whole-genome duplication (further decreasing the detection sensitivity

of post WGD mutations), the high frequency of base substitution errors (causing false positive variant detection), and sequence coverage non-uniformity due to FFPE DNA degradation (causing false negative variant detection). Because of these limitations, we seek an alternative strategy of phylogenetic inference based on somatic copy-number alterations.

E. Advantages of SCNA-based phylogenetic inference. The first advantage of SCNA-based phylogenetic inference is their better detection accuracy from FFPE sequencing data than local sequence variants (SNVs, INDELs, and rearrangements). As discussed previously, both the non-uniformity of sequence coverage and the limited sequencing depth of FFPE DNA result in lower detection sensitivity of local sequence variants. As SCNAs affect large segments (including whole-chromosome arms) and alter both the total read depth and the allelic ratio between parental chromosomes, SCNA detection does not require deep sequencing and is insensitive to sequence dropout. Moreover, as individual SCNAs usually affect one of two parental chromosomes (homologs), they can be distinguished from sequence coverage non-uniformity due to technical artifacts (e.g., FFPE DNA degradation) that affect both homologs equally. By contrast, there is no strategy to "correct" false SNVs, INDELs, or rearrangements due to sequence artifacts in FFPE libraries (causing false positive detection).

The ability to detect SCNAs based on allelic coverage not only ensures the accuracy of SCNA detection but further enables phasing of SCNAs to parental chromosomes. By contrast, the haplotype phase of SNVs or rearrangements is generally not available from shotgun sequencing. The phasing of SCNAs to parental chromosomes enables the identification of ancestral SCNAs that are lost due to downstream DNA deletions when the ancestral SCNA and the downstream deletion are phased to the same homolog. This is not feasible for SNVs or rearrangements as one cannot be certain whether these variants were present on the deleted homolog or the intact homolog.

Finally, the phasing information of SCNAs enables us to draw evolutionary inferences about SCNAs or SCNA breakpoints in related BE/EAC samples. When SCNAs or SCNA breakpoints are phased to different parental chromosomes, they arise from independent alterations regardless of the distance between SCNA breakpoints. By contrast, when SCNAs/breakpoints are phased to the same parental chromosome, they could have descended from a single ancestral unstable chromosome with divergent secondary events. This capability enables us to identify mechanistically related chromosomal breakpoints, including reciprocal DNA retention/loss (Fig. 6C and Fig. 7B), progressive deletions (Fig. 6D), and other divergent outcomes of breakage-fusion-bridge cycles (Fig. 7A and 7C).

We note that whole-chromosome or arm-level SCNAs are less informative than segmental SCNAs. This is because segmental SCNAs are non-reiterative (i.e., arising only once) but gain or loss of a chromosome or chromosome arm can arise multiple times independently. Therefore, SCNA-based phylogenetic inference is best suited for tumors with high segmental SCNA burdens. However, this strategy does not require a large number of segmental SCNAs. This is because SCNA breakpoints are relatively rare (10-100 in BE/EAC clones) and shared SCNA breakpoints impose a strong constraint on the phylogenetic relationship between different tumor clones. The specificity of SCNA breakpoints as lineage markers contrasts with the ambiguity of SNVs due to DNA losses or false negative detection. Using haplotype-specific breakpoints for tumor phylogenetic inference is conceptually similar to using rearrangement breakpoints to determine the phylogeny of animal genomes [4]. A key difference between the two is that the analysis of organismal genome evolution only needs to consider breakpoints in a haploid genome ('gametes'), whereas the analysis of somatic genome evolution needs to consider breakpoints on each parental chromosome separately (i.e., haplotype-specific breakpoints) as the two homologs evolve independently.

2. Haplotype-specific DNA copy-number calculation

A. Calculation of haplotype fraction and allelic sequence coverage. To calculate phased average allelic fractions, the allelic depths of heterozygous genotypes (Suppl. Fig.S12 ii) were first converted to haplotype-specific (A or B) allelic depths based on the phase of genotypes (A or B). Based on the allelic fractions $F_{\mathrm{A}}(x)$ and $F_{\mathrm{B}}(x)$, and the normalized sequence coverage $R(x)$ (described in ***Standard copy-number analysis and estimation of sample purity and ploidy*** in **Methods**), we then calculated local allelic sequence coverage as $A(x) = R(x)F_{\mathrm{A}}(x)$ and $B(x) = R(x)F_{\mathrm{B}}(x)$. (Note that the total sequence depth $R(x)$ is centered at 2 such that $A(x)$ and $B(x)$ center at 1 in a diploid genome.) These data are plotted in "`xxx_phased.pdf`" files (available from the Online Data Repository) and used for downstream copy-number calculation.

The combination of normalized total sequence coverage $R(x)$ and phased average allelic fractions $F_{\mathrm{A,B}}(x)$ has two advantages. First, as the total sequence coverage $R(x)$ is calculated using both phased reads (i.e., those overlapping with heterozygous sites) and unphased reads, it shows less variability than the combined allelic depths at heterozygous sites. Second, the phased

average of allelic fractions at multiple adjacent variant sites reduces fluctuations of allelic fractions at single variant sites (Suppl. Fig.S12).

To see how the phased average improves the accuracy of allelic fraction calculation, we consider the distribution of allelic depths from D copies of genomic DNA from both homologs (i.e., mean sequencing depth = D) with allelic fraction f and $1 - f$. The mean allelic depths are given by Df and $D(1 - f)$. Assume the allelic depths follow a binomial distribution, the variance of allelic depths at a single variant site is $Df(1 - f)$ and the variance of allelic fraction at a single variant site is $f(1 - f)/D$. As the fluctuations of allelic fractions at different variant sites are largely independent, the variance of the average allelic fraction over n variant sites is approximately

$$f(1-f)\sum_i \frac{1}{D_i} = \frac{f(1-f)}{n}\left\langle \frac{1}{D} \right\rangle \sim \frac{1}{n}$$

where D_i is the total allelic depth at variant site i. Therefore, the statistical error of the average allelic fraction over n variant sites is reduced by $1/\sqrt{n}$ relative to the statistical error of allelic fractions at single variant sites. Importantly, the average allelic fractions need to be calculated based on the parental haplotype but not as the mean of major or minor allelic fraction as the latter are not equal to the allelic fraction of parental chromosomes. For example, let $F(x)$ denote the observed allelic fraction of haplotype A, then the major allelic fraction is given by

$$\max\left[F(x), 1 - F(x)\right] = |F(x) - 1/2| + 1/2.$$

It is straightforward to see that its expectation

$$E\left[|F(x) - 1/2| + 1/2\right] > E[F(x)] = f.$$

Therefore, a simple averaging of major or minor allelic fractions over consecutive variant sites will over-estimate allelic imbalance.

Although the phased average allelic fraction displays less variation than the allelic fraction at single variant sites, the averaging will reduce the resolution of allelic fraction changepoints and more importantly, reduce the signal of allelic imbalance when there are switching errors in the haplotype phase[5]. The rate of switching errors in statistical phasing was estimated to be 0.1-1%[6], or one switching error every 0.1-1Mb. To avoid inaccurate allelic fraction calculation due to haplotype switching, we performed phased average of allelic fractions in 25kb intervals with five or more variants. In our cohort, the lowest sequence depth is ≈ 12, and each sample has at least 1 million variants with sequence coverage (8 per 25kb). Based on these numbers, the average statistical error of phased allelic fractions in 25kb intervals is lower than

$$\sqrt{\frac{f(1-f)}{n}\left\langle \frac{1}{D} \right\rangle} \approx \sqrt{\frac{0.5^2}{5}\cdot\frac{1}{12}} = 0.06.$$

We further note that (1) the 25kb window sets the resolution of copy-number changepoints; (2) although one can theoretically detect SCNAs down to 25kb, the real sensitivity will depend on both the clonality and the size of SCNAs; (3) for deeper sequencing with both higher depth (D) and less dropout, the interval size (25kb) can be further reduced (e.g., to 10kb with 5 variants) to increase the resolution of SCNA changepoints and the detection of smaller SCNAs.

B. Validation of purity/ploidy estimates using phased allelic sequence coverage. Let $c_{\mathrm{A}}(x)$ and $c_{\mathrm{B}}(x)$ denote the (integer) DNA copy number of parental chromosomes, α denote the clonal fraction of tumor cells and τ denote the average ploidy of the tumor genome, we have [7]:

$$R(x) = 2 \times \underbrace{\frac{\alpha\left(c_{\mathrm{A}}(x) + c_{\mathrm{B}}(x)\right) + 2(1-\alpha)}{\tau\alpha + 2(1-\alpha)}}_{\text{local read depth ratio}}$$

and

$$F_{\mathrm{A}}(x) = \frac{\alpha c_{\mathrm{A}}(x) + (1-\alpha)}{\alpha\left(c_{\mathrm{A}}(x) + c_{\mathrm{B}}(x)\right) + 2(1-\alpha)}, \quad F_{\mathrm{B}}(x) = \frac{\alpha c_{\mathrm{B}}(x) + (1-\alpha)}{\alpha\left(c_{\mathrm{A}}(x) + c_{\mathrm{B}}(x)\right) + 2(1-\alpha)},$$

where the factor of 2 in R(x) is introduced such that the allelic depths

$$A(x) = R(x)F_A(x) = \frac{\alpha c_A(x) + (1-\alpha)}{\alpha \cdot \tau/2 + (1-\alpha)}, \quad B(x) = R(x)F_B(x) = \frac{\alpha c_B(x) + (1-\alpha)}{\alpha \cdot \tau/2 + (1-\alpha)} \qquad \text{[S1]}$$

center at 1 in a diploid genome.

As the tumor clonal fraction α and ploidy τ are both constant and $c_A(x)$ and $c_B(x)$ both take integer values, the allelic sequence coverage $A(x)$ and $B(x)$ should both cluster around discrete values (Suppl. Fig. S15) starting at $(1-\alpha)/[\tau\alpha + 2(1-\alpha)]$ corresponding to allelic copy-number state 0, with increments of $\alpha/[\tau\alpha + 2(1-\alpha)]$ reflecting a single copy gain. The clustering pattern of allelic sequence coverage can therefore be used to verify estimates of tumor clonal fraction and ploidy. We generated scattered plots of $A(x)$ and $B(x)$ of 25kb intervals for every chromosome in each sample in "`xxx_CNstates.pdf`" that is available in the Online Data Repository. We used the allelic coverage data to validate ploidy and purity estimates calculated by ABSOLUTE.

We note that the above derivations are for the simple case with one tumor clone with fraction α and average ploidy τ. In reality, many tumor specimens are polyclonal, possibly with opposite copy-number changes (i.e., segmental or arm-level loss in one clone, gain in a different clone) occurring to the same region. The inferences about the clonality or the integer copy-number states of SCNAs in polyclonal tumors are of less certainty. However, segmental SCNA changepoints, which are expected to be non-recurrent during genome evolution, can still be accurately detected (and their clonal fractions can be estimated from the change in the allelic depth); therefore, the strategy to use SCNA changepoints for phylogenetic inference is still feasible for polyclonal tumors even though the integer segmental copy-number in individual clones cannot be reliably determined.

C. Haplotype refinement and phasing of somatic copy-number alterations using allelic imbalance. In regions with allelic imbalance, the high and low allelic coverage should reflect the DNA copy number of parental chromosomes[7]. The oscillation of allelic coverage in these regions (Suppl. Fig. S15) is caused by switching between parental haplotypes from statistical phasing. To distinguish between allelic coverage oscillation due to haplotype switching and allele-specific SCNAs, we have implemented a computational workflow to correct switching errors and phase SCNAs based on the allelic coverage (Suppl. Fig. S14). The workflow consisted of four steps.

I. We first identified regions of allelic balance (5Mb) to estimate random fluctuations of allelic fractions. Deviation from allelic balance (allelic imbalance) can be assessed either from the mean allelic fraction (≈ 0.5) or from the standard deviation of phased allelic fractions. For FFPE data with significant coverage non-uniformity, the allelic fractions display significant variation even in disomic regions; we found the standard deviation of phased allelic fractions to be a better classifier of regions of allelic balance. As the primary goal of this step is to derive a reference distribution of allelic fractions in regions of allelic balance, we set a stringent cutoff (≤ 0.07 for most samples) that serves to exclude large regions of allelic imbalance but not necessarily include all regions of allelic balance. From the regions inferred to be in allelic balance, we accumulate 25kb level allelic fractions in these regions to derive a reference distribution of 25kb-level allelic fractions.

II. We next identified regions of allelic imbalance based on the reference allelic fraction distribution determined from regions of allelic balance. Due to uneven sequence coverage and the modest sequencing depths (~10x) and low tumor clonal fractions (< 0.4) in some of the samples, the allelic fractions in regions of allelic balance can display large fluctuations from the mean allelic fraction of 1/2. To discern true allelic imbalance from random fluctuations, we considered allelic fractions in multiple adjacent 25kb intervals. For m consecutive intervals, let $p_i (1 \leq i \leq m)$ denote the likelihood of allelic balance in each interval (two-sided p-value of the observed allelic fraction relative to the reference distribution), we can approximate the log-likelihood of allelic balance for the observed allelic fractions in m intervals as

$$L_0 = \sum_{1 \leq i \leq m} \ln p_i.$$

For true allelic imbalance due to gain or loss of DNA from one parental chromosome, we expect the phased allelic fractions to be either lower or higher than 1/2, i.e., with a unidirectional change; by contrast, random fluctuations should be bidirectional. This difference can be quantified from the frequency of allelic fraction transitions from above to below 1/2 or vice versa. Let δ be the probability that the allelic fraction transitions across 1/2 between adjacent intervals. Assume no correlation between the

allelic fractions in adjacent intervals, we have

$$\delta = \min\left[P(\mathrm{AF} > 0.5), P(\mathrm{AF} < 0.5)\right],$$

where $P(\mathrm{AF} < 0.5)$ and $P(\mathrm{AF} > 0.5)$ denote the cumulative probability that the allelic fractions are either below or above 0.5. In regions of allelic balance, we expect the allelic fraction to have the same probability to be either above or below 1/2, i.e., $\delta = 1/2$; in regions with allelic imbalance, we expect $\delta < 1/2$ as the mean allelic fraction is either above or below 1/2. For m intervals with a total of $m-1$ transitions, the log-likelihood of seeing s transitions is given by

$$\begin{aligned}&\ln\left[\frac{(m-1)!}{s!(m-1-s)!}\delta^s(1-\delta)^{m-1-s}\right]\\&=(m-1-s)\ln(1-\delta)+s\ln\delta-(m-1-s)\ln\left(1-\frac{s}{m-1}\right)-s\ln\frac{s}{m-1}.\end{aligned}$$

We can introduce a log-likelihood function for allelic imbalance as

$$L_1 = \underbrace{(m-s)\ln(1-\hat{\delta}) + s\ln\hat{\delta}}_{\text{allelic imbalance}} \quad - \quad \underbrace{m\ln 2}_{\text{allelic balance}} \quad .$$

The first term is from the log-likelihood of the observed number of transitions under the assumption of allelic imbalance ($\hat{\delta} < 1/2$) and the second (constant) term is from the log-likelihood of allelic balance ($\delta = 1/2$). The binomial coefficients cancel out from the subtraction. Combining the deviation (L_0) and the correlation (L_1) of allelic fractions, we can define a heuristic score of allelic imbalance based on allelic fractions in m consecutive intervals as

$$L = \sum_{1\le i\le m} \ln p_i - \left[(m-s)\ln(1-\hat{\delta}) + s\ln\hat{\delta}\right]. \qquad \text{[S2]}$$

We chose $m = 10$ such that there is on average less than one switching error across the adjacent intervals (250kb) from statistical phasing. We operationally set $\hat{\delta} = 0.1$. For each 25kb bin, we calculated the allelic imbalance score based on the allelic fractions in 10 adjacent bins. To determine the cutoff for calling allelic imbalance, we compared the distribution of allelic imbalance scores in regions of allelic balance against the distribution in selected regions of allelic imbalance. The cutoffs were set individually for each sample and chosen to be permissive in order to identify subclonal SCNAs in low-purity tumor samples.

III. We next performed allelic-depth phasing on each chromosome based on 25kb-level allelic coverage. We concatenated all 25kb bins that were determined to be in allelic imbalance and assessed potential haplotype switching between adjacent bins causing a swap of allelic depths. The general idea is illustrated in Suppl. Fig. S14 and examples are shown in Suppl. Fig. S15. For each interval i, we compared the allelic depths (A_i, B_i) in this interval with the average allelic depths (A, B) of the left 40 intervals (1Mb) with allelic imbalance and calculated

$$\underbrace{\min(|A_i - A|, |B_i - B|)}_{\text{no swap}} - \underbrace{\min(|A_i - B|, |B_i - A|)}_{\text{swap}}.$$

If the differential metric was ≤ 0.05, we kept the haplotype phase of this interval; if the differential was above 0.05, we switched the haplotype from the ith interval to the end of the chromosome. The same procedure was then performed from the last interval to the first by comparing allelic depths in each interval to the right 40 bins. Allelic-depth phasing was performed individually and the results are plotted in "`xxx_ADCorr.pdf`" in the Online Data Repository.

IV. Finally, we combined haplotype corrections from individual samples from each patient to derive the consensus parental haplotype. This step both improved the accuracy of local haplotype linkage by aggregating allelic depths from all samples with allelic imbalance and generated longer contiguous haplotypes by concatenating haplotypes determined from allelic imbalance in different samples. The latter was especially powerful given the prevalence of both arm-level and segmental allelic imbalance in BE/EAC genomes. Examples of joint haplotype inference from allelic imbalance in all samples are shown in Suppl. Fig S16. The final jointly inferred haplotype still contained occasional long-range switching errors (usually 1-2 switching errors on 1-3 chromosomes per individual). These errors were corrected manually. Readers can refer to codes provided in the repository (see

"Code Availability") for both the implementation of algorithms in each step of the calculation and the final manual curation. Using the jointly inferred haplotype, we re-calculated haplotype-specific DNA copy number in 25kb intervals. These results are plotted in "`xxx_final.pdf`" (available in the Online Data Repository).

We want to add a few comments about allelic-depth based phasing. First, this strategy is restricted to regions of allelic imbalance and is necessary because haplotype mixing will average out the difference between the allelic fractions of parental chromosomes, which reduces the signal of allelic imbalance. In regions of allelic balance, the allelic copy number is simply half of the total copy number and haplotype correction is unnecessary. Second, the single-sample allelic-depth phasing algorithm assumes *a priori* that multiple SCNAs on a chromosome are more likely to have accumulated on a single parental homolog than originate from both homologs (Suppl. Fig S14ii, middle). This assumption is based on the biological insight that it is more likely to accrue multiple SCNAs on a single unstable chromosome, either from catastrophic events such as chromothripsis or through multigenerational processes such as breakage-fusion-bridge cycles, than to acquire SCNAs on both homologs through independent DNA breaks and partially supported by the observed patterns of SCNAs in multifocal BE samples (Suppl. Fig. S3E). For segmental SCNAs interspersed with regions of allelic balance (Suppl. Fig. S15iii), this assumption cannot be directly validated. However, we were able to validate some of the long-range phasing of SCNAs either by rearrangements joining distal copy-number changepoints (Suppl. Fig. S8F) or using chromosome-scale haplotypes derived from whole-chromosome (Suppl. Fig. S16i) or large overlapping regions of allelic imbalance (Suppl. Fig. S16ii,iii). Finally, even when both parental chromosomes acquire SCNAs (Suppl. Fig. S15iv, S16ii,iii), sometimes even related to each other (Patient 7 Chr.12, Suppl. Fig. S8H), we were still able to separately phase SCNAs on each parental chromosome based on rearrangements and allelic copy-number changes at SCNA changepoints. The solution of these more complex cases required manual review.

D. Segmentation of haplotype copy number. For each BE/EAC sample, we first calculated the integer DNA copy number $c_{\mathrm{A}}(x)$ and $c_{\mathrm{B}}(x)$ of parental chromosomes in 25kb intervals from the haplotype-specific sequence coverage $A(x)$ and $B(x)$, the clonal fraction α, and the ploidy τ of the major BE/EAC clone using Eq. (S1). We then segmented $c_{\mathrm{A}}(x)$ and $c_{\mathrm{B}}(x)$ by an iterative procedure described previously for single-cell DNA data that only have integer copy-number states. Briefly, in each round of iteration, the 25kb copy number was re-calculated as the local median of 9 consecutive bins (0.23 Mb) and then rounded to the nearest integer; the iteration ended when the copy number of all bins converged to integer states. We then collected all copy-number changepoints and re-calculated the segmental copy-number states. The final segmented (using 25kb bins) and 100kb-average integer DNA copy number data are plotted in "`xxx_final_100kb.pdf`" (available in the Online Data Repository), with annotations of all SCNAs. Whole-genome duplication status was assigned if there were more parental chromosomes with duplicated copy number (CN=2) than single copy (CN=1). Because we constrained copy-number changes to integers, the minimum detectable SCNA was dependent on both the clonality and the copy-number difference across changepoints. For clonal single-copy changes (i.e., one copy loss or gain), the minimum CNA length was ~ 0.12Mb (five bins); for clonal two-copy changes (rare), the minimum CNA length was ~ 0.08Mb (3 bins). As this procedure converged to only integer copy-number states, it would incorrectly segment non-integer copy-number states due to incorrect purity/ploidy estimates or in regions of subclonal SCNAs. Non-integer subclonal copy-number states were easily identifiable and annotated in the final copy-number plots; subclonal SCNAs were not used for phylogenetic inference except where stated.

E. Haplotype refinement using allelic imbalance in single cell data. Due to the variability of sequence coverage in single-cell libraries, we used a different strategy to perform haplotype correction from allelic imbalance in all single cells. Let $c_{\mathrm{A},i}(x)$ and $c_{\mathrm{B},i}(x)$ be the local allelic copy number in cell i at genomic location x. For any two bins (x and y) on a single chromosome, we expect one homolog to have constant DNA copy number in most cells, i.e.,

$$c_{A,i}(x) = c_{A,i}(y) \text{ or } c_{B,i}(x) = c_{B,i}(y).$$

If there is haplotype switching between x and y, we expect

$$c_{A,i}(x) = c_{B,i}(y) \text{ or } c_{B,i}(x) = c_{A,i}(y).$$

To assess the likelihood of haplotype switching between x and y, we introduced

$$\delta(x,y) = \sum_i \mathbb{1}\left[\min\left(\left|c_{A,i}(x) - c_{A,i}(y)\right|, \left|c_{B,i}(x) - c_{B,i}(y)\right|\right)\right] > \delta$$

and

$$\delta'(x,y) = \sum_i \mathbb{1}\left[\min\left(\left|c_{A,i}(x) - c_{B,i}(y)\right|, \left|c_{B,i}(x) - c_{A,i}(y)\right|\right)\right] > \delta.$$

$\delta(x,y)$ represents the number of cells where the local allelic copy number states in bin x and bin y show a significant difference ($> \delta$) based on the current haplotype phase and $\delta'(x,y)$ represents the same number with the opposite haplotype linkage between bin x and bin y. In regions of allelic balance, even if there are occasional allelic copy-number differences above the threshold, we expect such differences to be random and cancel out among many cells. By contrast, in regions of allelic imbalance that is shared by multiple cells, the gained homolog or deleted homolog will be preserved, producing recurrent allelic bias that can be used for haplotype correction. We therefore used

$$\epsilon(x,y) = \delta'(x,y) - \delta(x,y)$$

as a penalty function for switching errors. $\epsilon(x,y) > 0$ favors the current haplotype linkage between x and y as there are fewer samples showing significant changes in both allelic copy-number states ($>\delta$), and vice versa. Let $s(x) = 1$ represent the haplotype phase of 50kb bins, we iteratively flip $s(x)$, i.e., $s(x) \to -s(x)$ to find the haplotype phase s(x) that minimizes

$$E = -\sum_{x,y} \epsilon(x,y)s(x)s(y). \quad \text{[S3]}$$

It is straightforward to verify that the minimization of E produces the correct haplotype in regions with at least one parental chromosome having constant DNA copy number. This assumption was verified as we usually observed at least homolog with constant copy number across the entire chromosome (Supplementary Data S3), even when there were multiple SCNAs on the other homolog.In the first pass of allelic-depth based phasing, we used local haplotype-specific copy number (50kb bins) in all cells with a cutoff penalty $\delta = 0.6$. In the second pass, we only used aneuploid cells with cutoff penalty $\delta = 0.8$.

3. Technical analysis of haplotype-specific copy-number calculation

The haplotype-specific coverage or allelic depth is calculated as the total sequence coverage $R(x)$ multiplied by the average allelic fraction of each parental homolog $F_A(x)$ and $F_B(x)$, where x is the genomic coordinate. For 10× mean sequencing coverage, the average number of reads/fragments in 10kb intervals is approximately

$$10 \times 10^4/\text{read length} = 300 - 500.$$

The standard deviation of read count is roughly proportional to the square root of the read count; therefore, the statistical error of normalized coverage $\delta R(x)/\overline{R(x)}$ is approximately $1/\sqrt{300} \approx 0.06$. This estimation implies that 10× mean sequencing depth is sufficient for the calculation of average sequence coverage $R(x)$ in 10kb or bigger intervals.

In above, we showed that the statistical error of the phased average allelic fraction is approximately

$$\sqrt{\frac{f(1-f)}{n}\left\langle\frac{1}{D}\right\rangle},$$

where f is the haplotype fraction, n is the number of phased variants, and $\langle 1/D\rangle$ is the mean of the inverse of sequence coverage at phased variant sites. Both n and D depends on the mean sequence coverage. We therefore first assessed the dependence of allelic depths on the sequencing depth.

A. Dependence on the sequencing depth. To determine the minimum sequencing depth required for the accurate calculation of allelic depths, we calculated allelic depths from down-sampled allelic coverage in several BE/EAC samples and compared the results calculated from down-sampled coverage with those derived from the original. To generate down-sampled allelic coverage,

we randomly selected reads at each variant site based on the down-sampling factor. The down-sampling was only applied to allelic coverage and therefore only affected the calculation of allelic fractions.

Results from this analysis demonstrate that single-copy DNA deletions with ≥50% clonality can be reliably detected at 10× or even 5× mean sequencing depth. An example of this analysis is presented in Fig. S18 and additional examples can be found in Pages 1-4 of Additional Data S9: DownSamplePlots.pdf. Below we will verify this result based on quantitative estimates of the allelic depth change and its statistical variation.

The statistical error of allelic depth can be estimated as

$$\delta\left(R(x)F(x)\right) \approx \overline{R(x)} \cdot \delta F(x) + \delta R(x) \cdot \overline{F(x)},$$

where $\overline{R(x)}$ and $\overline{F(x)}$ are the expected local sequence coverage and allelic fraction, and $\delta R(x)$ and $\delta F(x)$ are the statistical errors. At 10× mean sequencing depth, the statistical error of the phased average allelic fraction (restricted to intervals with five or more variants with coverage) is approximately

$$\delta F(x) \approx \sqrt{\frac{f(1-f)}{n}\left\langle\frac{1}{D}\right\rangle} \approx \sqrt{\frac{0.5 \cdot 0.5}{5} \cdot \frac{1}{10}} \approx 0.07.$$

The statistical error of the total sequence coverage is approximately

$$\frac{\delta R(x)}{\overline{R(x)}} \approx \frac{1}{\sqrt{300}} \approx 0.06$$

based on 300 unique fragments in 10kb intervals. Therefore, the combined statistical error of allelic depth is approximately

$$\overline{R(x)} \cdot 0.07 + \overline{R(x)} \cdot 0.06 \cdot \overline{F(x)}.$$

In disomic regions, $\overline{R(x)} \approx 2$ and $\overline{F(x)} \approx 1/2$, and the statistical error is

$$\delta\left(R(x)F(x)\right) \approx 0.1 \cdot \overline{R(x)} \approx 0.2.$$

Based on Eq. (S1), the allelic depths are given by

$$A(x) = \frac{\alpha c_{\mathrm{A}}(x) + (1-\alpha)}{\alpha\tau/2 + (1-\alpha)}, \quad B(x) = \frac{\alpha c_{\mathrm{B}}(x) + (1-\alpha)}{\alpha\tau/2 + (1-\alpha)}$$

where α is tumor purity, τ is tumor ploidy, $c_{\mathrm{A,B}}$ are the integer allelic copy-number states. For near diploid genomes, $\tau \approx 2$, a single copy change $\Delta c_{\mathrm{A,B}} = \pm 1$ will result in a change in the normalized allelic depth of

$$\delta = \frac{\alpha}{\alpha\tau/2 + (1-\alpha)} \approx \alpha.$$

For near tetraploid genomes, $\tau \approx 4$, a single copy change will result in a change in the normalized allelic depth of

$$\delta = \frac{\alpha}{2\alpha + (1-\alpha)} \approx \frac{\alpha}{1+\alpha}.$$

Therefore, if we require the allelic depth change δ to be at least twice the standard error, then the minimum clonality of detectable single-copy changes in a near diploid genome at 10× depth is $\alpha = 0.2 \times 2 = 0.4$ and the minimum clonality of detectable single-copy changes in a near tetraploid genome is $\alpha = 2/3 \approx 0.67$.

We note that these thresholds are derived based on the allelic depths in 25kb intervals with five or more variants. In our copy-number workflow, we considered multiple consecutive 25kb intervals to identify regions of allelic imbalance and further performed joint haplotype inference based on allelic imbalance in all related BE/EAC samples from the same patient. The joint haplotype inference from allelic imbalance in multiple samples enables long-range haplotype phasing. With long-range haplotype information, we can perform phased averaging of allelic fractions in larger intervals to improve the accuracy of allelic

depth calculation. In the final haplotype copy-number calculation, we chose 100kb intervals that have on average ~ 30 phased variants with coverage. This reduced the statistical error of allelic fraction to

$$\delta F(x) \approx \sqrt{\frac{f(1-f)}{n}\left\langle\frac{1}{D}\right\rangle} \approx \sqrt{\frac{0.5\cdot 0.5}{30}\cdot\frac{1}{5}} \approx 0.04$$

even at 5× mean sequencing depth. The statistical error of allelic depth is approximately

$$\delta\left(R(x)F(x)\right) \approx \overline{R(x)}\cdot(0.04+0.03) \approx 0.14$$

which is much lower than the change in allelic depth due to single-copy changes with clonality $\alpha = 40\%$ in either near diploid ($\delta \approx \alpha = 0.4$) or near tetraploid samples ($\delta \approx \alpha/(1+\alpha) = 0.29$).

In our cohort, samples from Patient 8 had the lowest sequencing depth (5 − 8×). Moreover, the three samples with the lowest depths: HGD1 (5.5×), HGD2 (6.3×), and IMEAC (7.2×) had high ploidy (HGD1:3.83; HGD2:2.83; IMEAC:2.9). The purity estimates for these clones were around 0.4. For the HGD1 sample with ploidy 3.83 and purity 0.39, the allelic depth difference due to single-copy difference is approximately

$$\delta = \frac{0.39}{3.83/2\cdot 0.39+(1-0.39)} \approx 0.28.$$

The above estimation establishes that this change is significantly higher than the statistical error of allelic depth calculated for 100kb intervals (~ 0.14).

B. Dependence on SCNA clonality. We next analyzed the dependence of allelic depth difference on the clonality of copy-number alterations. In this analysis, we calculated the allelic depths in simulated tumor/normal mixtures with 20× mean sequence coverage and projected tumor cell fractions ("tumor purity") in the range of 10-40%. We first calculated the fractional depths of tumor reads and normal reads based on the estimated tumor cell fraction of the original sample, the projected tumor cell fractions, and the final sequencing depth of 20×. We then calculated the total sequence coverage $R(x)$ as a linear mixture:

$$R(x) = \alpha R_{\text{tumor}}(x) + (1-\alpha)R_{\text{normal}}(x).$$

For the allelic coverage, we performed separate down-sampling on the tumor allelic coverage and the normal allelic coverage based on the fractional sequencing depths and combined the allelic coverage to represent the final allelic coverage.

Results from this analysis suggest that the minimum clonality of single-copy changes that is resolvable from 25kb-interval allelic coverage is 18-20%. An example of this analysis is presented in Fig. S19 and additional examples can be found in Pages 5-8 of Additional Data S9: DownSamplePlots.pdf.

We note that most samples in our collection have purity above 30% that is well above the detection limit (18%). The following samples have less than 30% purity: EAC from Patient 6 (27%), BE from Patient 9 (27%), HGD from Patient 13 (27%), BE (24%) and IND (16%) from Patient 14, and BE (28%), HGD1 (27%), HGD2 (27%), and IMEAC2 (24%) from Patient 15. Below we discuss each sample separately.

EAC from Patient 6: This sample had estimated tumor purity of 27% and ploidy of 3.38, and was sequenced to 25× mean coverage. The average number of phased variants in 25kb intervals is about 10. Based on these numbers, we estimate the statistical error of allelic fractions in disomic regions to be

$$\delta F(x) \approx \sqrt{\frac{f(1-f)}{n}\left\langle\frac{1}{D}\right\rangle} \approx \sqrt{\frac{0.5\cdot 0.5}{10}\cdot\frac{1}{25}} \approx 0.03.$$

The statistical error of allelic depths is ~ 0.03 × 2 = 0.06, which is significantly lower than the allelic depth difference expected for single-copy changes

$$\delta = \frac{0.27}{0.27\cdot 3.38/2+1-0.27} = 0.26.$$

BE from Patient 9: The only SCNA in this sample was 9p uniparental disomy (UPD). UPDs cause reciprocal single-copy gain and loss to both homologs, resulting in an allelic depth difference of

$$A(x) - B(x) = 2 \times \frac{\alpha}{\alpha\tau/2 + (1-\alpha)}.$$

In this sample, the estimated ploidy is 1.96 and the estimated purity is 27%. Therefore the allelic depth difference on 9p is

$$A(x) - B(x) = \frac{2 \cdot 0.27}{0.27 \cdot 1.96/2 + 1 - 0.27} \approx 0.54,$$

which is significantly higher than the statistical error (~ 0.14). Moreover, the resolution of Chr.9 haplotype in this patient based on allelic imbalance in HGD2, EAC1, and IMEAC2 samples ensured the accuracy of 9p allelic depth calculation in the BE sample (10× sequencing depth, near diploid).

HGD from Patient 13: This sample had estimated tumor purity of 27% and ploidy of 3.62, and was sequenced to 23× mean coverage. The statistical error of allelic depths and the allelic depth difference for single-copy changes are both similar to the EAC sample from Patient 6. The sequencing coverage is therefore sufficient for the detection of single-copy changes.

BE and IND from Patient 14: Both samples were near diploid and shared 9p UPD, whose detection sensitivity is higher because of the two-copy difference between the two homologs. The IND sample (21.7×) also contained a single-copy gain of Chr.8. The detection of Chr.8 gain in this sample was enabled by the determination of Chr.8 haplotype phase based on allelic imbalance in samples HGD1 and IMEAC from the same patient.

BE, HGD1, HGD2, and IMEAC2 from Patient 15: These samples were sequenced to at least 23.2× (in the HGD2 sample). The statistical error of allelic fractions is approximately $\delta F(x) \sim 0.03$. Because all the samples have near diploid genomes, the allelic depth difference due to single-copy changes is approximately equal to the purity (0.24-0.28). Therefore, the sequencing depth is sufficient to detect clonal single-copy changes (e.g., Chr.12q and Chr.17p). The biggest challenge with samples from this patient is the presence of many subclonal copy-number changes. This is overcome by joint haplotype inference based on allelic imbalance in all samples. These include completely resolved haplotypes on Chr.1 and Chr.2 (HGD1,HGD/IM), Chr.4 (HGD1, HGD/IM, IMEAC2), Chr.5 (HGD/IM, BE, IMEAC2, HGD2, EAC1), Chr.6 (BE, IMEAC2, HGD2, EAC1), Chr.7 and Chr.8 (IMEAC2, EAC1), Chr.9 (HGD1,HGD/IM, EAC1), Chr.10 (IMEAC2, EAC1), Chr.11p (BE, IMEAC2, HGD2), Chr.13q (HGD1, HGD/IM), Chr.15q (HGD2, EAC1), Chr.18 (HGD/IM, IMEAC2), Chr.19 (HGD/IM, BE, IMEAC2, HGD2), Chr.20 (HGD1, HGD/IM, IMEAC2), Chr.21q (HGD/IM, BE, IMEAC2, HGD2), Chr.22q (BE, IMEAC2, HGD2). The phylogeny of these samples was determined based on segmental copy-number alterations on Chr.2q and Chr.11p (see Fig. S17 and caption for more details).

In summary, the tumor/normal mixture benchmarking and the statistical estimation both demonstrate the capability to reliably detect single-copy changes with 20% or higher clonality at 20× sequencing depth through a combination of phased average allelic fraction and joint haplotype inference from multiple samples with allelic imbalance. *We note that in a single tumor sample, the minimum clonality of detectable SCNAs will depend on the uniformity of sequence coverage in the DNA library and may not reach 20% for FFPE libraries with significant variation in the sequence coverage.*

C. Validation of allelic-imbalance based haplotype inference. In the last subsection of this document, we provide validation of the method of haplotype inference based on allelic imbalance. For this validation, we used the whole-genome sequencing data of clones expanded from single post-crisis retina pigmental epithelium (RPE-1) cells [8] together with truth haplotype data of RPE-1 cells generated in a previous study from us [5]. The truth haplotype was determined by computational haplotype inference from linked-reads and Hi-C sequencing of RPE-1 cells and validated by the sequencing of monosomic RPE-1 cells.

We had previously used the RPE-1 haplotype data to calculate haplotype-specific DNA copy number of both parental chromosomes in these clones and resolved all segmental copy-number alterations. For this validation, we combined statistical haplotype phasing and allelic-imbalance based haplotype inference to calculate haplotype-specific DNA copy number. These results were then compared against results derived using the truth haplotype for validation.

The alignment and post-alignment processing of the sequencing reads were described in our previous paper [5]. Reads were aligned to the GRCh38 reference. We performed genotyping and statistical phasing using the newly generated reference haplotype data from the 1000 Genomes project (available from http://ftp.1000genomes.ebi.ac.uk/vol1/ftp/data_collections/

1000G_2504_high_coverage/working/20201028_3202_phased/). These data were generated by variant calling and phasing (with shapeit2) of sequencing reads that were aligned to GRCh38, which may have some differences from the liftover of reference haplotypes from GRCh37 to GRCh38. Haplotype phasing and haplotype-specific copy-number calculation were performed using the same algorithm as described in **Haplotype refinement and phasing of somatic copy-number alterations using allelic imbalance**. Results from this analysis are presented in Additional Dataset S10: AllelicDepthPhasing.pdf and selected examples are presented in Figs. S20-S22.

In comparison to the WGS data of BE/EAC samples, the RPE-1 WGS data had better quality as they were derived from live cells instead of degraded FFPE DNA. The RPE-1 libraries were also sequenced deeper than the BE/EAC samples (30-40× mean depth). Because of these advantages, the RPE-1 data had both better variant coverage and better uniformity of total sequence coverage. To make a fair comparison, we chose to perform haplotype inference using allelic depths calculated for 10kb intervals, which have similar allelic coverage as 25kb allelic depths in the BE/EAC data. Importantly, *many copy-number alterations in the RPE-1 data were inferred to be subclonal*. This enabled us to evaluate the capability to detect subclonal SCNAs and perform haplotype inference based on subclonal allelic imbalance.

In Additional Dataset S10, we first present genome-wide copy-number plots of all RPE-1 clones (Pages 1-2), with red and blue dots corresponding to the 1Mb-level DNA copy number of each parental haplotype. On Page 3, we show the results of haplotype phasing from copy-number gains of 10q and 12p that are shared by all RPE-1 clones. (The 10q example is included in Fig. S20.) These examples demonstrate that the phasing algorithm can accurately resolve the long-range haplotype phase in large regions of allelic imbalance that is indicated by the agreement between the allelic-imbalanced derived haplotype and the truth haplotype. In pages 4-20 of Additional Dataset S10, we show the results of haplotype phasing in all chromosomes with allelic imbalance (determined using the truth haplotype) in all post-crisis RPE-1 clones. For each clone, the first plot shows the genome-wide DNA copy number and each subsequent panel shows the results of haplotype phasing on a single chromosome with either arm-level or segmental allelic imbalance.

We manually reviewed all chromosomes with partial or complete allelic imbalance identified with the complete haplotype data (40 chromosomes total). The results are summarized below.

- X-29 (one instance): Chr.11 (no error)
- X-32 (one instance): Chr.18 (one switching error at the centromere)
- 24-141 (two instances): Chr.7 (no error), Chr.12 (no error, opposite haplotype assignment of iso-12p is unrelated to alterations to 12q; **sloping copy-number variation at the 12q-terminus**)
- 24-144 (two instances): Chr.3 (no error; minor subclonal SCNAs on 3p affect both homologs and are unresolvable), Chr.6 (unable to resolve subclonal gains on 6p with allelic depth difference ≈ 0.1, 6p-terminus was phased correctly).
- X-33 (four instances): Chr.1 (unable to resolve subclonal 1q loss with allelic depth difference ≈ 0.09), Chr.8 (one switching error on 8p, no error in complex segmental gains on 8q), Chr.18 (no error; **note the presence of subclonal 18q-terminal loss with very low clonal fractions**), Chr.X (no error).
- I-dox-1 (five instances): Chr.2 (two switching errors on 2q near the centromere), Chr.7 (one switching error on 7q), Chr.8 (one switching error on 8p), Chr.11 (no error), Chr.15 (one switching error on 15q).
- X-25 (five instances): Chr.4 (no error; **SCNAs on both homologs**), Chr.8 (no error), Chr.12 (no error), Chr.13 (no error; **partial retention near the 13q terminus with very low clonal fractions**), Chr.X (no error)
- X-37 (six instances): Chr.1 (one switching error of the 1q-terminal deletion), Chr.14 (no error), Chr.15 (no error; **sloping copy-number variation**), Chr.17 (one switching error on 17p), Chr.18 (no error; **sloping copy-number variation on 18q**), Chr.22 (unable to resolve subclonal 22q loss with allelic depth difference 0.09 (q-ter) − 0.13 (pericentric)).
- X-36 (six instances): Chr.2 (one switching error on 2p **with sloping copy-number variation**; 2q haplotype derived from subclonal loss with allelic depth difference ~ 0.16 had a few local errors), Chr.4 (one switching error at the centromere), Chr.6 (a few local errors at the 6q terminus with allelic depth difference ~ 0.20; **this region has sloping copy-number variation**), Chr.7 (no error), Chr.10 (no error), Chr.18 (one switching error at the centromere)

- X-35 (eight instances): Chr.1 (no error), Chr.5 (a few local errors on 5q:50-100Mb with allelic depth difference ≈ 0.16; **sloping copy-number variation**), Chr.6 (no error; **sloping copy-number variation near 6q-terminus**), Chr.8p (unable to resolve subclonal gain of 8p with allelic depth difference ≈ 0.08), Chr.10 (two switching errors on 10q near the centromere), Chr.12p (no error), Chr.16 (two switching errors on 16p), Chr.18 (one switching error at the centromere; **sloping copy-number variation on 18p and 18q**).

Except for four SCNAs with clonality <14% (24-144:Chr.6; X-33:Chr.1; X-37:Chr.22; X-35:Chr.8) that can only be resolved with the truth haplotype data, all the other SCNAs can be resolved using 10kb allelic depth data. For most chromosomes, the haplotype inferred by allelic imbalance has either no (20/40) or one long-range switching error (10/40) that is often at the centromere or near regions with low complexity or variant density. Three chromosomes had two switching errors. These switching errors are easily identifiable. Three copy-number alterations with clonality of 15-20% are resolvable but contain some local phasing errors (X-36:Chr.2q; X-36:Chr.6; X-35:Chr.5). In particular, our phasing algorithm can generate correct haplotype inference even in regions where both homologs are altered and in regions with complex segmental gains and losses (Fig. S21). Together, these results demonstrate the robustness of our algorithm for allelic-imbalance based haplotype inference.

We note that this analysis also provides *independent evidence about the copy-number outcomes of breakage-fusion-bridge cycles or telomere crisis* in addition to results from our prior work[9] as the copy-number alterations in post-crisis RPE-1 clones are initiated by telomere de-protection. In particular, we identified multiple examples of sloping copy number variation, two of which are shown in Fig. S22. These results demonstrate the robustness of our haplotype-inference algorithm even when one homolog has non-constant copy number.

References

1. Do, H. and A. Dobrovic, Sequence artifacts in DNA from formalin-fixed tissues: causes and strategies for minimization. Clin Chem, 2015. 61(1): p. 64-71.
2. Haile, S., et al., Sources of erroneous sequences and artifact chimeric reads in next generation sequencing of genomic DNA from formalin-fixed paraffin-embedded samples. Nucleic Acids Res, 2019. 47(2): p. e12.
3. Wang, Y., et al., Clonal evolution in breast cancer revealed by single nucleus genome sequencing. Nature, 2014. 512(7513): p. 155-60.
4. Ferguson-Smith, M.A. and V. Trifonov, Mammalian karyotype evolution. Nat Rev Genet, 2007. 8(12): p. 950-62.
5. Tourdot, R.T., Brunette, G.J., Pinto, R.A., and Zhang, C.-Z. Determination of complete chromosomal haplotypes by bulk DNA sequencing. Genome Biol 2021. 22(139).
6. Loh, P.R., et al., Reference-based phasing using the Haplotype Reference Consortium panel. Nat Genet, 2016. 48: p.1443-1448.
7. Carter, S.L., et al., Absolute quantification of somatic DNA alterations in human cancer. Nat Biotechnol, 2012. 30: p. 413-421.
8. Maciejowski, J., Li, Y., Bosco, N., Campbell, P.J., and de Lange, T, Chromothripsis and kataegis induced by telomere crisis. Cell, 2015. 163: p. 1641-1654.
9. Umbreit, N.T., et al., Mechanisms generating cancer genome complexity from a single cell division error. Science, 2020. 368(6488): eaba0712.

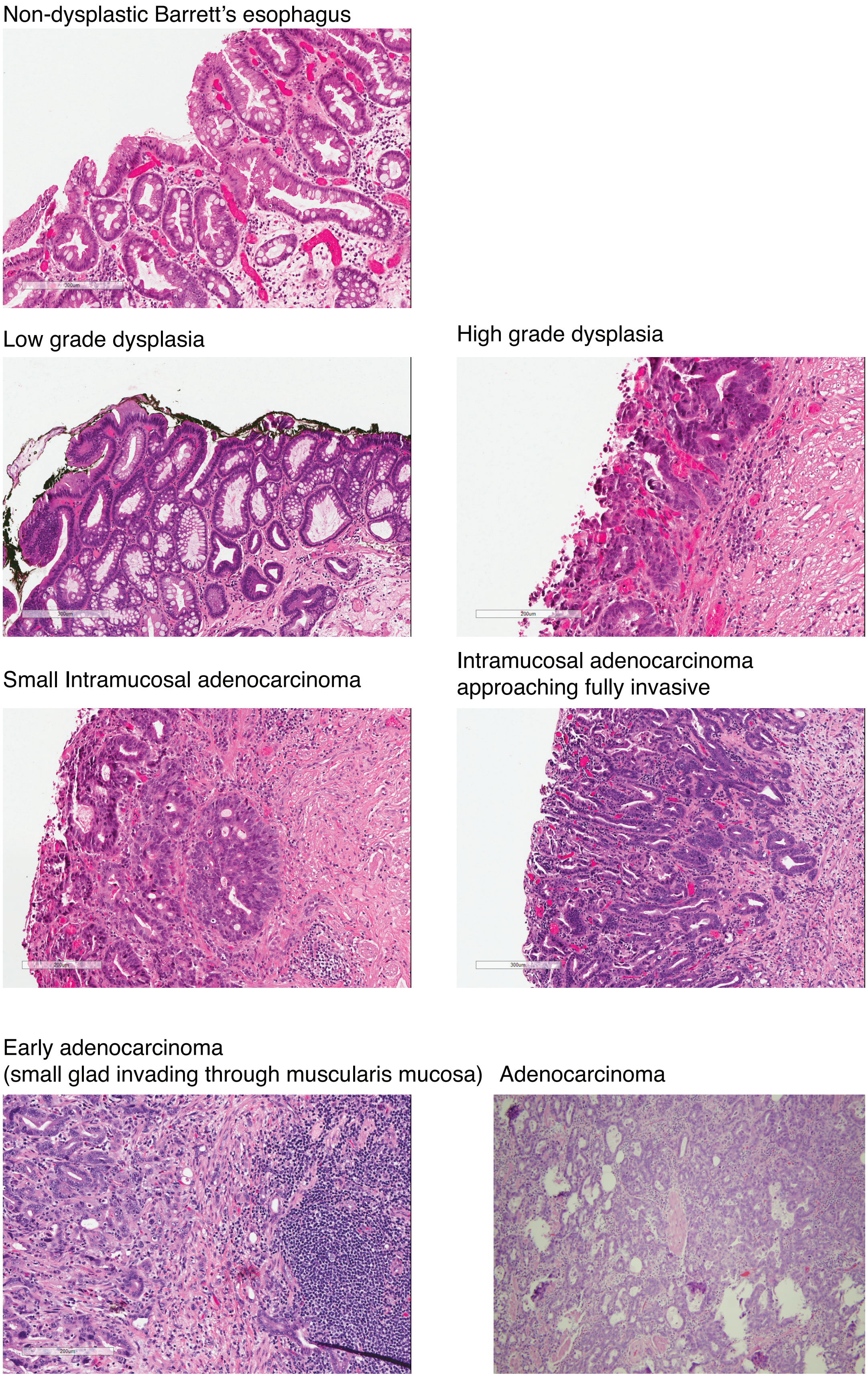

Supplementary Fig. S1. Example histopathological images of Barrett's esophagus without dysplasia (NDBE), low-grade dysplasia (LGD), high-grade dysplasia (HGD), intramucosal esophageal adenocarcinoma (IMEAC), and esophageal adenocarcinoma (EAC).

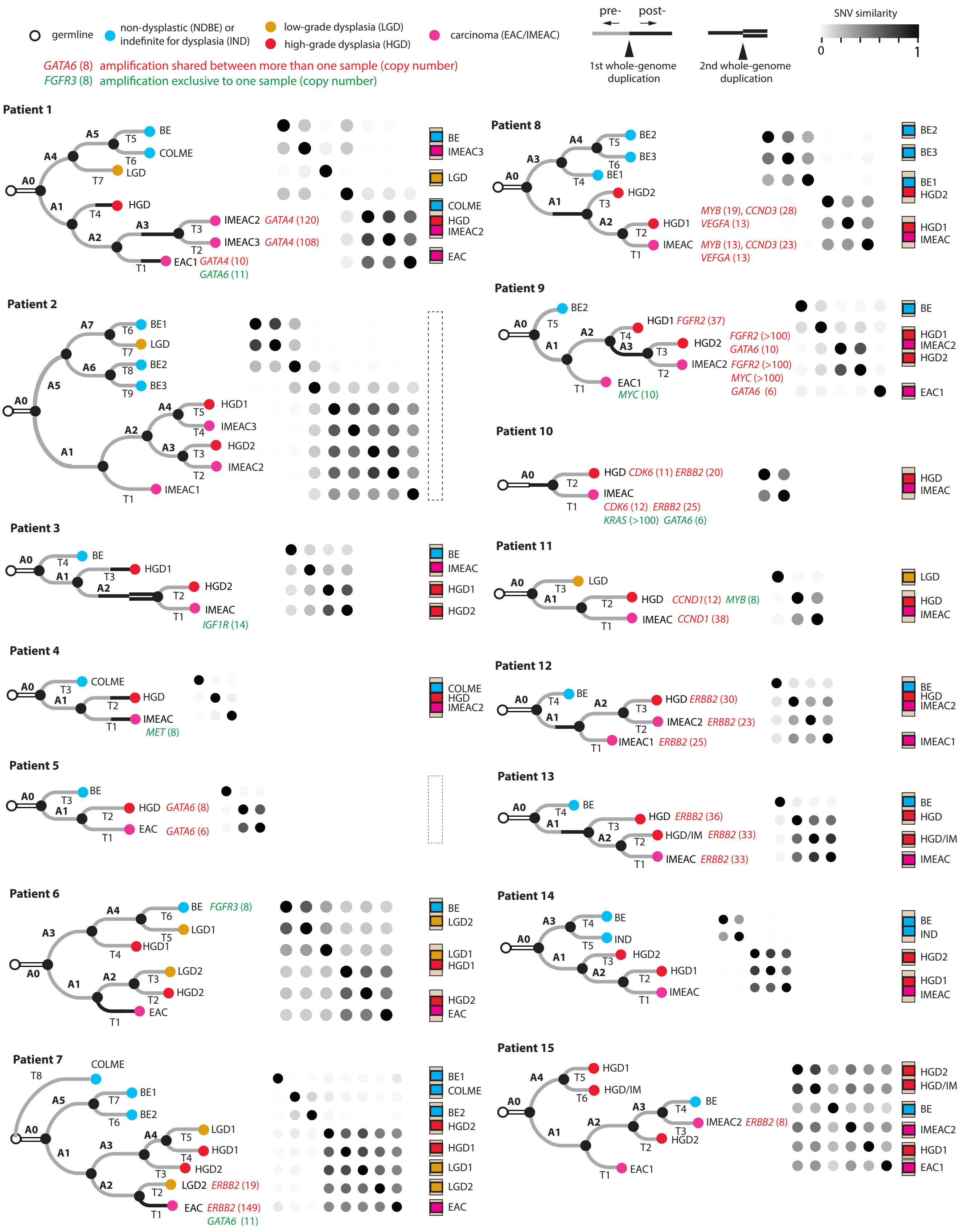

germline
non-dysplastic (NDBE) or indefinite for dysplasia (IND)
low-grade dysplasia (LGD)
high-grade dysplasia (HGD)
carcinoma (EAC/IMEAC)
pre-
post-
1st whole-genome duplication
2nd whole-genome duplication
SNV similarity
0
1
GATA6 (8) amplification shared between more than one sample (copy number)
FGFR3 (8) amplification exclusive to one sample (copy number)
Patient 1
BE COLME LGD HGD IMEAC2 GATA4 (120) IMEAC3 GATA4 (108) EAC1 GATA4 (10) GATA6 (11)
Patient 2
BE1 LGD BE2 BE3 HGD1 IMEAC3 HGD2 IMEAC2 IMEAC1
Patient 3
BE HGD1 HGD2 IMEAC IGF1R (14)
Patient 4
COLME HGD IMEAC MET (8)
Patient 5
BE HGD GATA6 (8) EAC GATA6 (6)
Patient 6
BE FGFR3 (8) LGD1 HGD1 LGD2 HGD2 EAC
Patient 7
COLME BE1 BE2 LGD1 HGD1 HGD2 LGD2 ERBB2 (19) EAC ERBB2 (149) GATA6 (11)
Patient 8
BE2 BE3 BE1 HGD2 HGD1 IMEAC MYB (19), CCND3 (28) VEGFA (13) MYB (13), CCND3 (23) VEGFA (13)
Patient 9
BE2 HGD1 FGFR2 (37) HGD2 FGFR2 (>100) GATA6 (10) IMEAC2 FGFR2 (>100) MYC (>100) GATA6 (6) EAC1 MYC (10)
Patient 10
HGD CDK6 (11) ERBB2 (20) IMEAC CDK6 (12) ERBB2 (25) KRAS (>100) GATA6 (6)
Patient 11
LGD HGD CCND1(12) MYB (8) IMEAC CCND1 (38)
Patient 12
BE HGD ERBB2 (30) IMEAC2 ERBB2 (23) IMEAC1 ERBB2 (25)
Patient 13
BE HGD ERBB2 (36) HGD/IM ERBB2 (33) IMEAC ERBB2 (33)
Patient 14
BE IND HGD2 HGD1 IMEAC
Patient 15
HGD1 HGD/IM BE IMEAC2 ERBB2 (8) HGD2 EAC1

Supplementary Fig. S2. Phylogenetic trees with branch labels (copy-number alterations along each branch are listed in Supplementary Data S2) and annotated oncogene amplifications. Also shown are the pairwise genetic similarity matrix (as heatmaps) calculated from the fraction of shared sSNVs between each pair of samples (see **Methods**). The relative spatial locations of samples are shown next to the sSNV similarity heatmap. (This information is unavailable for Patient 2 and 5). The SCNA-derived phylogenetic trees are consistent with sSNV genetic similarity with the following exceptions: HGD from Patient 1; HGD/IMEAC1/IMEAC2 from Patient 12; all samples from Patient 15. The evidential support for the phylogenetic inference in Patient 12 and 15 is presented in Suppl. Fig.S17. The HGD sample from patient 1 is a polyclonal mixture containing subclones of IMEAC cells; the grouping of HGD with cancer lesions is based on the shared *TP53* mutation and the presence of complex SCNAs in HGD that is absent in BE or LGD.

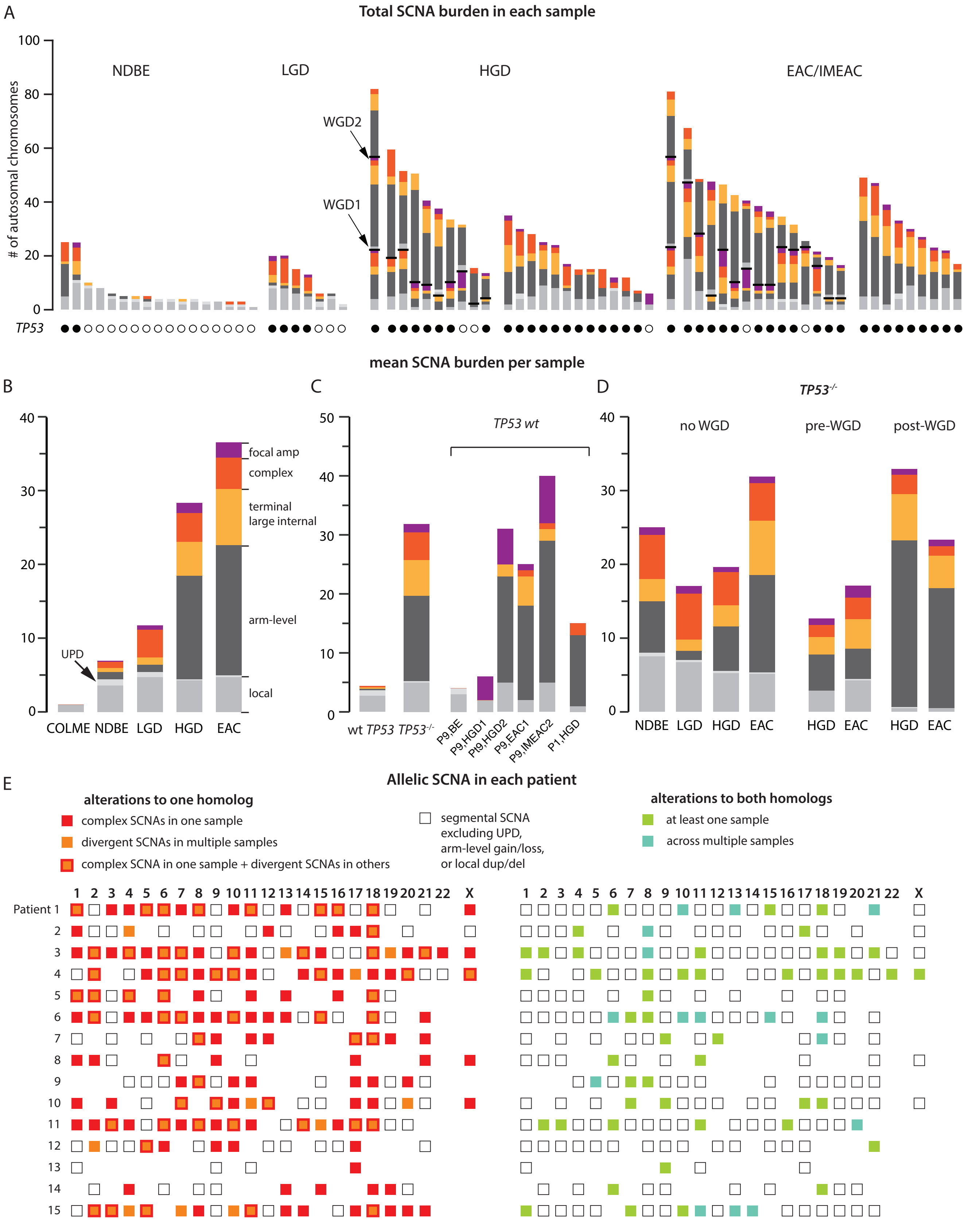
A
Total SCNA burden in each sample
# of autosomal chromosomes
NDBE
LGD
HGD
EAC/IMEAC
WGD2
WGD1
TP53
mean SCNA burden per sample
B
focal amp
complex
terminal
large internal
arm-level
local
UPD
COLME NDBE LGD HGD EAC
C
TP53 wt
wt TP53 TP53-/-
P9,BE
P9,HGD1
P9,HGD2
P9,EAC1
P9,IMEAC2
P1,HGD
D
TP53-/-
no WGD
pre-WGD
post-WGD
NDBE LGD HGD EAC HGD EAC HGD EAC
E
Allelic SCNA in each patient
alterations to one homolog
complex SCNAs in one sample
divergent SCNAs in multiple samples
complex SCNA in one sample + divergent SCNAs in others
segmental SCNA
excluding UPD,
arm-level gain/loss,
or local dup/del
alterations to both homologs
at least one sample
across multiple samples
Patient 1
X

Supplementary Fig. S3. Landscape of somatic copy-number alterations (SCNA) in BE and EAC lesions. **A**. Total SCNA burden (number of paternal and maternal autosomes with SCNAs) in each sample broken down by SCNA type and timing relative to whole-genome duplication (WGD). Local deletion/duplication (<100kb), uniparental disomy (UPD), and arm-level SCNAs are colored the same as in **Main Figure** 4. Complex segmental copy-number changes are further subdivided to (1) terminal, paracentric, and pericentric gain/loss (orange); (2) complex alterations (red); and (3) focal amplifications (purple). Samples are grouped based on histopathological grading (non-dysplastic BE, low-grade dysplasia, high-grade dysplasia, and carcinoma). The mutation status of *TP53* is shown below each sample: black circles for bi-allelic inactivation, open circles for no identifiable alterations or mono-allelic inactivation. **B**. Mean SCNA burden of samples in each histopathological group. **C**. Mean SCNA burden in samples with intact or inactive p53. The SCNA burdens in five HGD/EAC samples (four from Patient 9, one from Patient 1) without evidence of biallelic *TP53* inactivation are shown separately from NDBE/LGD samples without *TP53* inactivation. These five samples are excluded in the left plot or in **Main Fig. 4A** (middle). **D**. Mean SCNA burden in samples without whole-genome duplication (WGD) or inferred to have occurred prior to or after WGD. **E**. Allelic distribution of SCNAs on each chromosome (Chrs.1-22, X) in each patient. Open boxes denote the presence of segmental SCNAs. Colored boxes denote the allelic composition of multiple SCNA breakpoints: red/orange for the presence of SCNAs phased to a single parental chromosome (left); blue/green for the presence of SCNAs on both parental chromosomes (right). The allelic patterns of SCNA breakpoints are further classified based on their presence in a single sample or different samples from the same patient. For SCNAs arising independently, they should more frequently affect both homologs than accrue on a single homolog. The predominance of mono-allelic SCNAs over bi-allelic SCNAs indicates a concentration of breakpoints on a single homolog that is consistent with catastrophic events or successive changes on a single unstable chromosome.

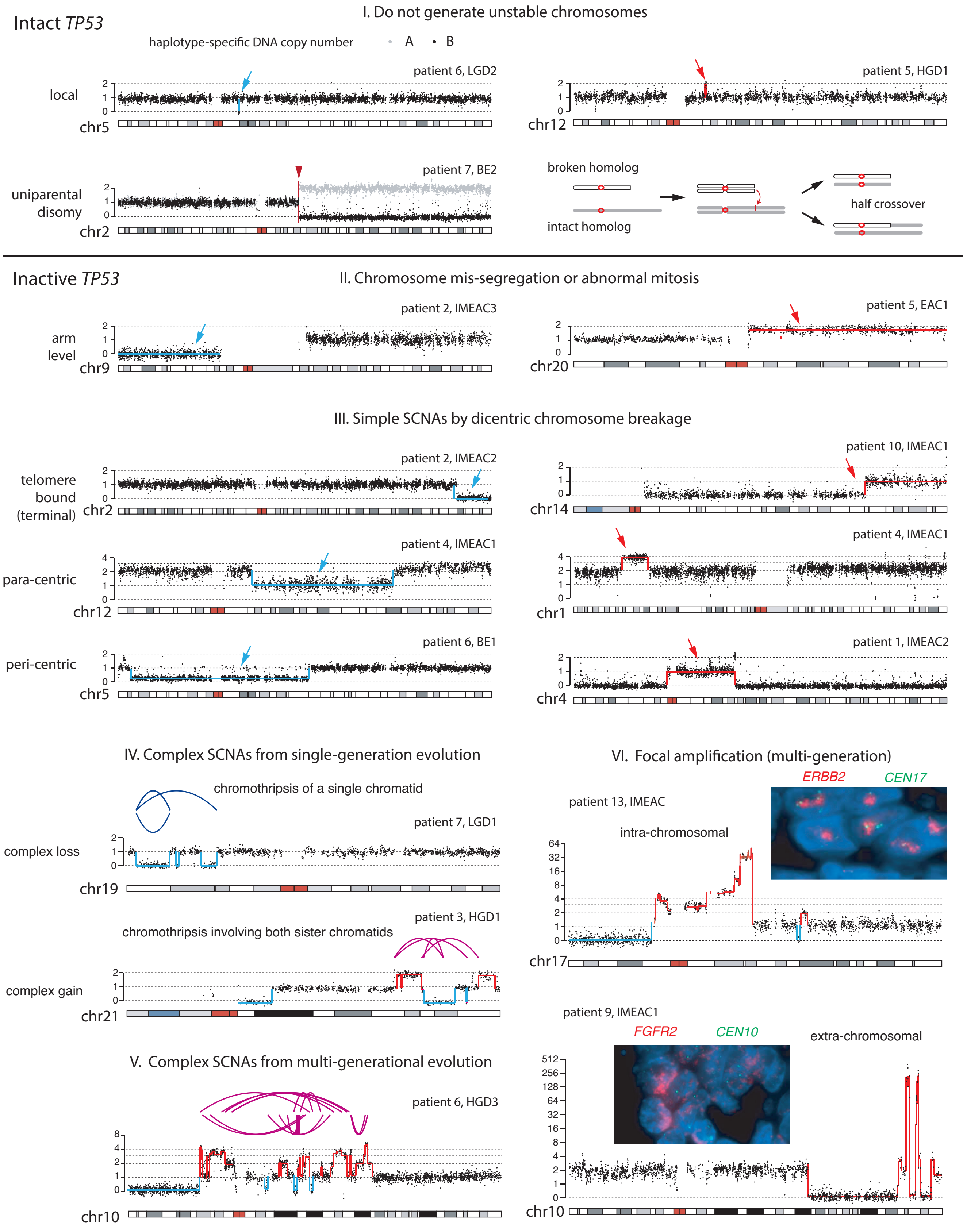

Intact TP53
I. Do not generate unstable chromosomes
haplotype-specific DNA copy number
A
B
patient 6, LGD2
patient 5, HGD1
local
chr5
chr12
patient 7, BE2
uniparental disomy
chr2
broken homolog
intact homolog
half crossover
Inactive TP53
II. Chromosome mis-segregation or abnormal mitosis
patient 2, IMEAC3
patient 5, EAC1
arm level
chr9
chr20
III. Simple SCNAs by dicentric chromosome breakage
patient 2, IMEAC2
patient 10, IMEAC1
telomere bound (terminal)
chr2
chr14
patient 4, IMEAC1
patient 4, IMEAC1
para-centric
chr12
chr1
patient 6, BE1
patient 1, IMEAC2
peri-centric
chr5
chr4
IV. Complex SCNAs from single-generation evolution
chromothripsis of a single chromatid
patient 7, LGD1
complex loss
chr19
patient 3, HGD1
chromothripsis involving both sister chromatids
complex gain
chr21
V. Complex SCNAs from multi-generational evolution
patient 6, HGD3
chr10
VI. Focal amplification (multi-generation)
ERBB2
CEN17
patient 13, IMEAC
intra-chromosomal
chr17
patient 9, IMEAC1
FGFR2
CEN10
extra-chromosomal
chr10

Supplementary Fig. S4. SCNA patterns based on the mutational mechanisms. I. Local DNA deletion/duplication and uniparental disomy (UPD) that do not generate unstable (acentric/dicentric) chromosomes. These events comprise a majority of alterations seen in cells or clones with intact p53. We suggest UPDs may arise from homology-dependent invasion of a broken chromatid into the intact homolog followed by a "half crossover" with an opposite moving replication fork, instead of conservative, unidirectional break-induced replication to the chromosome end. II-IV. Copy-number alterations resulting from single chromosome missegregation events. II. Arm-level copy-number changes are generated by chromosome mis-segregation or abnormal cell divisions, including cytokinesis failure and multipolar cell division (Suppl. Fig. S5A). III. Terminal, paracentric, and pericentric segmental copy-number alterations can be generated by the breakage of different types of dicentric chromosomes (**Main Figure** 5). IV. Dicentric chromosome breakage or DNA damage in micronuclei (Suppl. Fig. S5B,C) can lead to chromosome fragmentation and create two-state (single-chromatid fragmentation) or three-state (sister-chromatids fragmentation) oscillating copy-number patterns. V and IV. Complex gains and focal amplifications resulting from multiple generations of chromosome breakage and asymmetric distribution (Suppl. Fig. S5D). V. Chromothripsis or telomere loss can initiate multi-generational breakage-fusion-bridge cycles with concomitant chromothripsis that generate complex copy-number gains (>2 copy-number states) or focal amplifications. VI. Focally amplified DNA can be either intrachromosomal (e.g., generated by intrachromosomal BFBs) or extrachromosomal (e.g., acentric fragments from chromothripsis).

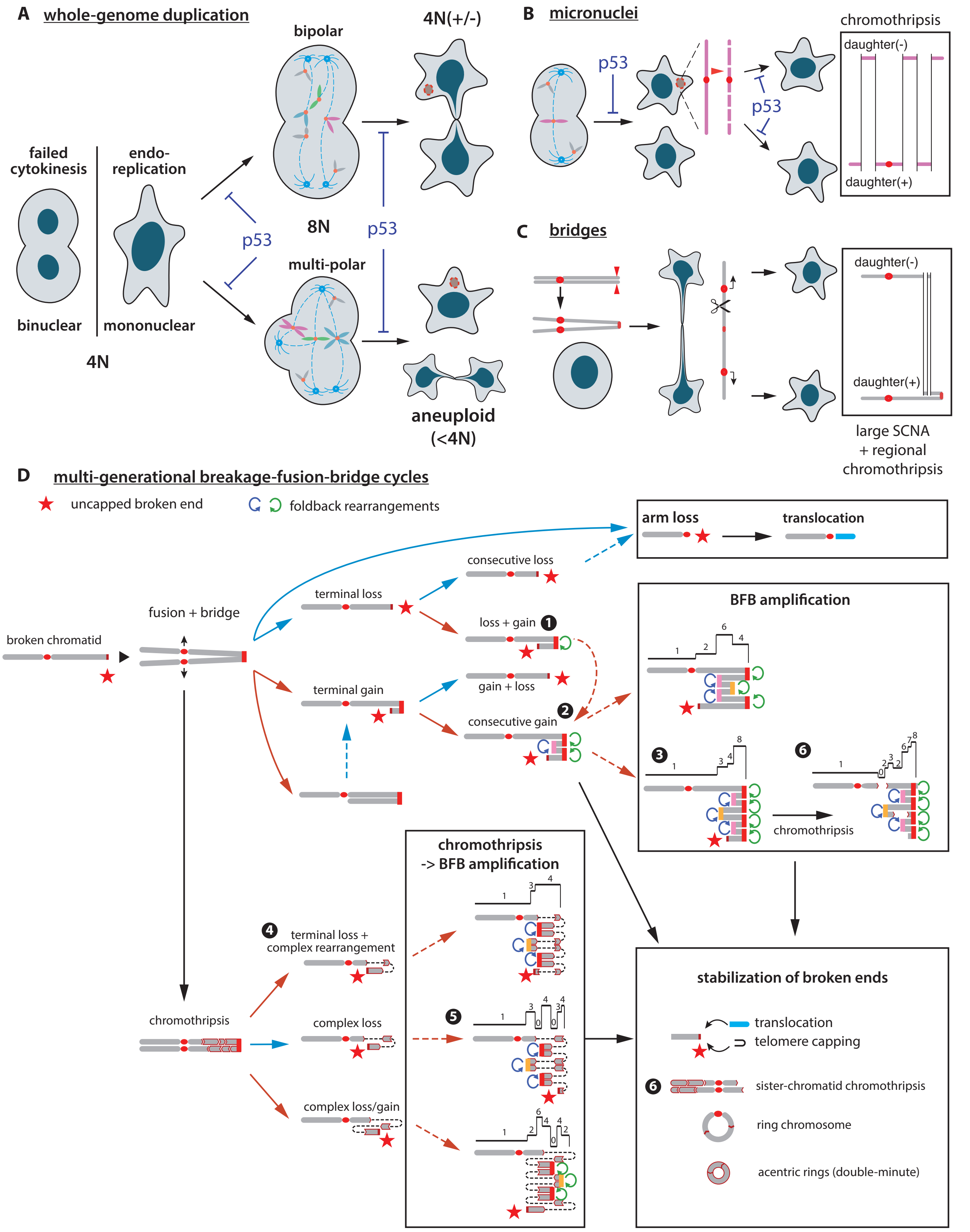
A whole-genome duplication
failed cytokinesis
endo-replication
binuclear
mononuclear
4N
p53
bipolar
8N
multi-polar
p53
4N(+/-)
aneuploid (<4N)
B micronuclei
p53
p53
chromothripsis
daughter(-)
daughter(+)
C bridges
daughter(-)
daughter(+)
large SCNA + regional chromothripsis
D multi-generational breakage-fusion-bridge cycles
uncapped broken end
foldback rearrangements
broken chromatid
fusion + bridge
terminal loss
consecutive loss
loss + gain
terminal gain
gain + loss
consecutive gain
arm loss
translocation
BFB amplification
chromothripsis
chromothripsis
chromothripsis -> BFB amplification
terminal loss + complex rearrangement
complex loss
complex loss/gain
stabilization of broken ends
translocation
telomere capping
sister-chromatid chromothripsis
ring chromosome
acentric rings (double-minute)

Supplementary Fig. S5. Copy-number outcomes of tetraploidization (A), micronucleation (B), breakage of a dicentric chromosome (C), and multigenerational evolution (including successive breakage-fusion-bridge cycles and chromothripsis) of dicentric chromosomes (D) determined from experimental analyses of chromosomal instability. **A**. Tetraploid (4N) cells can arise from failed cytokinesis or endoreplication (DNA replication without mitosis). The 4N cell has duplicated centrosomes that can both cause downstream chromosome missegregation and generate highly aneuploid genomes after multipolar cell divisions. Multiple steps of this evolutionary process are suppressed by p53. **B**. Chromosome fragmentation in micronuclei can generate chromothripsis with oscillating DNA copy number in both daughter cells. **C**. Resolution of dicentric bridge chromosomes results in reciprocal gain and loss of a telomere-bound segment (chromatid-type fusion) or a large internal segment (chromosome-type fusion, **Main Fig.** 5B), with occasional DNA fragmentation near the breakage site leading to local chromothripsis (see Suppl. Fig. S8A,B for examples in BE/EAC genomes). **D**. Multigenerational evolution of a broken chromosome through breakage-fusion-bridge (BFB) cycles and chromothripsis. (Top) Copy-number outcomes of BFB cycles without chromosome fragmentation. (Bottom) Copy-number outcomes of BFB cycles after ancestral chromothripsis. The unstable broken chromosome can become mitotically stable after acquiring a telomere through de novo telomere addition, translocation to a telomeric segment, or chromothripsis of a pair of sister chromatids with two telomeric segments. The broken chromosome can also give rise to an unstable ring chromosome (after loss of both telomeres) or acentric extrachromosomal circles (after centromeric loss), both of which can generate high-level gene amplifications.

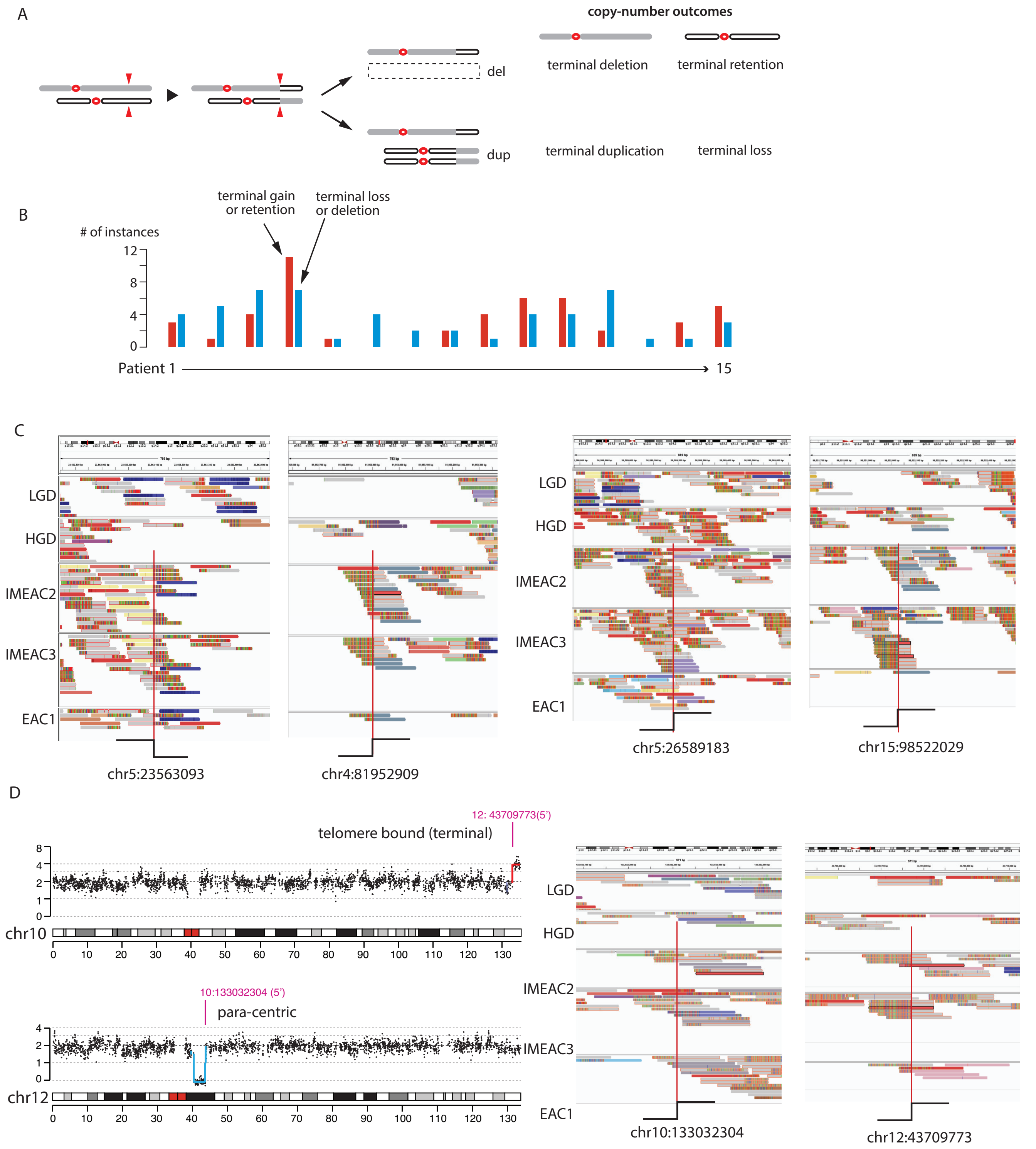

Supplementary Fig. S6. Additional evidence for the connection between dicentric chromosome breakage and terminal DNA loss and gain. **A**. An alternative model for generating terminal SCNAs is by a reciprocal translocation followed by whole-chromosome gain or loss. This sequence can generate single-copy terminal losses (of the filled chromatid) or terminal gains (of the open chromatid), but the number of terminal losses and terminal gains (including segmental retentions) must be strictly identical. **B**. The disparity between terminal losses and terminal gains (only single-copy alterations) suggests that at least a subset of these alterations did not arise from reciprocal translocations. **C**. Integrative Genome Viewer (IGV) snapshots of supporting discordant reads of two translocations of chr5 as shown in **Main Fig. 6B**. **D**. A rearrangement junction between the breakpoint of the 10q-terminal gain and the telomeric breakpoint of the paracentric deletion on 12q. This junction cannot reflect a simple translocation as the translocated chromosome consisting of the 12q terminal segment and the 10q segment would be acentric and therefore not stably propagated.

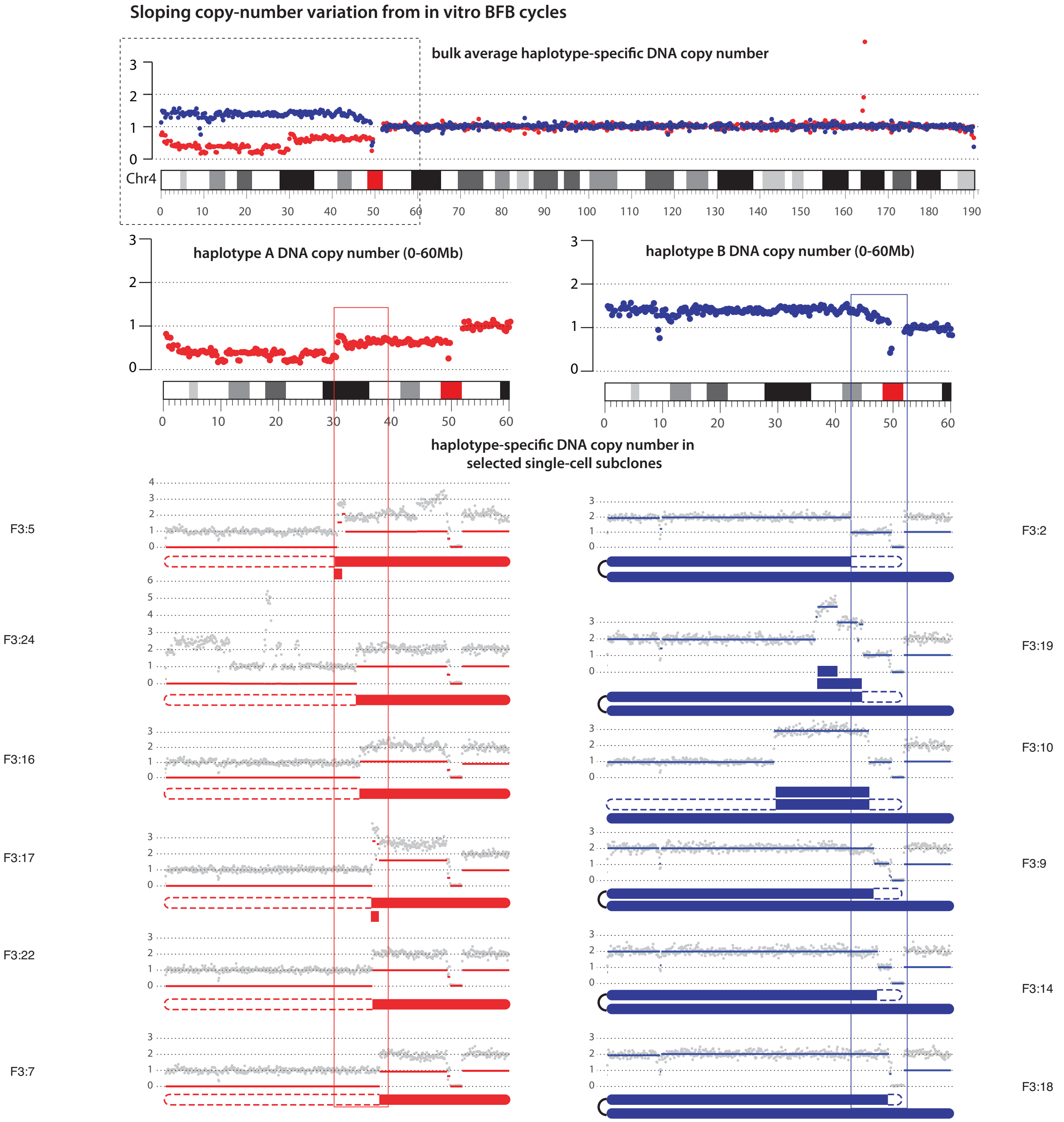

Supplementary Fig. S7. Gradual (sloping) copy-number variation on chr4p in the progeny population of a single cell with a broken chr4 engineered by CRISPR/Cas9 [Primary Clone 2b in Umbreit et al. (2020) [9]]. Shown are the bulk average DNA copy number (top) and the DNA copy number of subclones generated from single cells (bottom). Blue and red dots in the bulk average DNA copy-number plots represent the DNA copy number (100kb intervals) of each parental haplotype. In the DNA copy-number plots of single subclones, gray dots represent total sequence coverage (100kb intervals), red and blue lines represent segmented haplotype-specific DNA copy number of the homolog with progressive DNA losses; the other homolog is omitted. Segmental copy-number alterations are shown below the DNA copy-number plots. The copy-number plots on the left and on the right are from different subclones.

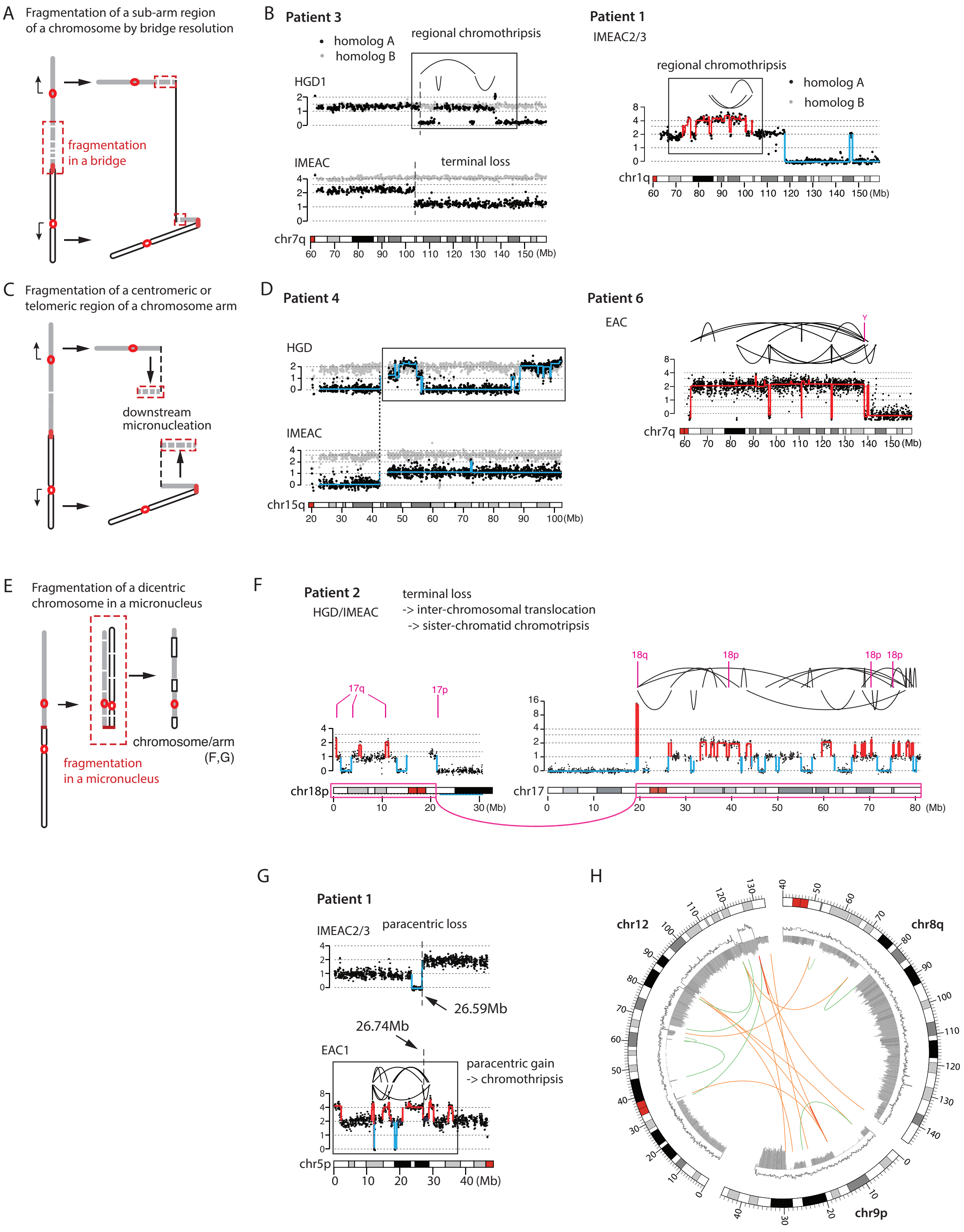
A
Fragmentation of a sub-arm region of a chromosome by bridge resolution
fragmentation in a bridge
B
Patient 3
homolog A
homolog B
regional chromothripsis
HGD1
IMEAC
terminal loss
chr7q
Patient 1
IMEAC2/3
regional chromothripsis
homolog A
homolog B
chr1q
C
Fragmentation of a centromeric or telomeric region of a chromosome arm
downstream micronucleation
D
Patient 4
HGD
IMEAC
chr15q
Patient 6
EAC
chr7q
E
Fragmentation of a dicentric chromosome in a micronucleus
chromosome/arm (F,G)
fragmentation in a micronucleus
F
Patient 2
HGD/IMEAC
terminal loss
-> inter-chromosomal translocation
-> sister-chromatid chromotripsis
17q
17p
18q
18p
chr18p
chr17
G
Patient 1
IMEAC2/3
paracentric loss
26.59Mb
26.74Mb
EAC1
paracentric gain
-> chromothripsis
chr5p
H
chr12
chr8q
chr9p

Supplementary Fig. S8. Copy-number patterns reflecting chromothripsis from different evolutionary sequences. **A**. A schematic model of regional chromothripsis resulting from dicentric chromosome breakage. **B**. (Left, top) Chromothripsis spanning a region next to a terminal deletion and duplication in HGD1 of Patient 3; (Left, bottom) terminal deletion in the same region in IMEAC. The haplotype phase in this region was determined based on allelic imbalance in IMEAC. (Right) Chromothripsis in a region of paracentric gain that was likely generated in a chromosome-type BFB cycle. **C**. A schematic model of regional chromothripsis resulting from DNA fragmentation in micronuclei containing broken bridge chromosomes. **D**. (Left) An example of chromothripsis spanning a telomeric segment after an ancestral paracentric deletion. (Right) Chromothripsis on 7q with a terminal deletion showing clustering of breakpoints near the boundaries of interspersed deletions. The clustering pattern resembles the "tandem-short-templates" (TST) signature reported in Umbreit et al. (2020)[9]. **E**. A schematic model of arm-level chromothripsis resulting from fragmentation of a dicentric chromosome in a micronucleus. **F**. Chromothripsis of a dicentric chromosome t(18p;17q) leading to three-state copy-number oscillation in both chromosome arms. **G**. Chromothripsis of 5p in EAC1 with three copy-number states includes a copy-number breakpoint at 26.59Mb that is complementary to the breakpoint at 26.74Mb in IMEAC2/3 (dashed lines). The paracentric deletion in IMEAC2 suggests an ancestral chromosome-type BFB cycle; the complementary breakpoints in EAC1 and IMEAC2/3 suggests that the chromothripsis may have arisen downstream after the ancestral breakage. **H**. Interchromosomal rearrangements and chromothripsis in Patient 7 with clustered rearrangement breakpoints that resemble the TST signature. Loci with more than one breakpoint include chr8:73Mb, chr9:4Mb and 15Mb, chr12:89Mb, 117Mb, and 127Mb. All copy-number plots except in B and C only show the altered homolog. The complete plots of both homologs and for other examples of the same evolution sequences of BFB cycles and chromothripsis are available in the Online Data Repository.

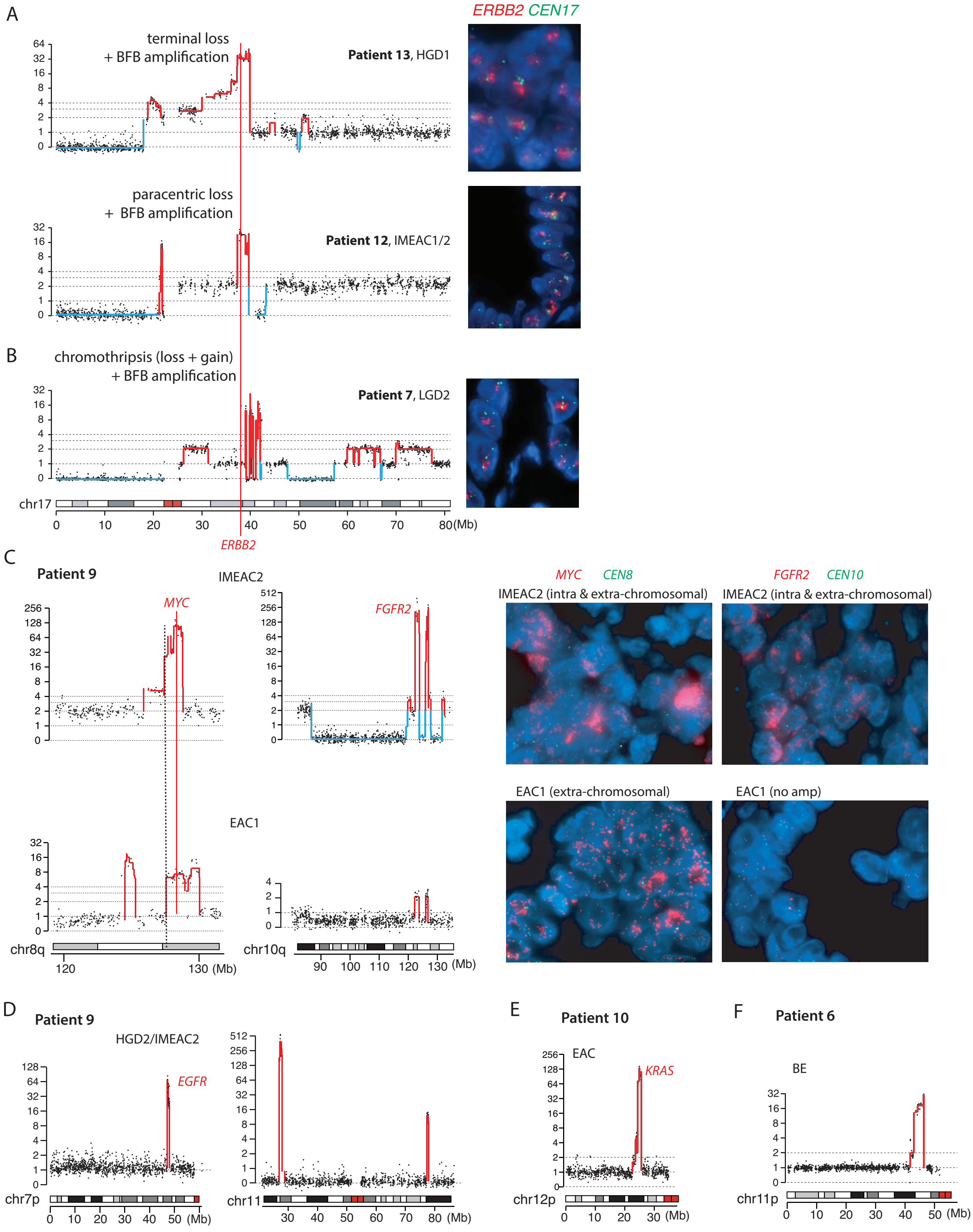
A
ERBB2 CEN17
terminal loss
+ BFB amplification
Patient 13, HGD1
paracentric loss
+ BFB amplification
Patient 12, IMEAC1/2
B
chromothripsis (loss + gain)
+ BFB amplification
Patient 7, LGD2
chr17
0 10 20 30 40 50 60 70 80(Mb)
ERBB2
C
Patient 9
IMEAC2
MYC
FGFR2
MYC CEN8
FGFR2 CEN10
IMEAC2 (intra & extra-chromosomal)
IMEAC2 (intra & extra-chromosomal)
EAC1
EAC1 (extra-chromosomal)
EAC1 (no amp)
chr8q
120 130 (Mb)
chr10q
90 100 110 120 130 (Mb)
D
Patient 9
HGD2/IMEAC2
EGFR
chr7p
0 10 20 30 40 50 (Mb)
chr11
30 40 50 60 70 80 (Mb)
E
Patient 10
EAC
KRAS
chr12p
0 10 20 30 (Mb)
F
Patient 6
BE
chr11p
0 10 20 30 40 50 (Mb)

Supplementary Fig. S9. Selected examples of focal amplifications in BE/EAC genomes. **A** and **B**. Intrachromosomal amplification of *ERBB2* consistent with successive BFB cycles. (Left) DNA copy number of chr17 showing ascending copy-number gains near *ERBB2* with 17q-terminal loss is consistent with BFB amplifications. The presence of interspersed deletions within the region of amplification in **B** indicates chromothripsis prior to amplification. (Right) Fluorescence in-situ hybridization (FISH) analysis showing amplified *ERBB2* in homogeneously staining regions (HSRs) next to chr17 centromeres as expected for intrachromosomal BFB amplification. **C**. (Left) Distinct focal amplifications spanning *MYC* on 8q and *FGFR2* on 10q in EAC and IMEAC of Patient 9. The shared boundary of amplified regions on 8q in both genomes suggests an ancestral chromosome breakage event with divergent downstream alterations. The localization of oscillating DNA deletion and amplification on 10q in IMEAC2 indicates regional chromothripsis preceding the amplification. The sharp copy-number transitions between deleted and amplified regions with few intermediate states are consistent with random segregation of intact double-minute chromosomes/ecDNA circles. (Right) FISH analysis shows amplified *MYC* and *FGFR2* in both extrachromosomal and intrachromosomal DNA. **D**. Additional examples of focal amplifications on 7p (spanning *EGFR*) and chr11 in HGD2 and IMEAC2 of Patient 9. **E**. Amplification of *KRAS* in Patient 10 that is specific to EAC but not in HGD. **F**. Amplification on 11p in a non-dysplastic BE lesion with *TP53* inactivation in Patient 6.

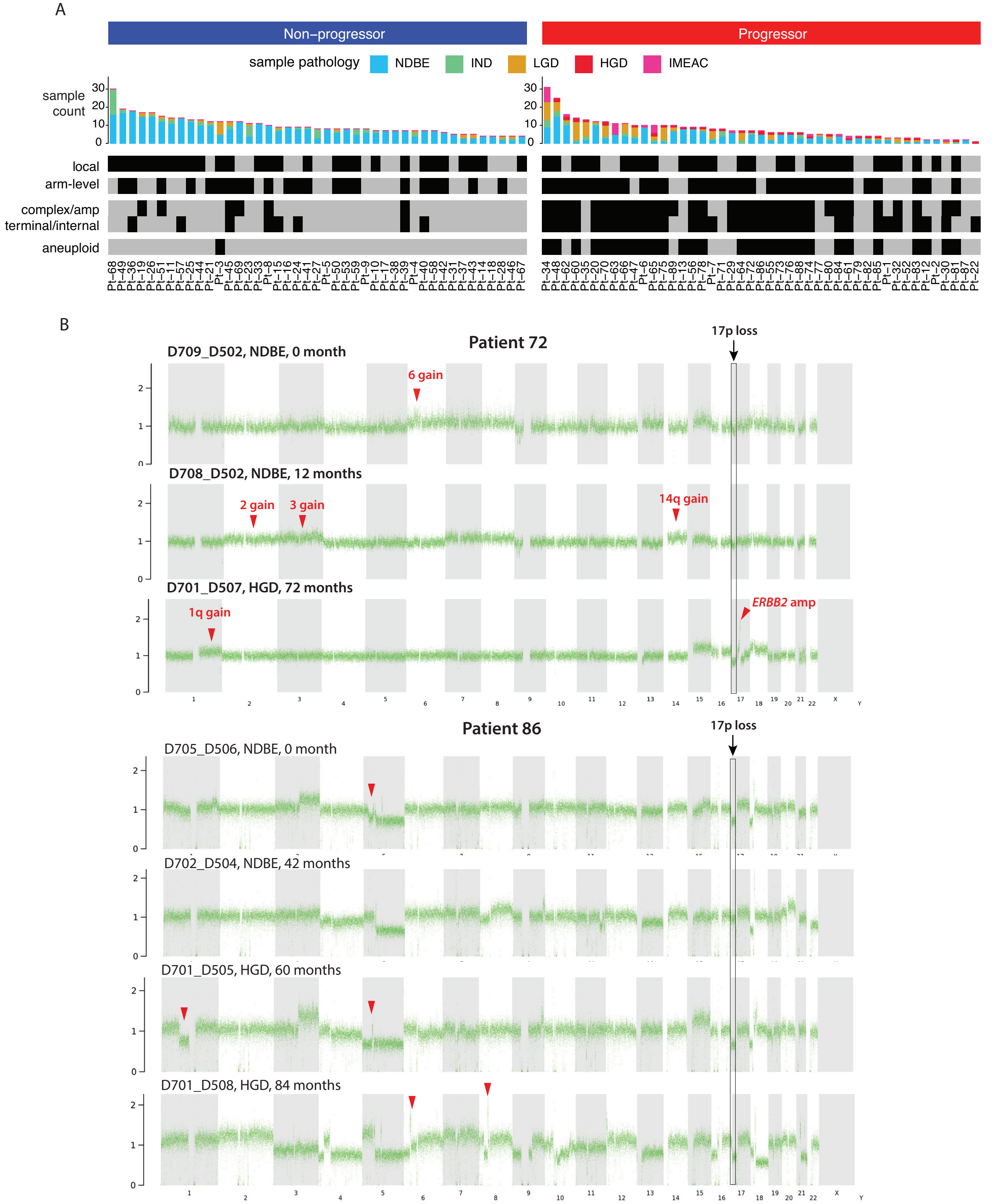

Supplementary Fig. S10. BE copy-number evolution revealed from low-pass sequencing of longitudinal BE samples from Killcoyne et al. (2020). **A**. Association between BE progression and copy-number features including (1) local deletion/duplication; (2) arm-level gain/loss; (3) simple and complex segmental SCNAs; and (4) extensive aneuploidy (five or more chromosomes with either arm-level or segmental SCNAs). For each patient, both non-progressors (left) and progressors (right), the copy-number features are assessed from all longitudinal BE/EAC samples, the number of which is shown above the copy-number feature heatmap and separated by pathological grading. The most distinguishing features are the presence of aneuploidy ($p = 10^{-10}$), complex SCNAs/focal amplifications ($p = 10^{-8}$) and segmental SCNAs ($p = 10^{-7}$). Sporadic arm-level alterations ($p = 0.05$) or local deletion/duplication events ($p = 0.83$) have similar prevalence between the two groups. All p–values are calculated by Fisher's exact test. **B**. Genome-wide DNA copy-number profiles of selected samples from Patient 72 and Patient 86 (both progressors) showing ancestral 17p losses (causing hemizygous p53 loss) and many private SCNAs (red arrows) indicating branching copy-number evolution. Mutations in *TP53* cannot be detected due to the limited sequencing depth.

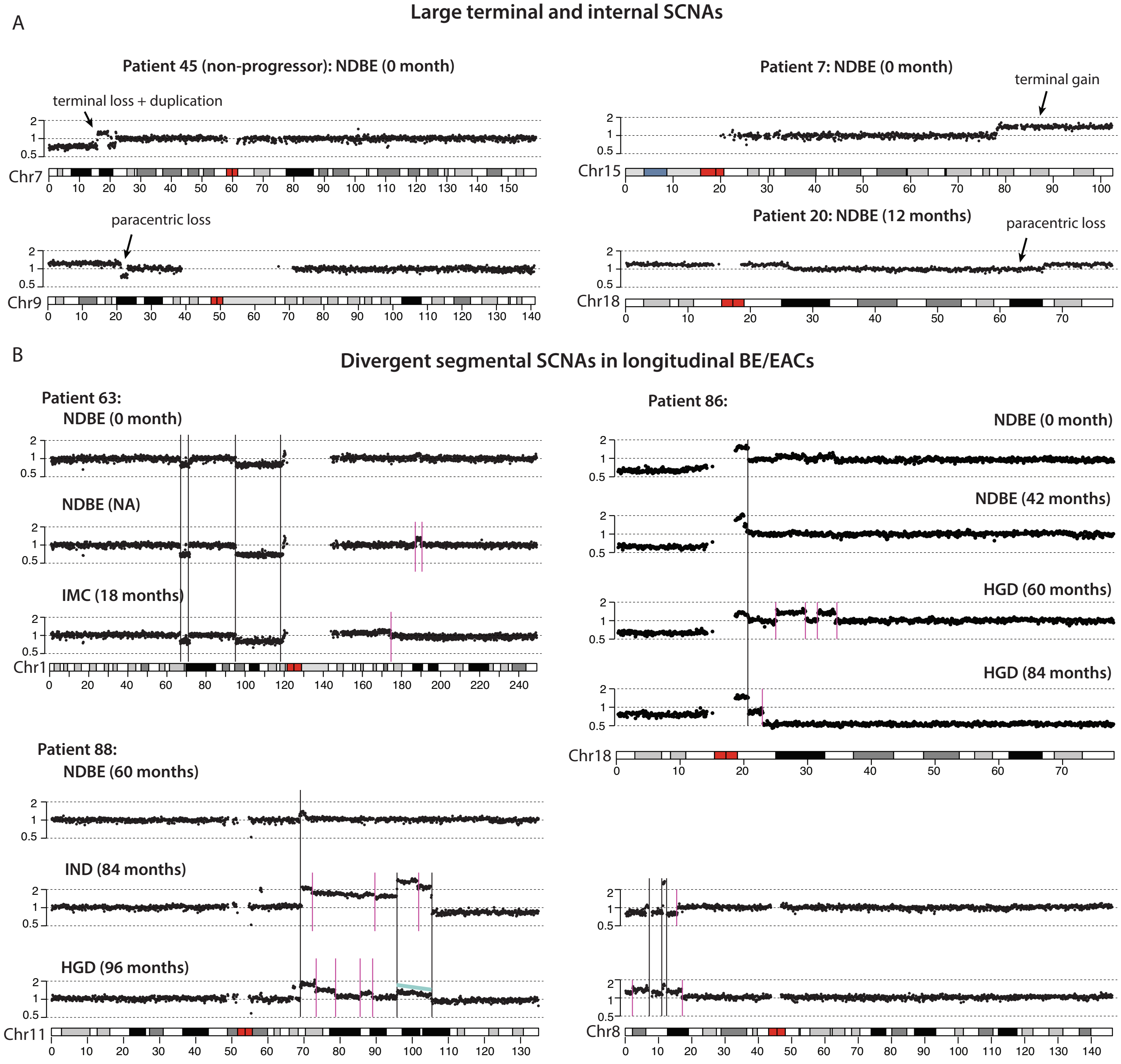

Supplementary Fig. S11. Evidence of chromosomal instability in BE cells from longitudinal BE sequencing. **A**. Representative examples of large terminal (top) and internal (bottom) SCNAs in non-dysplastic BE samples. Notably, the left two examples were taken from a non-dysplastic BE sample from a non-progressor (Patient 45). Although we cannot rule out the possibility that this patient may eventually develop advanced EAC, the absence of EAC or high-grade BE at 60 months after the initial NDBE biopsy indicates chromosomal instability being present at a very early stage of BE progression. This patient does not show 17p loss that is the most recurrent alteration in progressors (see the previous figure for two examples) and contributes to p53 loss. The lack of complex segmental gains or copy-number heterogeneity also suggests limited chromosomal instability in the ancestor cell where the observed alterations first arose. **B**. Representative examples of chromosomes with both shared (solid lines) and private SCNA breakpoints (magenta lines) in longitudinal BE samples from three progressors. Note the sloping copy-number pattern in the HGD lesion (96 months) from Patient 88 in a region on chr11q that displays clonal (discrete) copy-number changes in a BE with indefinite histopathology (IND) at 84 months.

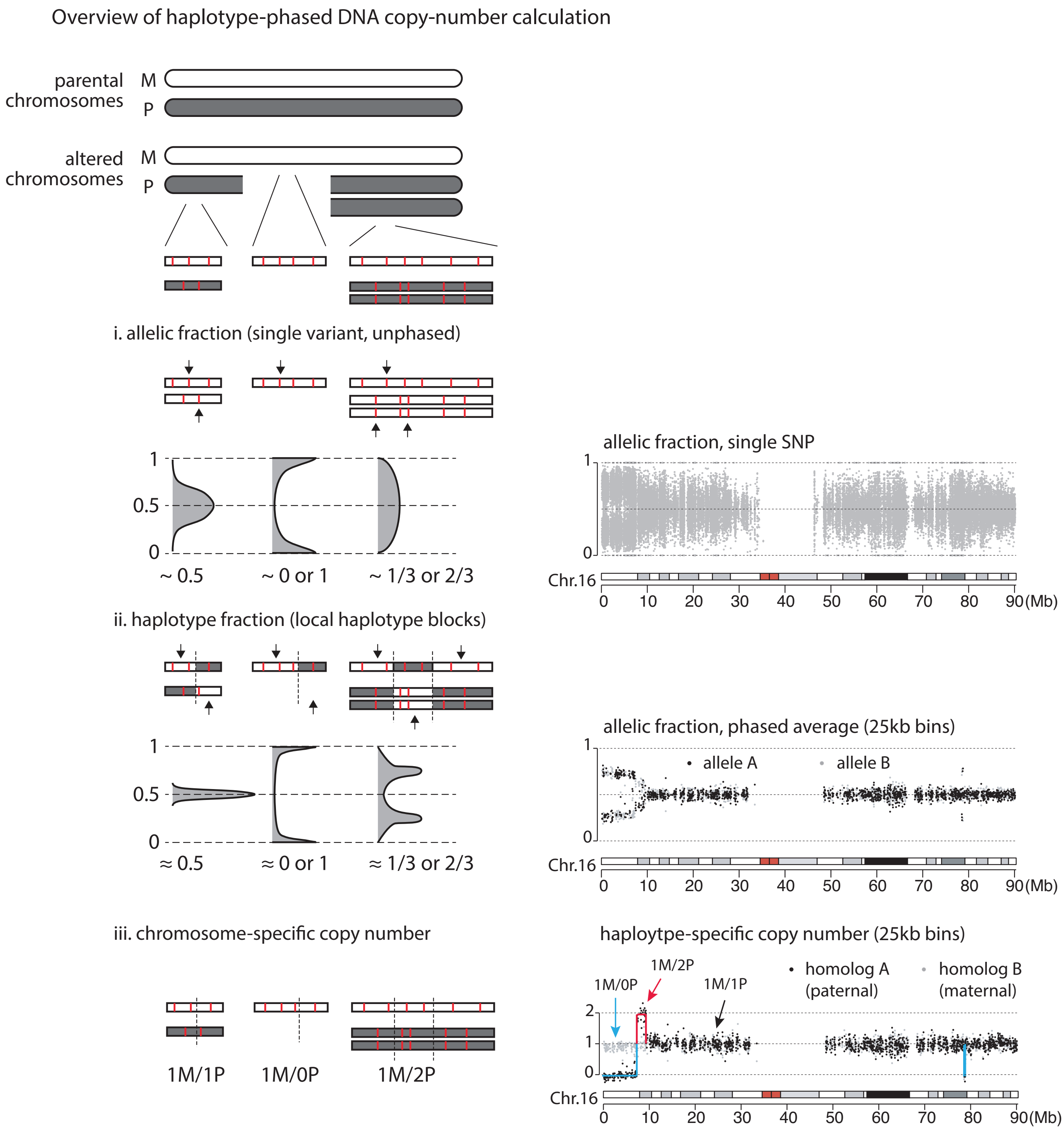

Supplementary Fig. S12. General idea of haplotype-specific DNA copy-number calculation based on (i) allelic fractions at single polymorphic sites; (ii) phased average allelic fractions in local haplotype blocks; and (iii) chromosome-specific allelic fractions. (Left) Schematic illustration of expected results; (right) real data from Chr.16 in the HGD2 sample from Patient 4.

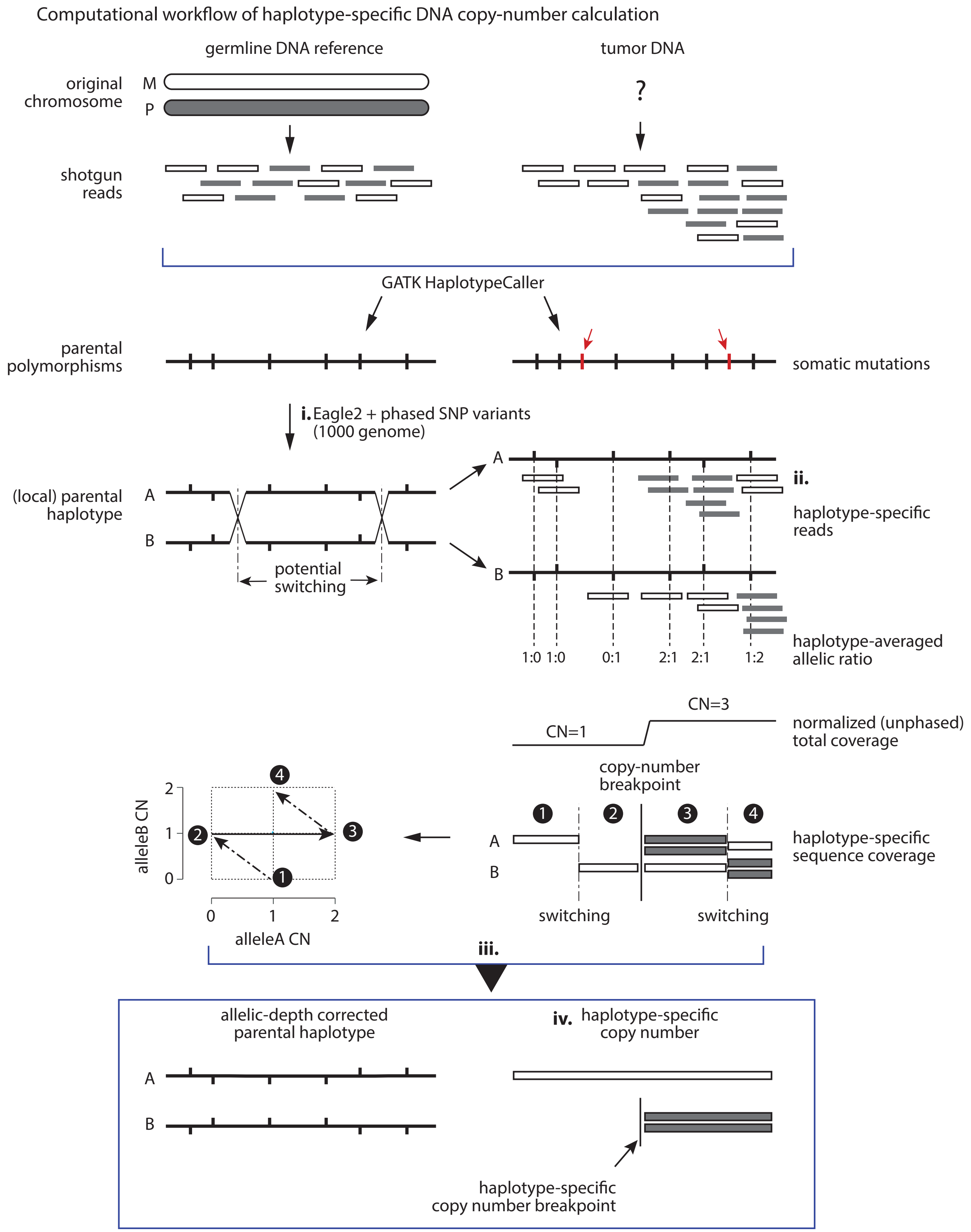

Supplementary Fig. S13. Overview of the computational workflow of haplotype-specific copy-number calculation: (i) statistical haplotype phasing (middle, left); (ii) calculation of local allelic fractions and allelic coverage (middle, right); (iii) correction of haplotype switching errors by allelic imbalance (lower middle); (iv) final haplotype copy-number calculation (bottom).

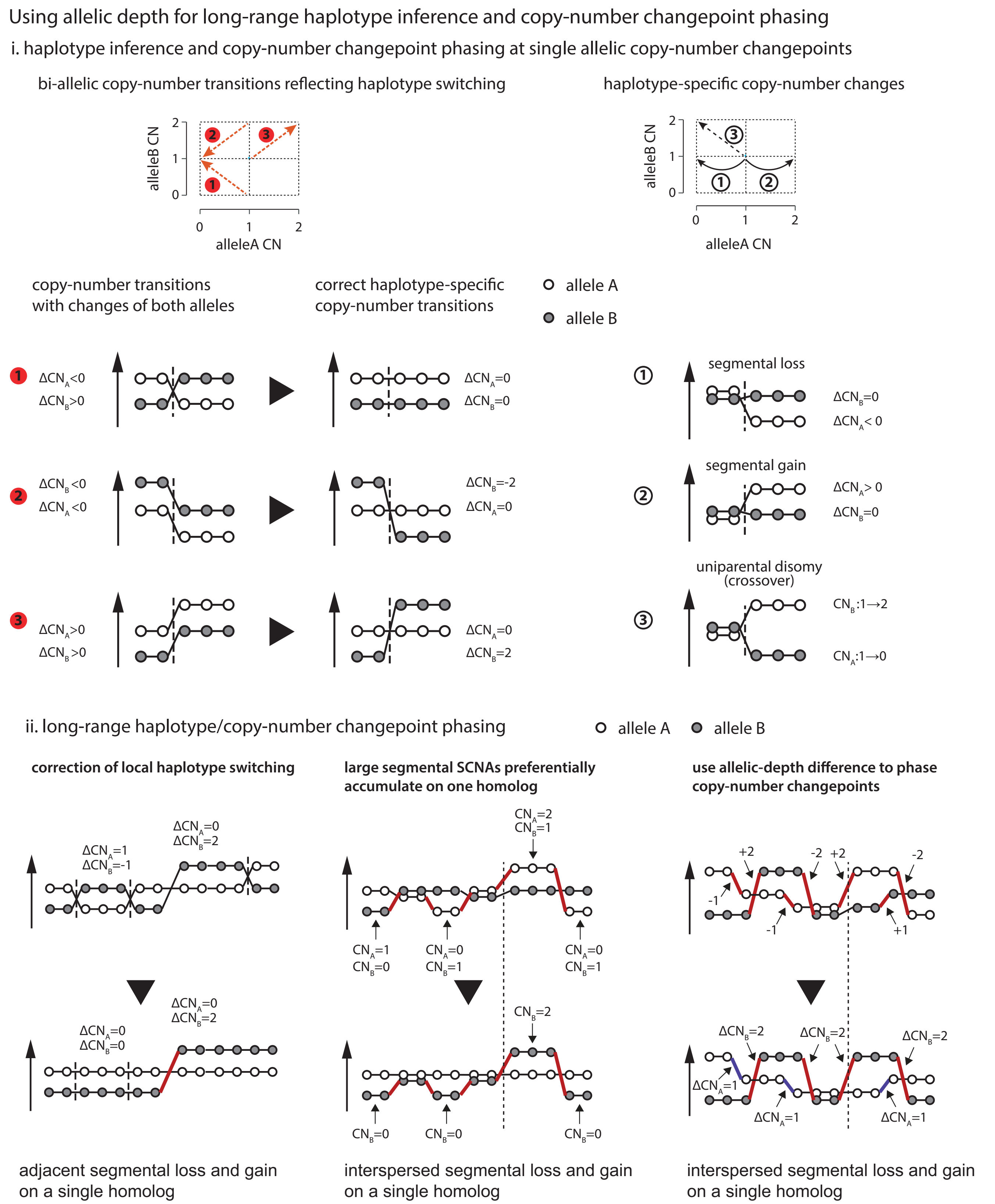

Supplementary Fig. S14. Strategies of long-range haplotype inference by allelic imbalance: i. allelic copy-number transitions due to haplotype switching errors (left, changes in both allelic depths) and reflecting true somatic copy-number alterations (right, changes in only one allelic depth, except uniparental disomy); ii. long-range phasing of copy-number alterations/changepoints based on continuous allelic imbalance (left), allelic imbalance with interspersed allelic balance (middle), and allelic imbalance with different allelic ratios (right). Corrected long-range switching errors are marked with dashed lines.

Examples of long-range haplotype inference and copy-number changepoint phasing in single samples

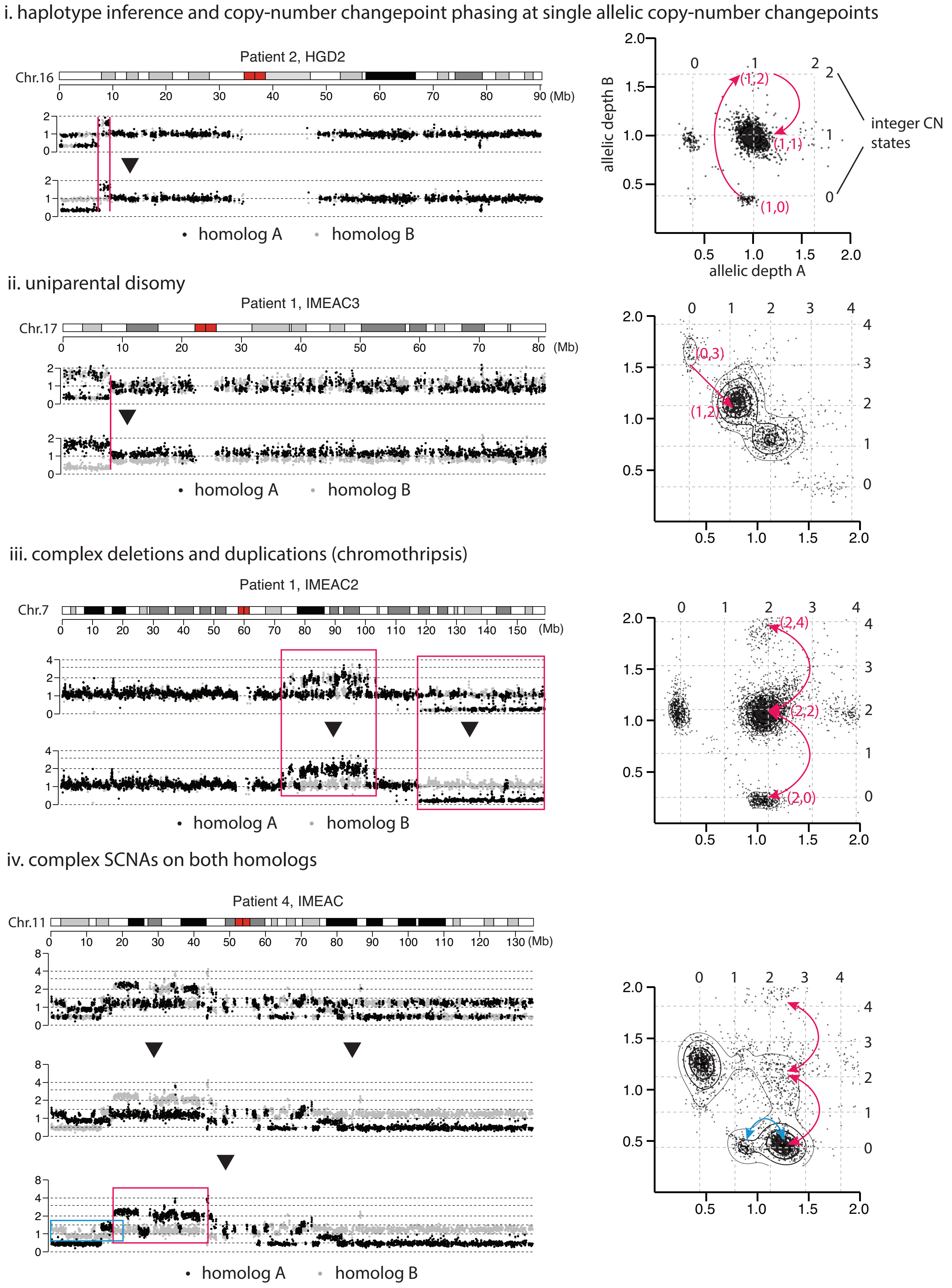

Supplementary Fig. S15. Examples of haplotype phasing by allelic imbalance in a single sample. (i) Continuous allelic imbalance with adjacent deletion and duplication on one homolog; (ii) uniparental disomy; (iii) two regions of allelic imbalance separated by a region of allelic balance; (iv) complex copy-number alterations on both homologs with different allelic depths changes. (Left) Normalized allelic depths (gray and black dots for opposite homologs) calculated from the statistical haplotype (top) and after allelic-imbalance based correction of haplotype switching (bottom); (right) scatter plots of allelic copy-number states and transitions (arrows) before haplotype correction; the integer copy-number states (after purity/ploidy correction) are annotated as gray dashed lines. Transitions (red arrows) between different copy-number states (annotated in red) on the scatter plots correspond to SCNAs (magenta lines or boxes) shown on the left. See Suppl. Fig. S14 for schematic illustrations of allelic depths and transitions. In (iii), we expect the terminal deletion and the internal duplication to occur on a single homolog in two successive BFB cycles, the first generating the terminal deletion ("chromatid type") with a small region of retention, the second generating the internal duplication ("chromosome type") with interspersed DNA losses. Regions with altered DNA copy number generated by these two events are outlined on the left. See **Main Figure 5** for further information about the outcomes of breakage-fusion-bridge cycles and Suppl. Fig. S14ii, middle panel for the incorporation of this mechanistic insight in the phasing of SCNAs. In (iv), the final haplotype phase of the p-arm is inferred based on the different changes in allelic depths: ΔCN ≈ 1.2 for the black homolog reflecting three-copy changes (red arrows on the right) and ΔCN ≈ 0.4 for the gray homolog reflecting single-copy changes (cyan arrow). Regions with oscillating copy number on each homolog are outlined on the left with matching colors. As this sample (Patient 4, IMEAC) is inferred to have undergone whole-genome duplication (WGD), alterations to the black homolog with three-copy changes most likely occurred before WGD, whereas alterations to the gray homolog with single-copy changes occurred after WGD. This example underscores the importance of haplotype-specific copy-number analysis for both phylogenetic and mechanistic inferences of SCNA evolution.

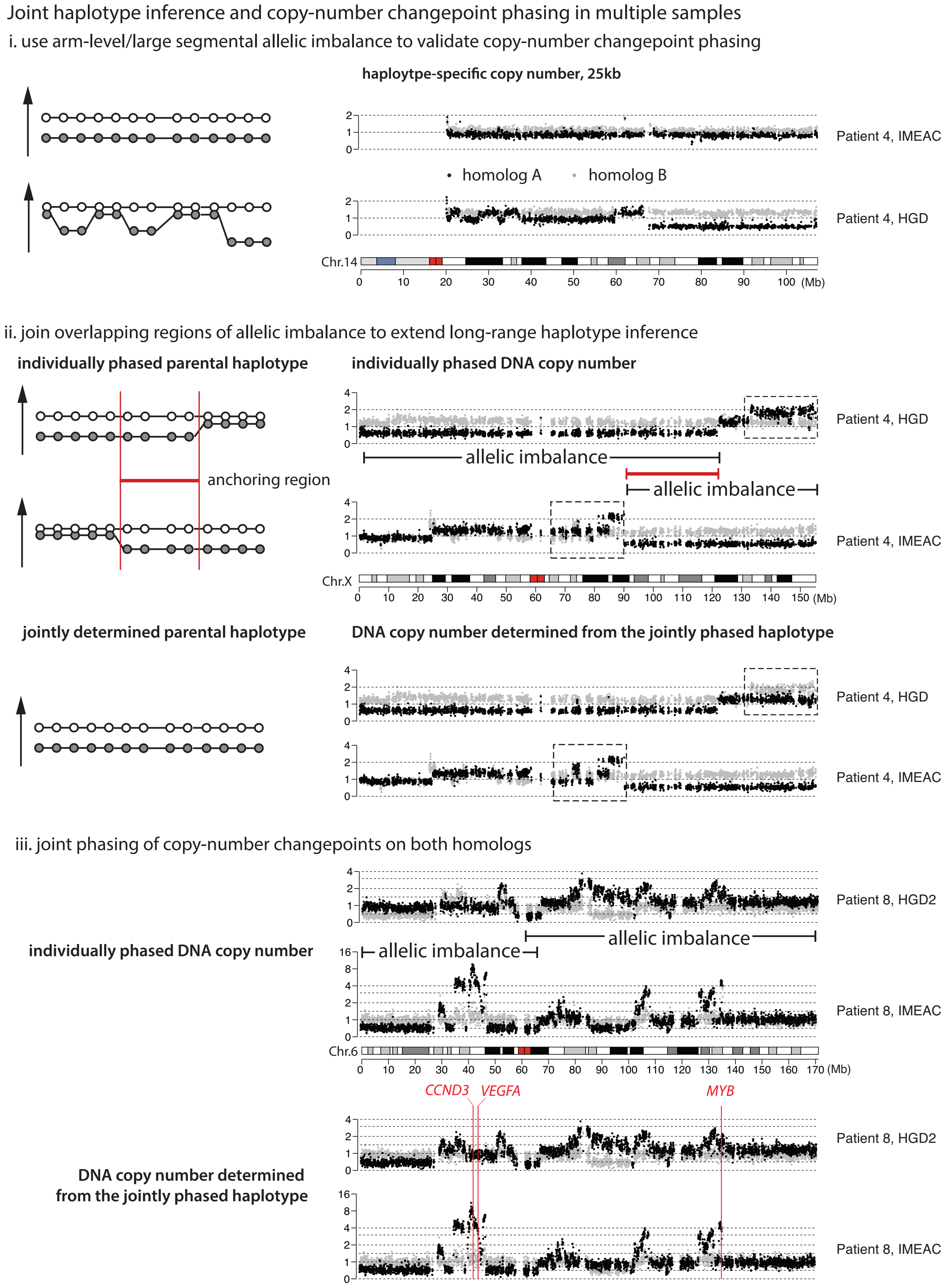

Joint haplotype inference and copy-number changepoint phasing in multiple samples
i. use arm-level/large segmental allelic imbalance to validate copy-number changepoint phasing
haploytpe-specific copy number, 25kb
Patient 4, IMEAC
homolog A
homolog B
Patient 4, HGD
Chr.14
(Mb)
ii. join overlapping regions of allelic imbalance to extend long-range haplotype inference
individually phased parental haplotype
individually phased DNA copy number
Patient 4, HGD
allelic imbalance
anchoring region
allelic imbalance
Patient 4, IMEAC
Chr.X
(Mb)
jointly determined parental haplotype
DNA copy number determined from the jointly phased haplotype
Patient 4, HGD
Patient 4, IMEAC
iii. joint phasing of copy-number changepoints on both homologs
Patient 8, HGD2
allelic imbalance
individually phased DNA copy number
allelic imbalance
Patient 8, IMEAC
Chr.6
(Mb)
CCND3
VEGFA
MYB
Patient 8, HGD2
DNA copy number determined from the jointly phased haplotype
Patient 8, IMEAC

Supplementary Fig. S16. Examples of haplotype correction from allelic imbalance in multiple samples from one individual. (i) Allelic imbalance in the IMEAC sample is used to derive the complete chromosomal haplotype that is then used to calculate/validate SCNAs in the HGD sample. (ii) Allelic imbalance in the HGD sample (0-120Mb) and in the IMEAC sample (90-qter) are joined based on the haplotype phase in the overlapping region of allelic imbalance in both samples (90-120Mb). The jointly inferred haplotype is then used to calculate chromosome-specific DNA copy number (shown on the bottom). (iii) Another example of joint haplotype inference based on overlapping allelic imbalance that resolves complex SCNAs on the black homolog and reveals a large segmental deletion (85-100Mb) of the gray homolog in HGD. There are several noteworthy observations in this example. First, the HGD1/HGD2/IMEAC samples have the lowest sequencing depths among all samples (HGD1:$5.5\times$; HGD2:$6.3\times$; IMEAC:$7.2\times$); the agreement between the 6p haplotypes that are independently derived from each of the three samples demonstrates the accuracy of our computational algorithm for allelic-imbalance based haplotype correction even when it is applied to individual samples sequenced at $5-7\times$ depth. Second, the distinct patterns of deletions and duplications/amplifications in the HGD2 and IMEAC genomes are inferred to have arisen from a single unstable ancestral chromosome based on multiple shared copy-number breakpoints including the shared boundary of the p-terminal deletion. Moreover, we inferred an ancestral WGD event preceding the divergence of these two samples. Based on the complete deletion at the 6p terminus, the ancestral unstable chr6 could have been generated prior to WGD but undergone multiple rounds of alterations both before and after WGD that create focal amplifications. Third, HGD1 and IMEAC have nearly identical copy-number breakpoints but display different copy-number states (see copy-number plots in the Online Data Repository); this pattern is consistent with complex ecDNAs arising from chromothripsis (which generates interspersed DNA deletions between the focally amplified regions) but undergoing asymmetric segregation to create copy-number heterogeneity. This inference is consistent with results from fluorescence in-situ hybridization (FISH) analysis of *VEGFA* that is located at ≈43.7Mb. We further note that the amplified DNA in the HGD1/IMEAC genomes also contains two other oncogenes, *CCND3* at 41.9Mb and *MYB* at 135.2Mb (GRCh37 coordinates), but the amplified DNA in HGD2 contained none of the oncogenes (including *VEGFA*). It is plausible that the amplified DNA was generated by an ancestral chromothripsis event but distributed into two daughter cells in a reciprocal manner; the daughter cell that inherited oncogenic DNA fragments subsequently acquired oncogenic amplifications and became the ancestor to HGD1 and IMEAC, whereas the sibling cell was the ancestor of HGD2 that did not transform to EAC.

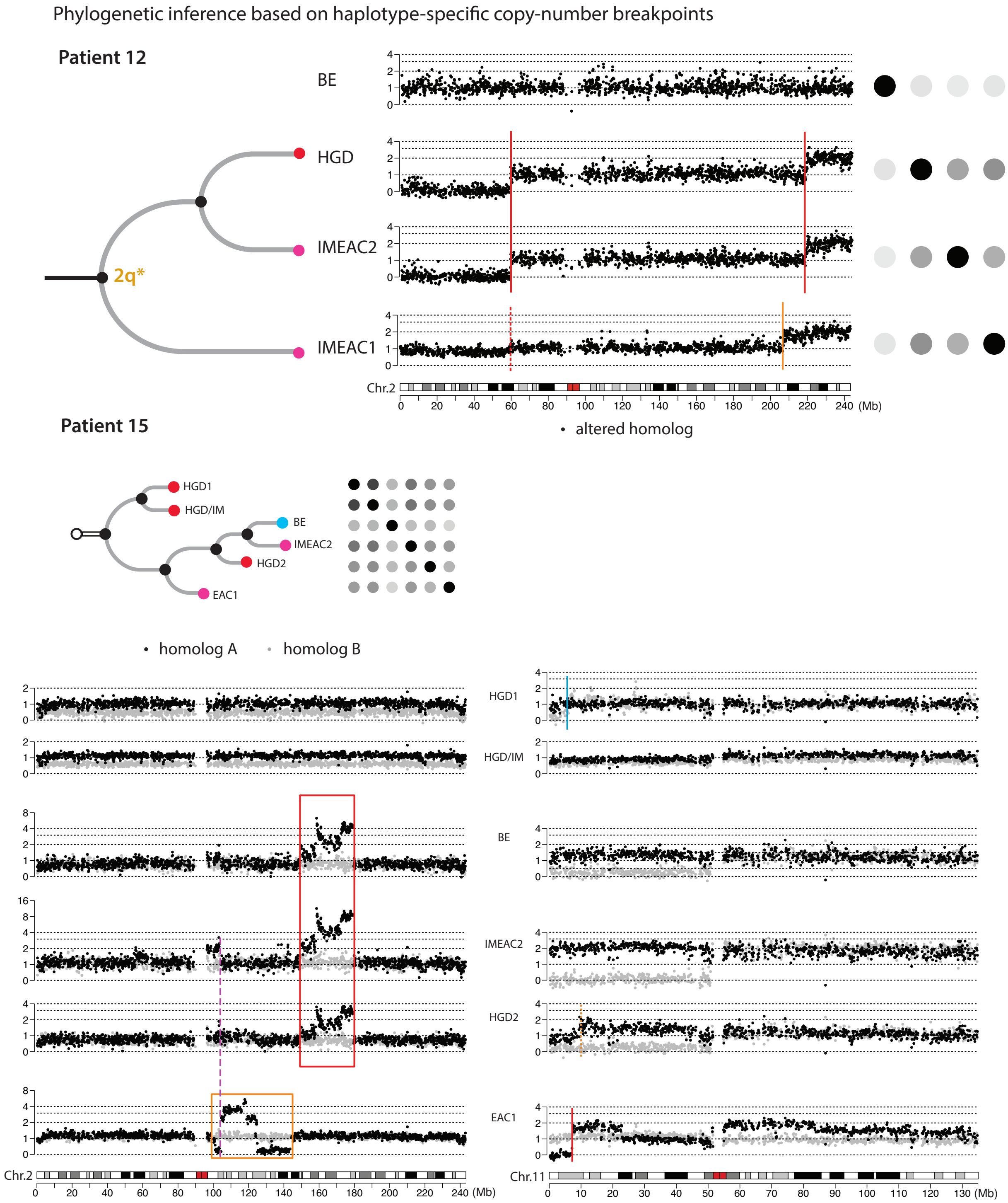

Supplementary Fig. S17. Phylogenetic inference based on haplotype-specific copy-number changepoints. (Top left) Phylogenetic relationship between HGD, IMEAC1, and IMEAC2 samples from Patient 12 derived from copy-number changepoints. (Top middle) Haplotype-specific copy number changepoints on chr2 in each genome. Note that the breakpoint at 60Mb shared between HGD and IMEAC2 is also present in a subclonal population in IMEAC1 as indicated by the minor decrease in allelic depth; also note that the breakpoint near 220Mb that is shared between HGD and IMEAC2 and establishes their kindred relationship has a related breakpoint near 208Mb in IMEAC1. (Top right) Pairwise SNV similarity estimated from the fraction of shared sSNVs between these samples. (See Suppl. Fig. S2 for the color scale.) Although the SNV similarity between HGD and IMEAC1 is slightly higher than between HGD and IMEAC2, this is most likely due to two technical reasons: (1) the lower sequencing depth of IMEAC2 (14.1×) in comparison to IMEAC1 (26.8×) causes a higher false-negative rate of variant detection in the IMEAC2 sample; (2) the presence of subclonal HGD/IMEAC2 cells in the IMEAC1 sample (as indicated by the subclonal copy-number breakpoint at 60Mb) confounds the calculation of SNV similarity. (Middle) SCNA-derived phylogenetic tree and pairwise sSNV similarity of samples from Patient 15. (Bottom) Evidential support for the SCNA-based phylogenetic inference. The complex SCNA patterns on chr2q (black homolog) and 11p loss (gray homolog) shared between BE, IMEAC2, and HGD2 establish their kindred relationship; the shared breakpoint (magenta dashed line) with complementary copy-number changes in IMEAC2 and EAC1 indicates a reciprocal distribution of broken fragments of an ancestral chromosome. The absence of any SCNA breakpoint on the black homolog of chr2 in HGD1 and HGD/IM suggests that they descended from a distinct ancestor from the common ancestor of the other four samples. Note the unrelated 11p-terminal deletion in HGD1 on the gray homolog and the terminal deletion and duplication in EAC1 on the black homolog that will confound phylogenetic inference from unphased SCNA breakpoints. The apparently high sSNV similarity between HGD1, HGD/IM and IMEAC2 is most likely the outcome of false negative variant detection in BE, HGD2, and IMEAC2 genomes due to the low clonal fractions of tumor cells (BE:28%; HGD2:27%; IMEAC2:24%).

Patient 7, HGD2. Purity: 60%; ploidy: 1.91 | Allelic depths in 25kb intervals calculated from down-sampled allelic coverage on Chr.18 with mean sequencing depths of 30×, 20×, 10×, 5×.

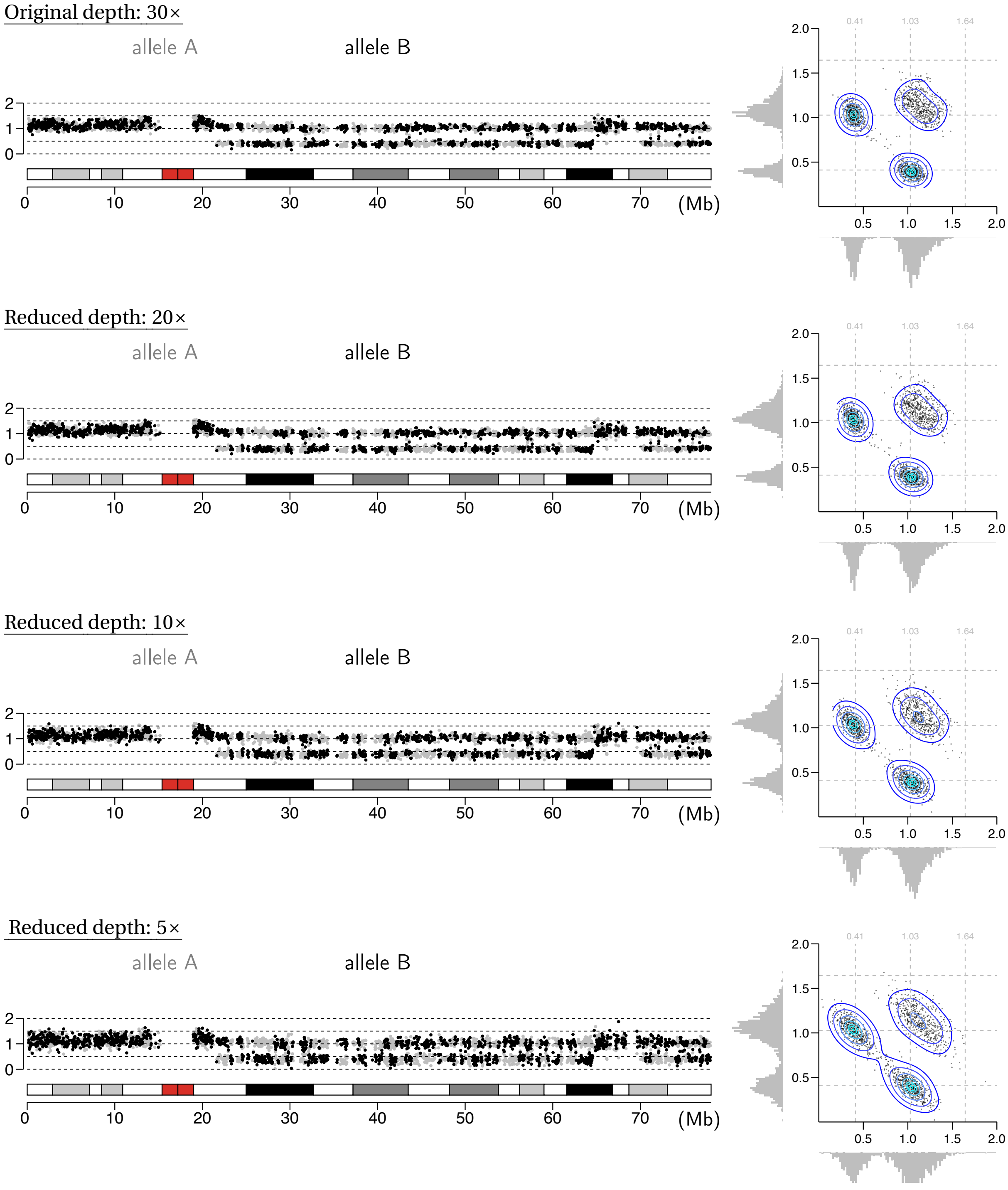

Supplementary Fig. S18. Allelic depths in 25kb intervals calculated from the original (30×) and down-sampled allelic coverage of Chr.18 in sample HGD2 from Patient 7. Left panels show the allelic depths along Chr.18: Black and gray dots represent the normalized depth of each allele ("allele A" and "allele B"). Right panels show the scatter plots and distributions of allelic copy-number states: X- and Y-axis represent allele A and allele B depths; dotted gray lines represent allelic depths (annotated with numbers in gray) corresponding to integer copy-number states $0, 1, 2, \ldots$; the contour map represent the 2D distribution of allelic copy-number states, the histograms on each axis represent the 1D distribution of allelic copy-number states. The reduced allelic depth on 18q is inferred to be a clonal deletion (tumor purity 60%). The separation between different allelic copy-number states (in both 2D and 1D distributions) ensures the accuracy of long-range haplotype phasing based on allelic depth differences. There is a minor increase in the statistical variations of allelic depths when the sequencing depth is reduced from 20× to 10× and a further increase as when reduced to 5×. This analysis suggests that this deletion can be reliably identified and phased even at 5× mean sequencing depth.

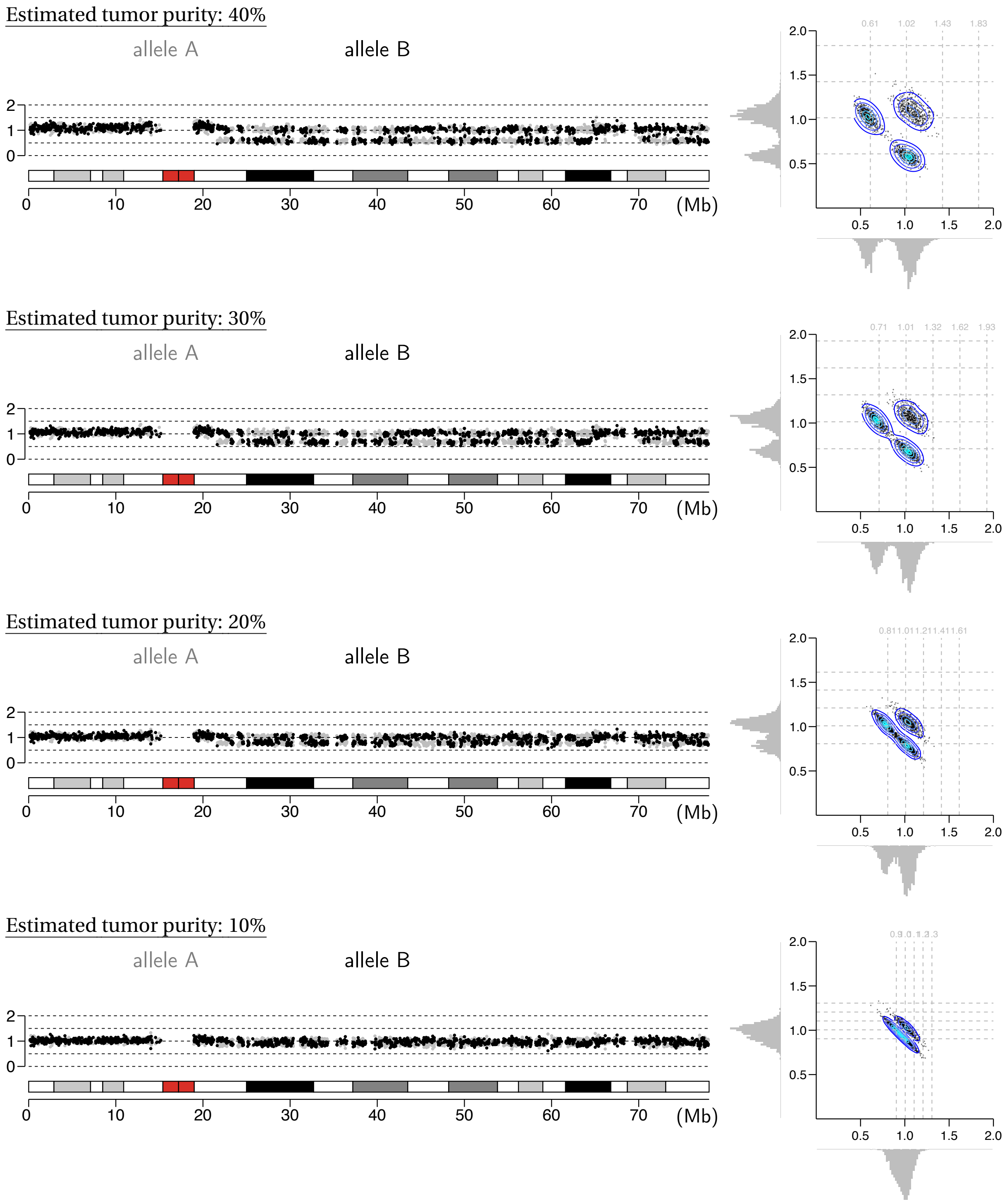
Estimated tumor purity: 40%
allele A
allele B
0.61
1.02
1.43
1.83
0
10
20
30
40
50
60
70
(Mb)
Estimated tumor purity: 30%
allele A
allele B
0.71
1.01
1.32
1.62
1.93
(Mb)
Estimated tumor purity: 20%
allele A
allele B
(Mb)
Estimated tumor purity: 10%
allele A
allele B
(Mb)

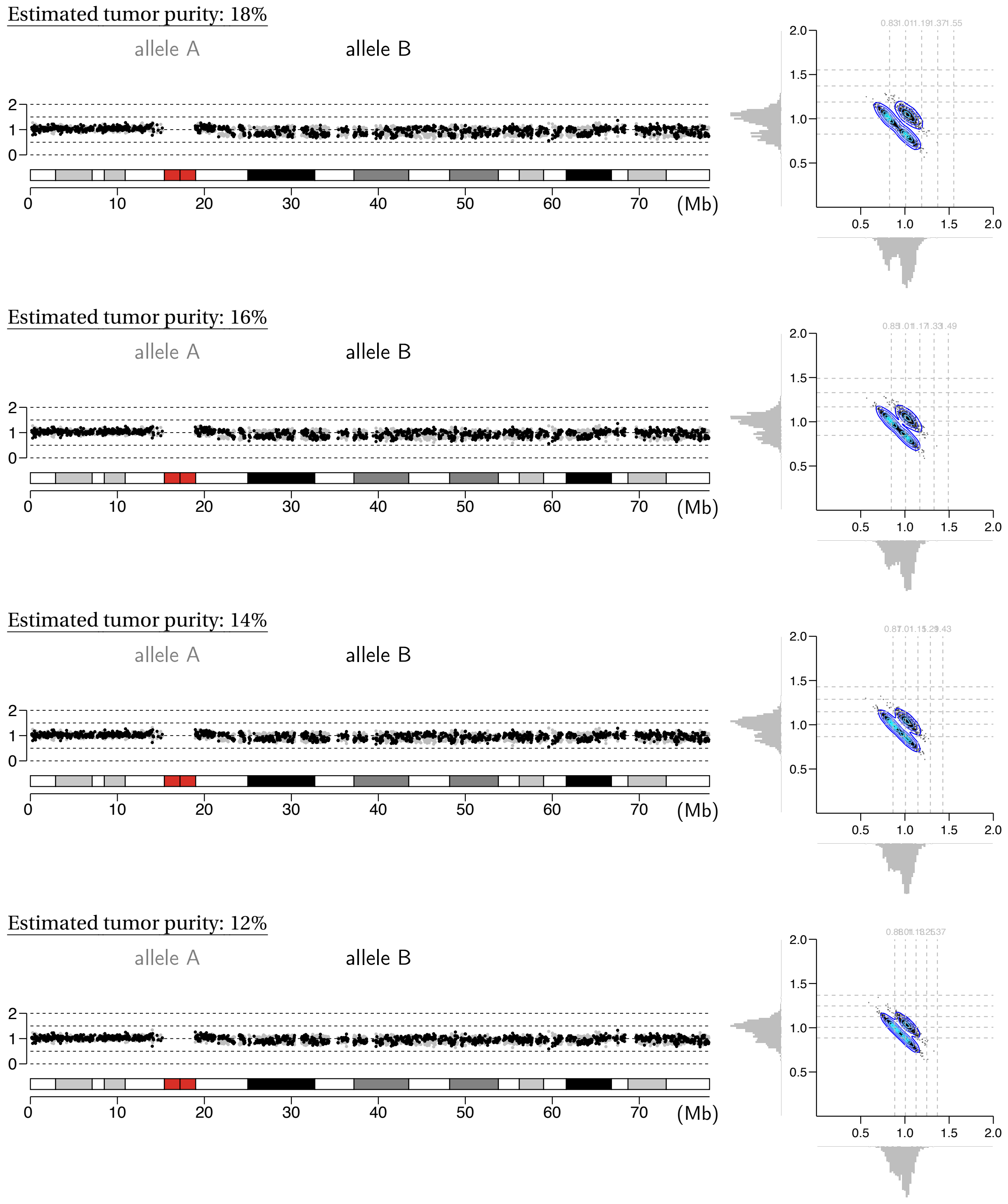

Supplementary Fig. S19. Allelic depths calculated from the allelic coverage of *in silico* mixtures of tumor and matching germline samples. The combined sequencing depth is $20\times$ and the estimated tumor cell fractions in the mixtures are 40%, 30%, 20%, 10%, 18%,16%,14%,12%. Left and right panels are the same as in Suppl. Fig. S18. Based on the allelic depth distributions, the lowest clonality of single-copy changes that can be resolved from allelic depth differences is 18-20%.

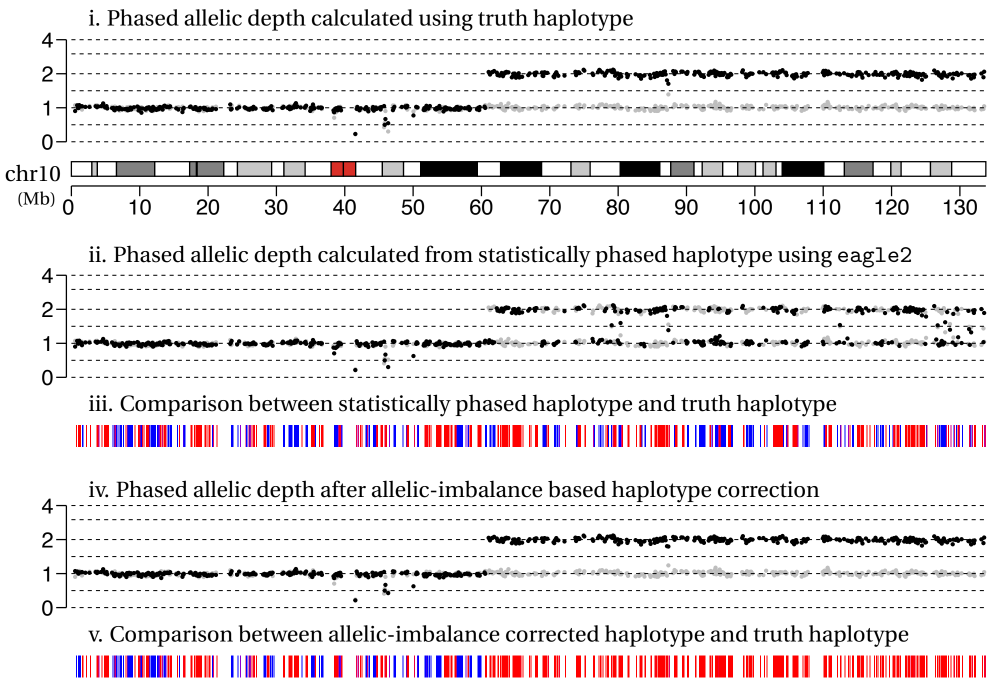

Supplementary Fig. S20. An example of haplotype inference from a single region with contiguous allelic imbalance. Black and gray dots correspond to 100kb allelic depths calculated using the complete chromosomal haplotype phase determined from long-range sequencing (top, i), statistical haplotype (middle, ii), and allelic-imbalance corrected haplotype (bottom, iv). Note that black and gray dots are arbitrarily assigned to haplotype A and B in the truth data (i) and in the statistical phasing results (ii and iii) as they are calculated independently. When comparing the statistical haplotype or the allelic-imbalance corrected haplotype against the truth haplotype data, we use red and blue bars to denote agreement with complementary parental haplotypes: Oscillations between blue and red indicate haplotype switching; consecutive blue or red indicate consistency with a single parental haplotype. The example here showed that our algorithm correctly resolves the long-range haplotype in the region of allelic imbalance (from 61Mb to the q-terminus) by eliminating switching errors (oscillation between blue and red) in the statistical haplotype. Note that the long-range haplotype inference is restricted to the region with allelic imbalance and leaves out the region in allelic balance (p-terminus to 61Mb).

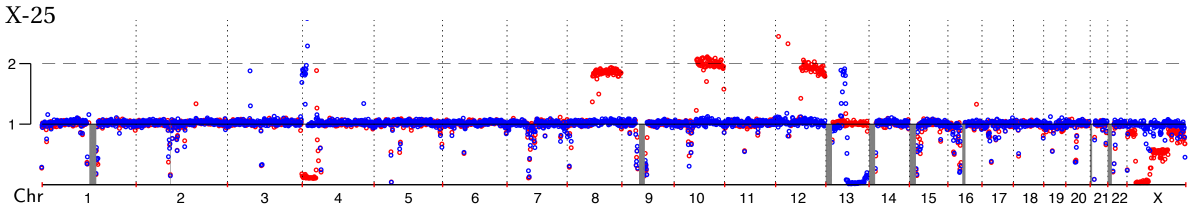

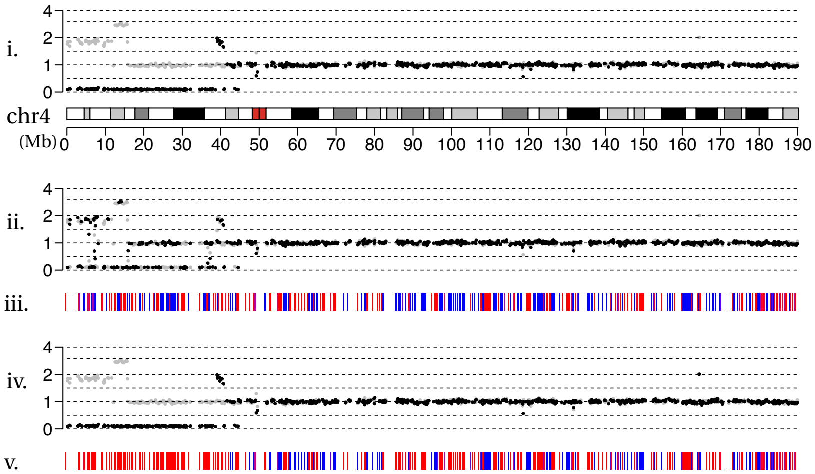

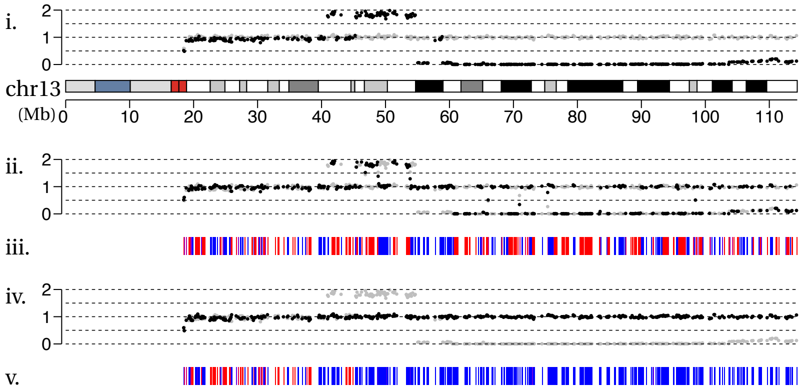

Supplementary Fig. S21. Examples of haplotype inference based on changes in the allelic depths of both homologs (Chr.4) and multiple regions of allelic imbalance on a single homolog (Chr.13). Both examples are from the X-25 clone after telomere crisis in Maciejowski et al. (2015)[8]. The top panel shows genome-wide DNA copy number of both homologs (red and blue) that are calculated using the truth haplotype. The lower panels show results of allelic-imbalance based haplotype correction. Both examples validate the correct haplotype inference in regions of allelic imbalance and further demonstrate the capability to resolve complex segmental alterations. For Chr.4p, the constraint that each allelic depth changepoint only affects one homolog (Suppl. Fig. S14i) is sufficient to determine the 4p haplotype despite SCNAs affecting both homologs. For Chr.13q, the phasing of interspersed segmental gains and the terminal deletion to a single homolog reflects the assumption that segmental copy-number changes more often arise on a single homolog (Suppl. Fig.S14ii, middle) and is also consistent with the outcomes of sequential breakage-fusion-bridge cycles (see Suppl. Fig. S15iii and its caption); moreover, the phasing of both alterations to a single homolog is directly verified by the truth haplotype data. Note that switching errors within the region of duplication (red bars) are restricted to interspersed regions of allelic balance, which do not impact the phasing and interpretation of SCNAs.

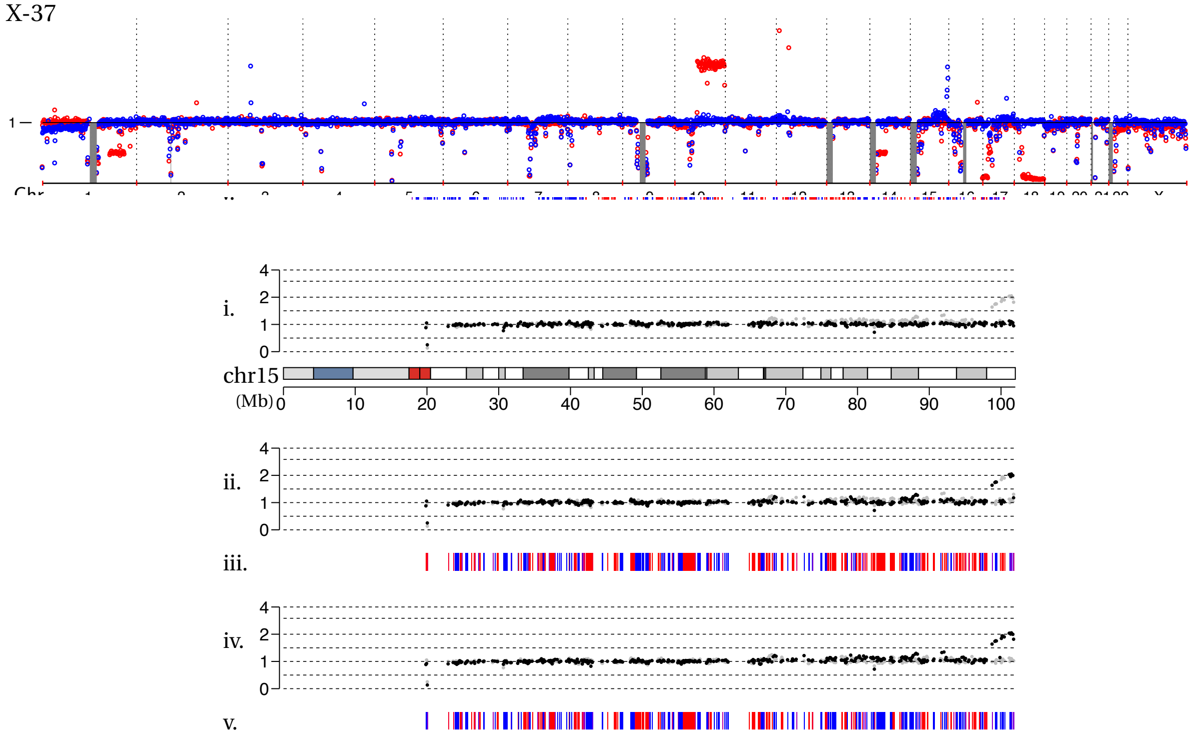

copy-number homolog) and near the 6q terminus.

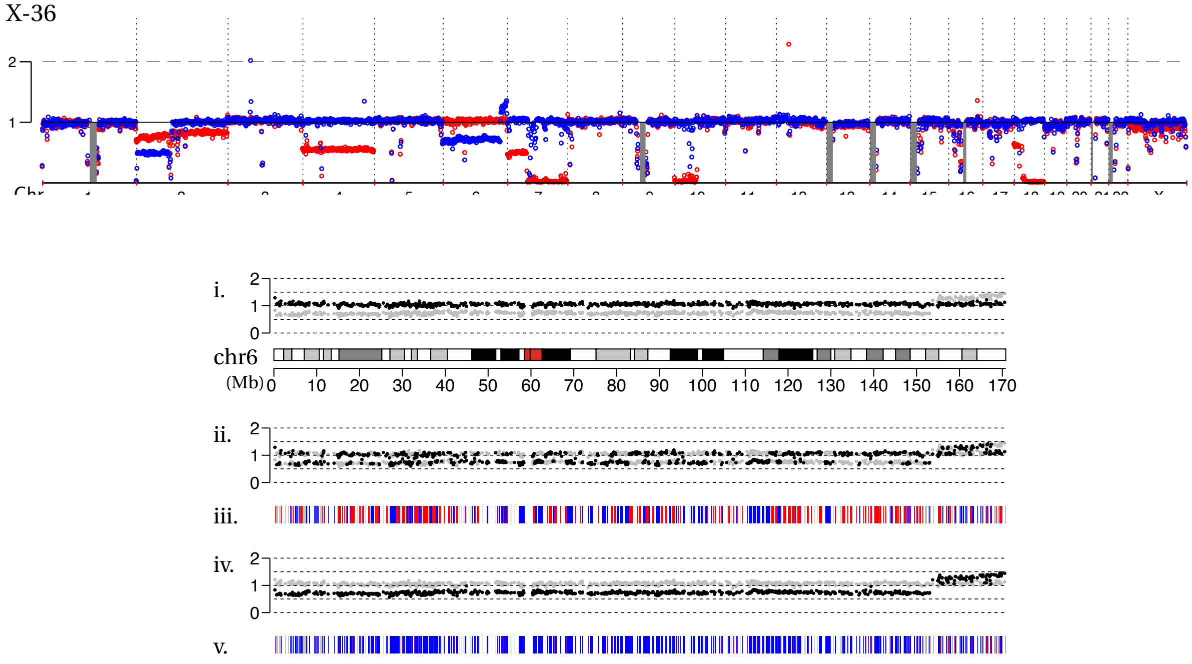

Supplementary Fig. S22. Examples of sloping copy number variation on a single haplotype in two post-crisis RPE-1 clones X-37 (Chr.15) and X-36 (Chr.6). The plots are the same as in Suppl. Fig.S21. Note the correct phasing of sloping copy number on one homolog and constant copy number on the opposite homolog. This result not only validates the correct haplotype inference based on allelic imbalance but provides further evidence that telomere crisis or breakage-fusion-bridge cycles can generate sloping copy-number variation.
