# Supplementary material for: Genomic signatures of past and present chromosomal instability in Barrett’s esophagus and early esophageal adenocarcinoma
Source: Nat Commun. 2023 Oct 4;14:6203. doi: 10.1038/s41467-023-41805-6 (PMC10550953; doi:10.1038/s41467-023-41805-6)
Supplement: Supplementary file 3 — Description of Additional Supplementary Files [file 41467_2023_41805_MOESM3_ESM.pdf]

Description of Additional Supplementary Files

Supplementary Data S1 (Sample.Summary.xlsx)

Sample information and metrics of sequencing data. Clinical information (Columns A-C), mean sequencing depth (Column D), inferred clonal fraction (Column E), average ploidy (Column F), and WGD status (Column G) of the dominant tumor clone, basic metrics of sequencing data (Columns H-L), allelic sequence coverage (Columns M-R). Samples without identifiable SCNAs (> 0.1 Mb) are assigned as diploid with purity not assigned. Column M shows the number of heterozygous sites jointly detected from all samples (including both BE/EAC and normal reference) from each individual. Column N shows the number of heterozygous sites with sequence coverage in each sample. Reference and alternate genotype coverage (Columns O and P) and standard deviation (Columns Q and R) are calculated using heterozygous variant sites in each individual but excluding those with allelic depth ≥ 100.

Supplementary Data S2 (All.SCNA.xlsx)

List of somatic copy-number alterations (SCNA) in individual samples (Tab 1) and individual evolutionary branches (Tab 2). In Tab 1, Patient_ID (Column A) and Sample_ID (Column B) are according to Supplementary Data S1; Column C: unique ID for each SCNA; Columns D-F denote the altered chromosome and its parental haplotype; Column G: SCNA classification based on Supplementary Figure S4 (1: focal deletion/duplication; 2: uniparental disomy; 3; arm-level SCNAs; 4: terminal SCNAs; 5: paracentric SCNAs; 6: pericentric SCNAs; 7: complex deletions/losses; 8: complex duplications/gains; 9: focal amplifications.); Column H: SCNA outcome (LOH: loss-of-heterozygosity through uniparental disomy; deletion: allelic DNA copy number = 0; loss: allelic DNA copy number < basal copy-number state; gain: allelic DNA copy number > basal copy-number state; amplification: allelic DNA copy number at or above 8); Column I: maximum allelic copy-number state; Column J: additional features of complex SCNAs. In Tab 2, Branch_IDs (Column A) are according to annotations in Supplementary Figure S2; Column B: number of progeny clones; Column C: relative timing to p53 loss (0 for prior to p53 loss, 1 for after p53 loss); Column D: relative timing to WGD (0 for pre-WGD branches, including all branches in patients without WGD; 1 for branches with WGD acquisition; 2 for post-WGD branches); Column E: number of SCNAs assigned to each branch, with individual SCNAs (annotated by unique IDs according to Column C of Tab 1) listed in Column F. Tab 3 contains a summary of the total number of SCNAs of each class grouped by their relative timing to WGD. Tab 4 contains a summary of the number of SCNAs of each class along different evolutionary branches grouped by their relative timing to WGD.

Supplementary Data S3 (SCNAs.in.single.BE.cells.xlsx)

Summary of genetic alterations in single BE cells related to **Main Figure 3**. Tab 1: *TP53* mutation status (Column B) with genotype likelihoods (Column C) calculated by HaplotypeCaller. Tab 2-7 list single cells from different clones/groups and their shared or private SCNAs. Aneuploid cells (Tab 7) are separated into two groups (near diploid and near tetraploid) with allelic copy-number states of each homolog annotated individually. Chromosome-level copy-number plots of every cell are available at https://zenodo.org/record/8265676.

Supplementary Data S4 (LocalSCNAsandUPDs.xlsx)

List of simple (focal) SCNAs (Tab 1) and uniparental disomy alterations (Tab 2) detected in bulk BE and EAC samples. Each row represents a SCNA that is annotated for the region of alteration (Column B), the altered haplotype (Column C) according to copy-number plots (available in the Online Data Repository), copy number outcome (Column D), affected samples (Column E), phylogenetic pattern (Column F), relative timing to *TP53* inactivation (Column G), and relative timing to WGD (Column H). We further added events inferred to have occurred prior to UPDs in column I of Tab 2.

Supplementary Data S5 (Terminal.Internal.SCNAs.xlsx)

List of SCNAs consistent with outcomes of single (Tab 1) or multigenerational (Tab 2) BFB cycles. Columns A-C, E-G are similar to Supplementary Data S4. For single-generation BFB outcomes (Tab 1), Column D shows the copy-number classification; for multigenerational outcomes (Tabs 2), Column D shows the inferred evolutionary sequence. Relative timing to *TP53* inactivation is omitted as most of these events are inferred to have occurred after *TP53* inactivation. Additional remarks are put in Column H.

Supplementary Data S6 (Evolution.Examples.xlsx)

List of SCNAs consistent with branching copy-number outcomes of BFB cycles. Tab 1: Progressive DNA losses identified in different BE/EAC genomes. Tab 2: Distinct SCNAs on the same parental homolog identified in different BE/EAC genomes from the same patient. Tab 3: SCNAs showing complementary DNA retention and deletion. Annotations are similar to Supplementary Data S5, except for the addition of the span of SCNA in Column D of Tab 2 and Tab 3.

Supplementary Data S7 (Chromothripsis.and.Focal.Amp.xlsx)

List of chromothripsis (Tab 1) and focal amplifications (Tab 2) identified in bulk BE/EAC samples; results of fluorescence in-situ hybridization analysis of amplified DNA are listed in Tab 3. In Tab 1, the annotations are similar to Supplementary Data S5, with additional annotations of the span of chromothripsis (Column G), status of telomere loss (Column H), chromothripsis subclassification (Column I), and copy-number feature (Column J). The evolutionary sequence (column K), including preceding (Column L) and downstream (Column M) alterations relative to chromothripsis, is inferred based on the copy-number features. The mechanistic inference related to bridge resolution (Column N) and micronucleation (Column O) is made based on the span and copy-number feature of chromothripsis. In Tab 2, additional annotations include potential oncogenes (Column I), EAC oncogene (Column J), and co-localization with recurrent amplifications in cancers (Column K) of each amplified region.

Supplementary Data S8 (Longitudinal.BE.Re-analysis.xlsx)

Re-analysis of longitudinal BE/EAC sequencing data. Tab 1: List of ancestral (present in all samples, Column F), shared (present in two or more samples, Column G), and private (Column H) SCNAs identified in each sample. Risk classification from the original study is kept in Columns I-K. Tab 2: Patient-level summary of SCNAs detected in all longitudinal samples from each individual. Focal/segmental losses/gains are annotated as '1p-' or '1p+', gain or loss of entire chromosome arms are annotated as '+1p', '+8q', bi-allelic/overlapping deletions are annotated as '9p--'.

Supplementary Data S9 (DownSamplePlots.pdf)

Validation of allelic depth coverage calculation from low-pass whole-genome sequencing

Supplementary Data S10 (AllelicDepthPhasing.pdf)

Validation of allelic-imbalanced based haplotype inference.
