# Supplementary material for: Genomic signatures of past and present chromosomal instability in Barrett’s esophagus and early esophageal adenocarcinoma
Source: Nat Commun. 2023 Oct 4;14:6203. doi: 10.1038/s41467-023-41805-6 (PMC10550953; doi:10.1038/s41467-023-41805-6)
Supplement: Supplementary file 4 — Supplementary Data 1-10 [file 41467_2023_41805_MOESM4_ESM.zip › Supplementary.Data/Supplementary.Data.10.AllelicDepthPhasing.pdf]

Allelic-imbalance based haplotype inference in post-crisis RPE-1 clones

Genome-wide allelic copy number calculated from truth haplotype data . . . 1

Allelic imbalance shared by all post-crisis clones: Chr.10q and Chr.12p . . . 3

X-29: Chr.11q . . . 4

X-32: Chr.18 . . . 5

24-141: Chr.7q and Chr.12q . . . 6

24-144: Chr.3q and Chr.6p . . . 7

X-33: Chrs.1q,8,18,X . . . 8

I-dox-1: Chrs.2,7,8,11,15 . . . 10

X-25: Chrs.4p,8q,12q,13q,X . . . 12

X-37: Chrs.1,14,15,17,18,22 . . . 14

X-36: Chrs.2,4,6,7,10,18 . . . 16

X-35: Chrs.1,5,6,8,10,12,16,18 . . . 18

Genome-wide allelic copy number plots of all post-crisis RPE-1 clones (1Mb) | Blue and red represent DNA copy-number (1Mb intervals) calculated using the parental haplotype of RPE-1 cells determined from long-range sequencing. Note the clonal 10q gain of the red haplotype. These clones also contain two extra copies of Chr.12p in an isochromosome (red haplotype); the gain on Chr.12p is not shown as the Y-axis is capped at allelic copy number state 2.

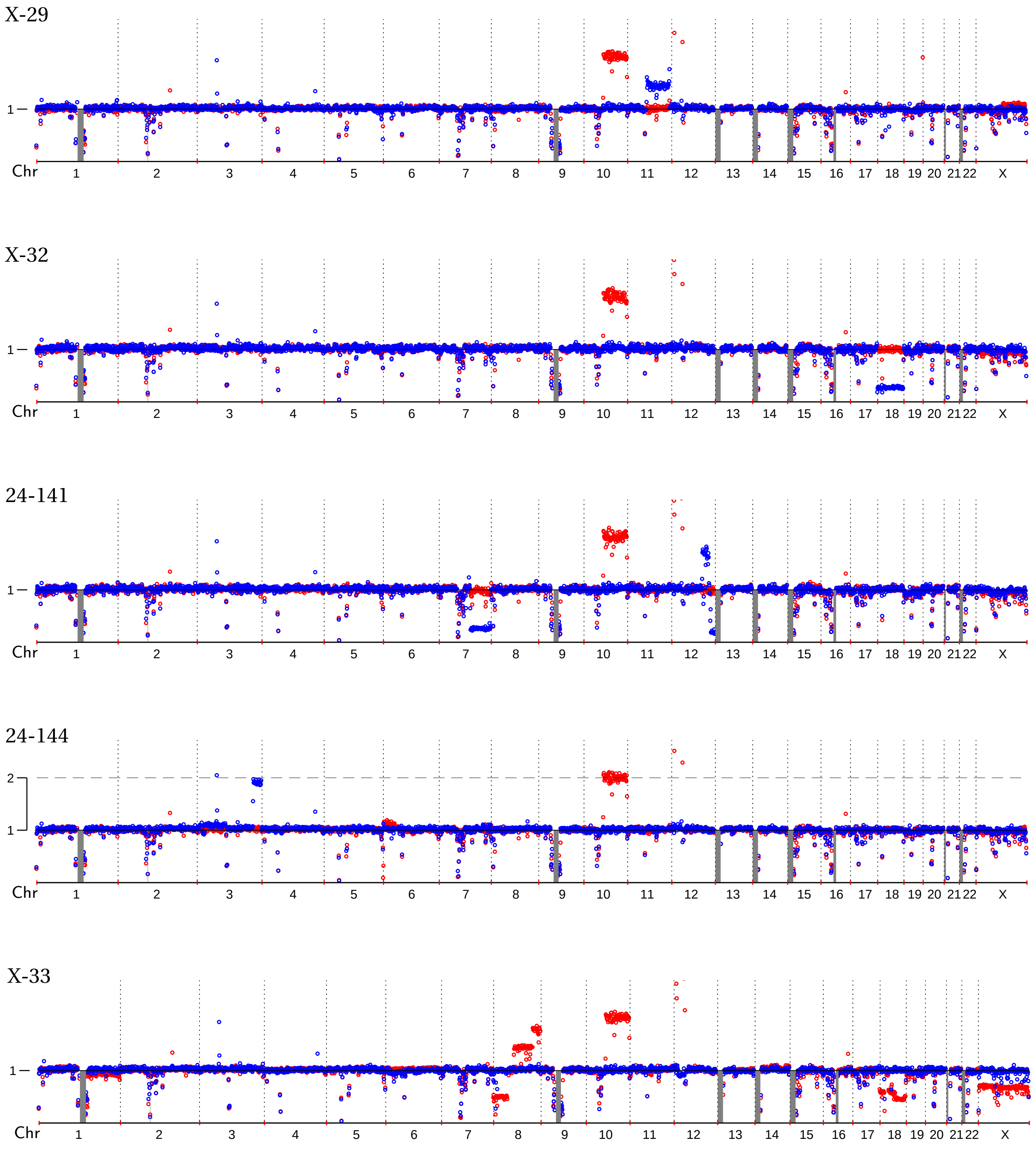

I-dox-1

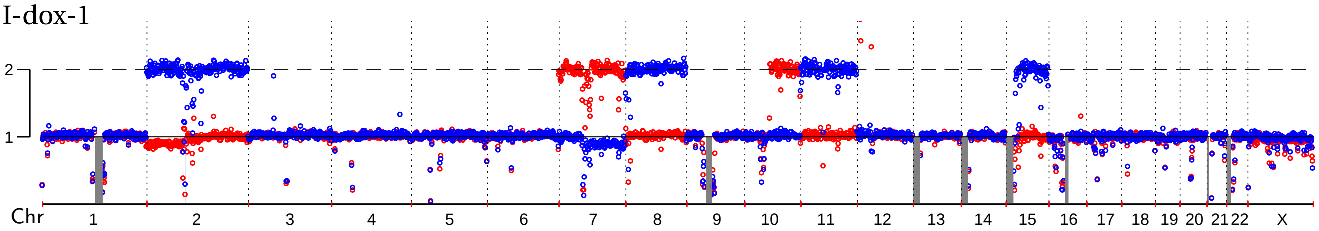

X-25

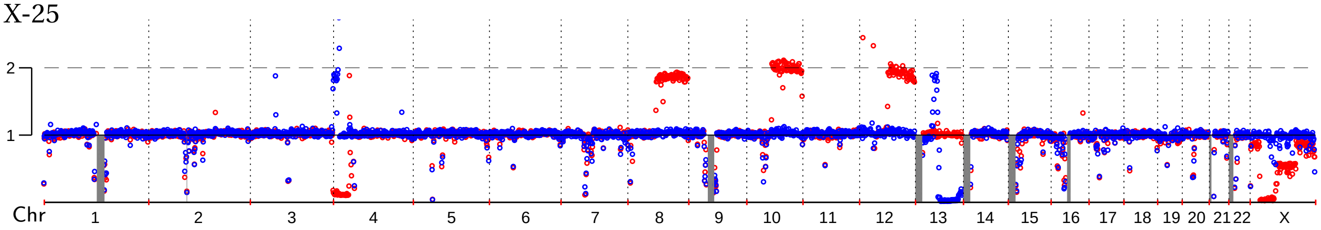

X-37

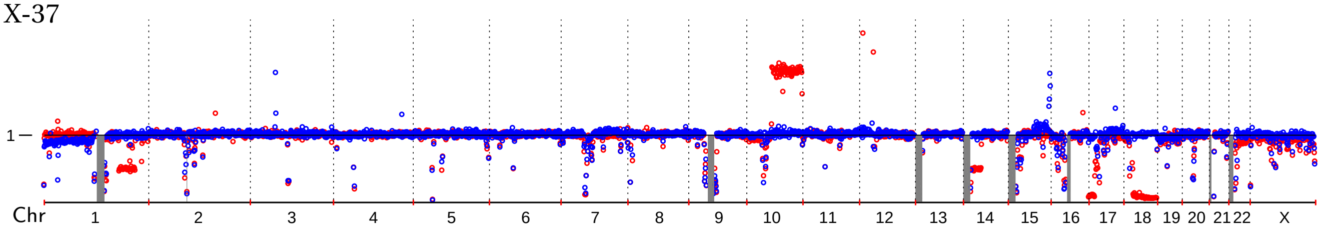

X-36

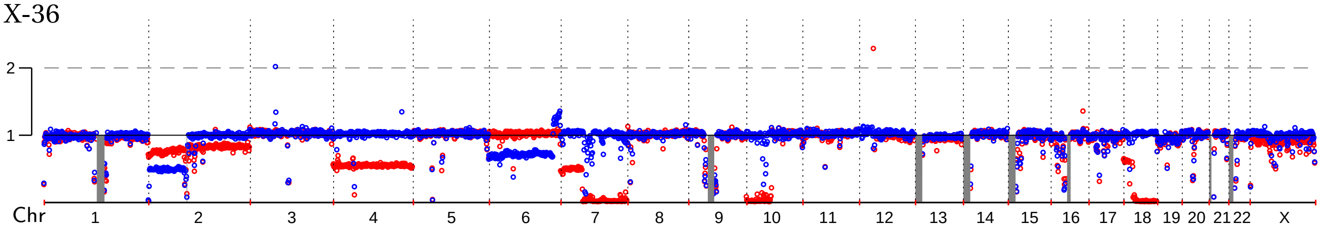

X-35

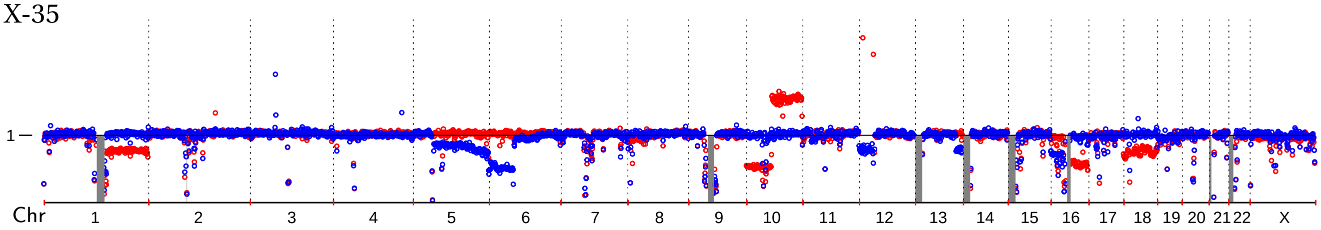

Allelic imbalance in the parental clone shared between all post-crisis clones | Allelic-imbalance based phasing of Chr.10q and Chr.12p haplotypes using reads from the X-29 clone. Black and gray dots correspond to 100kb allelic depths calculated using the complete chromosomal haplotype phase determined from long-range sequencing (top, i), statistical haplotype (middle, ii), and allelic-imbalance corrected haplotype (bottom, iv). Note that black and gray dots are arbitrarily assigned to haplotype A and B in the truth data (i) and in the statistical phasing results (ii and iii) as they are calculated independently. When comparing the statistical haplotype or the allelic-imbalance corrected haplotype against the truth haplotype data, we use red and blue bars to denote agreement with complementary parental haplotypes: Oscillations between blue and red indicate haplotype switching; consecutive blue or red indicate consistency with a single parental haplotype.

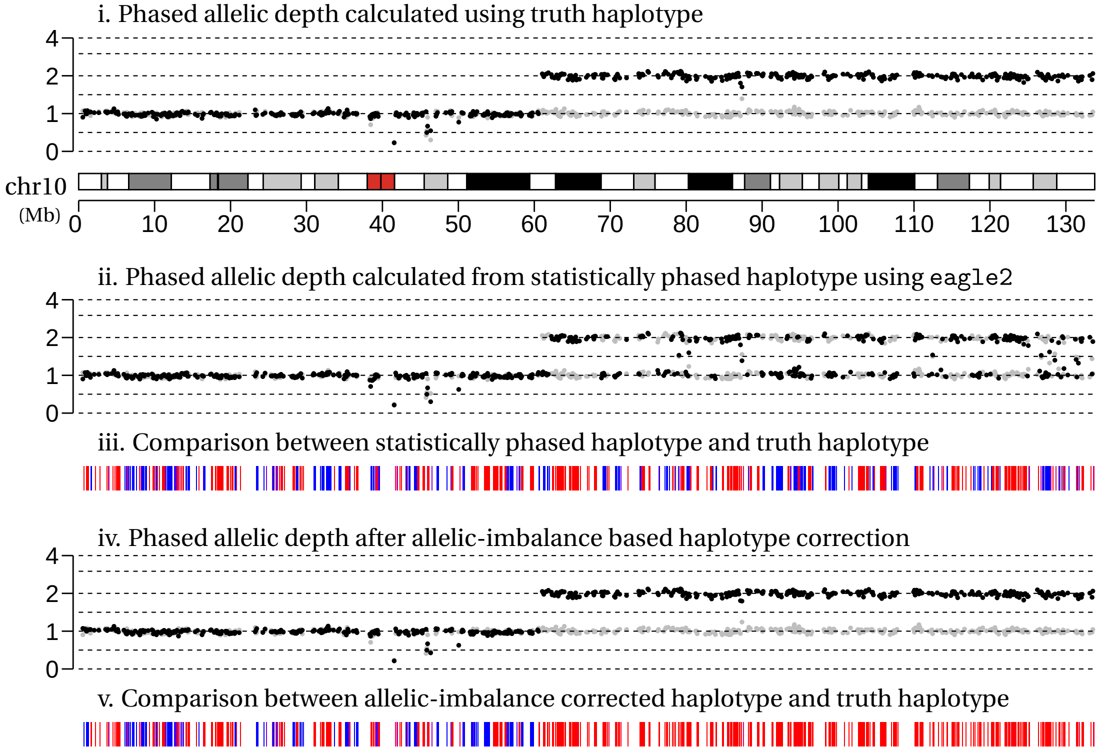

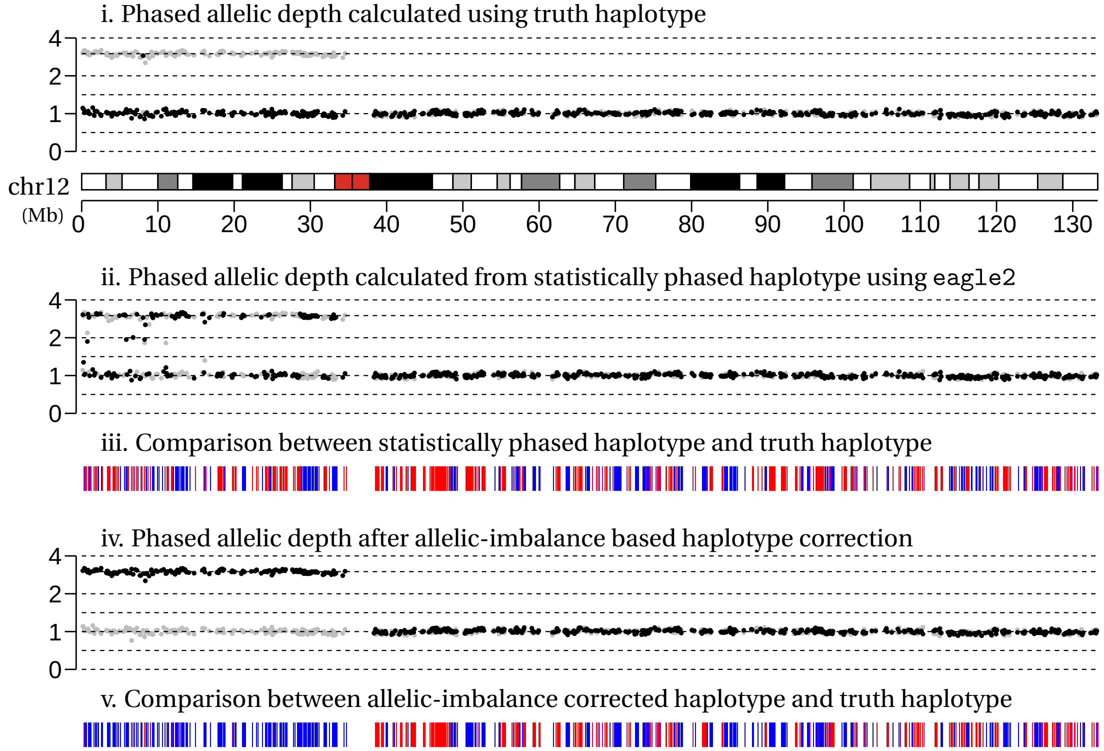

X-29 | Allelic-imbalance based haplotype phasing on Chr.11q

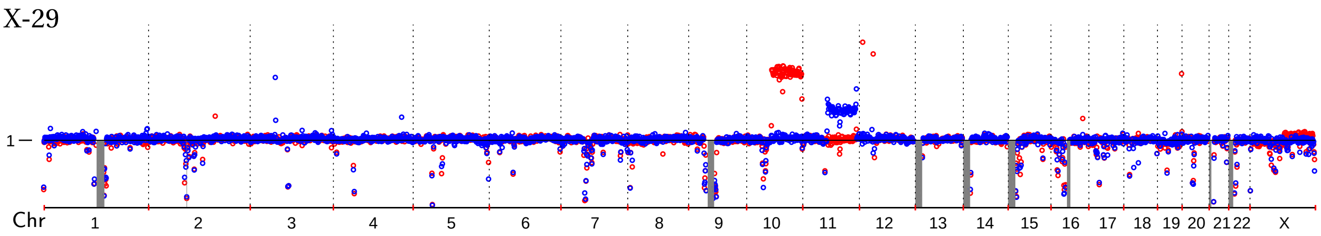

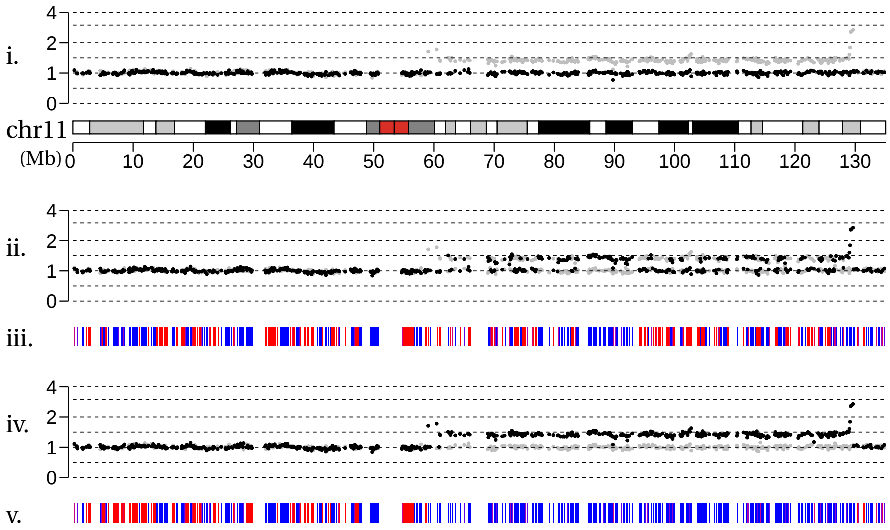

X-32 | Allelic-imbalance based haplotype phasing on Chr.18. There is one switching error at the centromere that is due to inaccuracies in the allelic depths in the pericentric region. Such errors can be easily recognized and corrected manually.

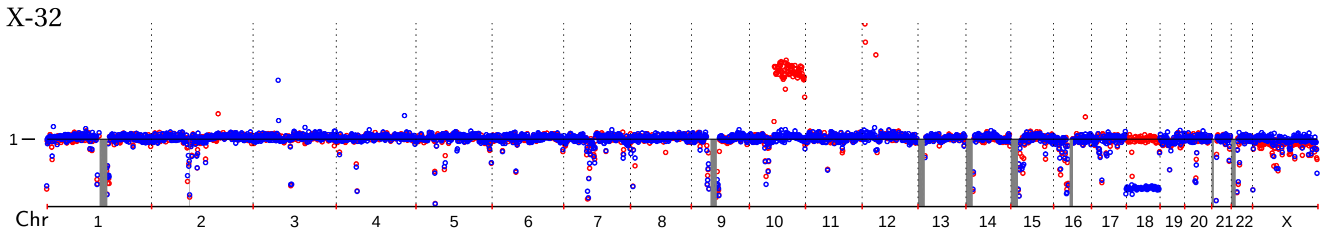

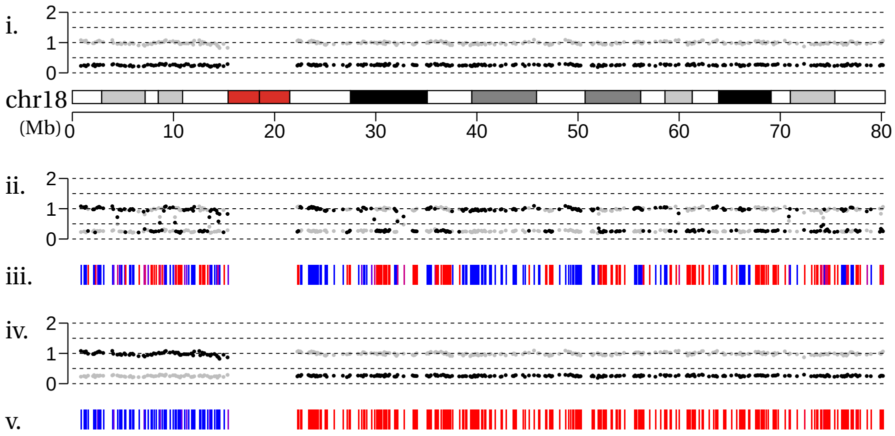

24-141 | Allelic-imbalance based haplotype phasing on Chr.7q and Chr.12q. Note that the gains on 12p is from an isochromosome and unrelated to alterations at the 12q terminus; it is impossible to determine the haplotype relationship between alterations on 12p and 12q.

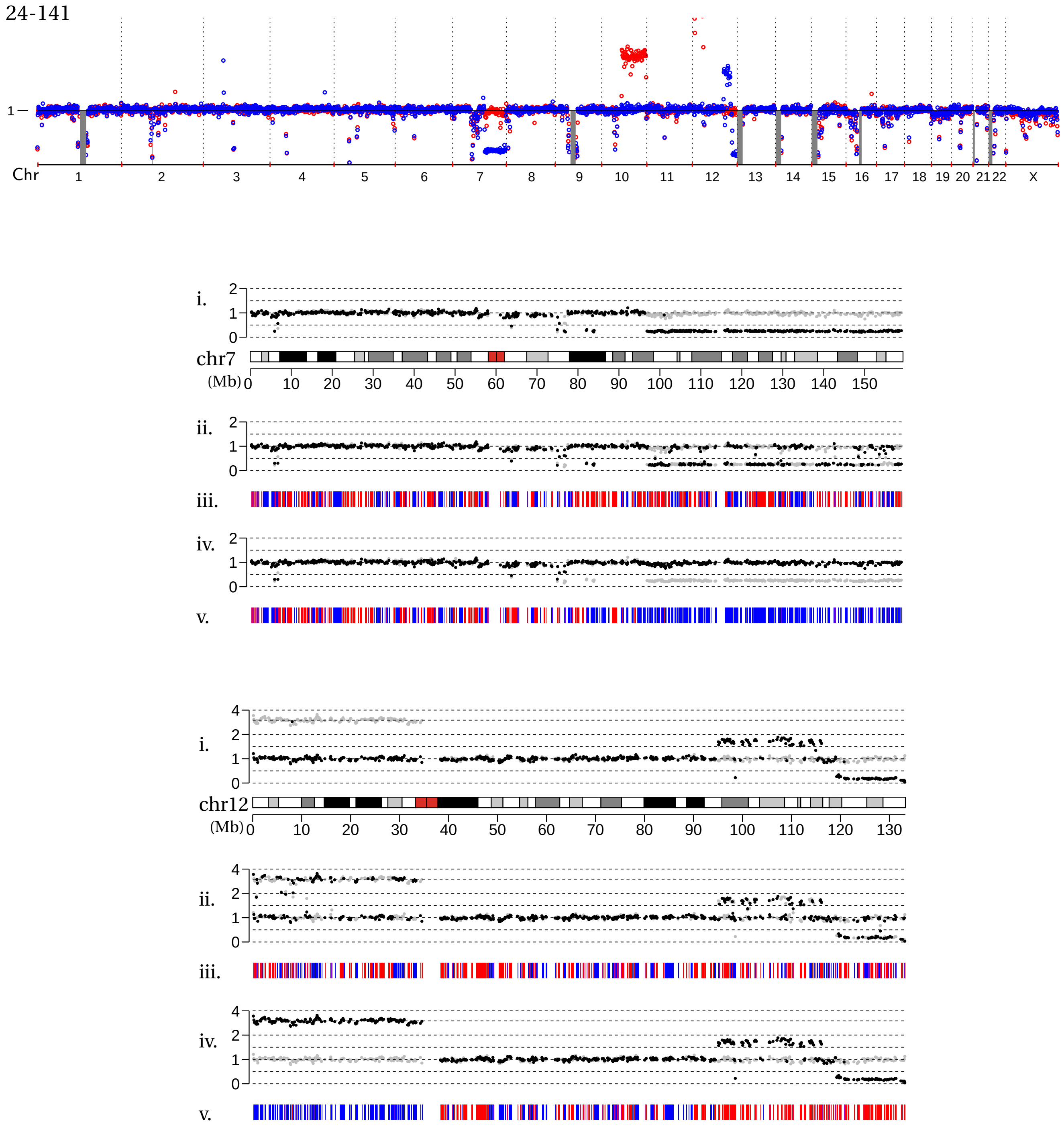

24-144 | Allelic-imbalance based haplotype phasing on Chr.3q and Chr.6p. Note that allelic-imbalance based phasing can only resolve the deletion at the 6p terminus but not the gain of 6p that is present at a low clonal fraction (≈ 0.1). The subclonal 6p gain can be identified from the haplotype-specific copy number determined from the truth haplotype. Similarly, the haplotype-specific copy number data suggest the presence of an internal deletion and duplications of the flanking segments on Chr.3p in a minor subclone; these alterations cannot be resolved by allelic-imbalance based phasing due to their low clonal fraction.

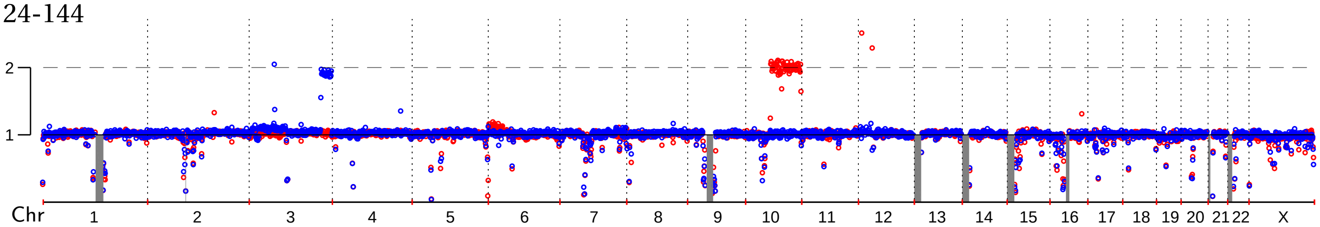

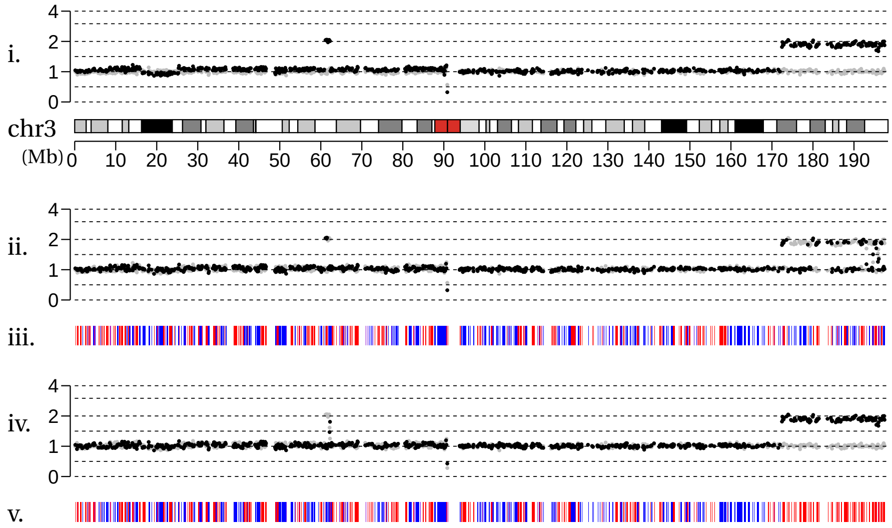

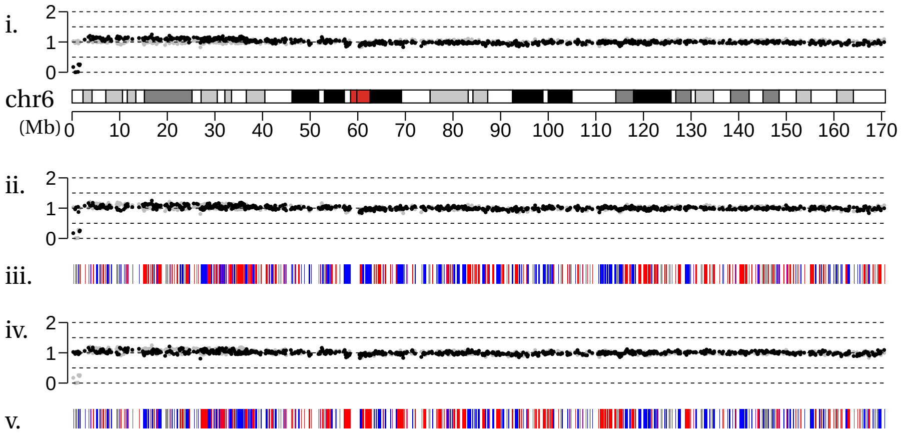

X-33 | Allelic-imbalance based haplotype phasing on Chrs.1q,8,18,X. The subclonal 1q loss cannot be resolved due to its low clonality ≈ 0.09. The switching error on 8p is due to inaccuracies in the allelic depth in a polymorphic region and can be corrected manually. Note the correct phasing of segments in allelic imbalance on 8q that are interspersed with regions in allelic imbalance.

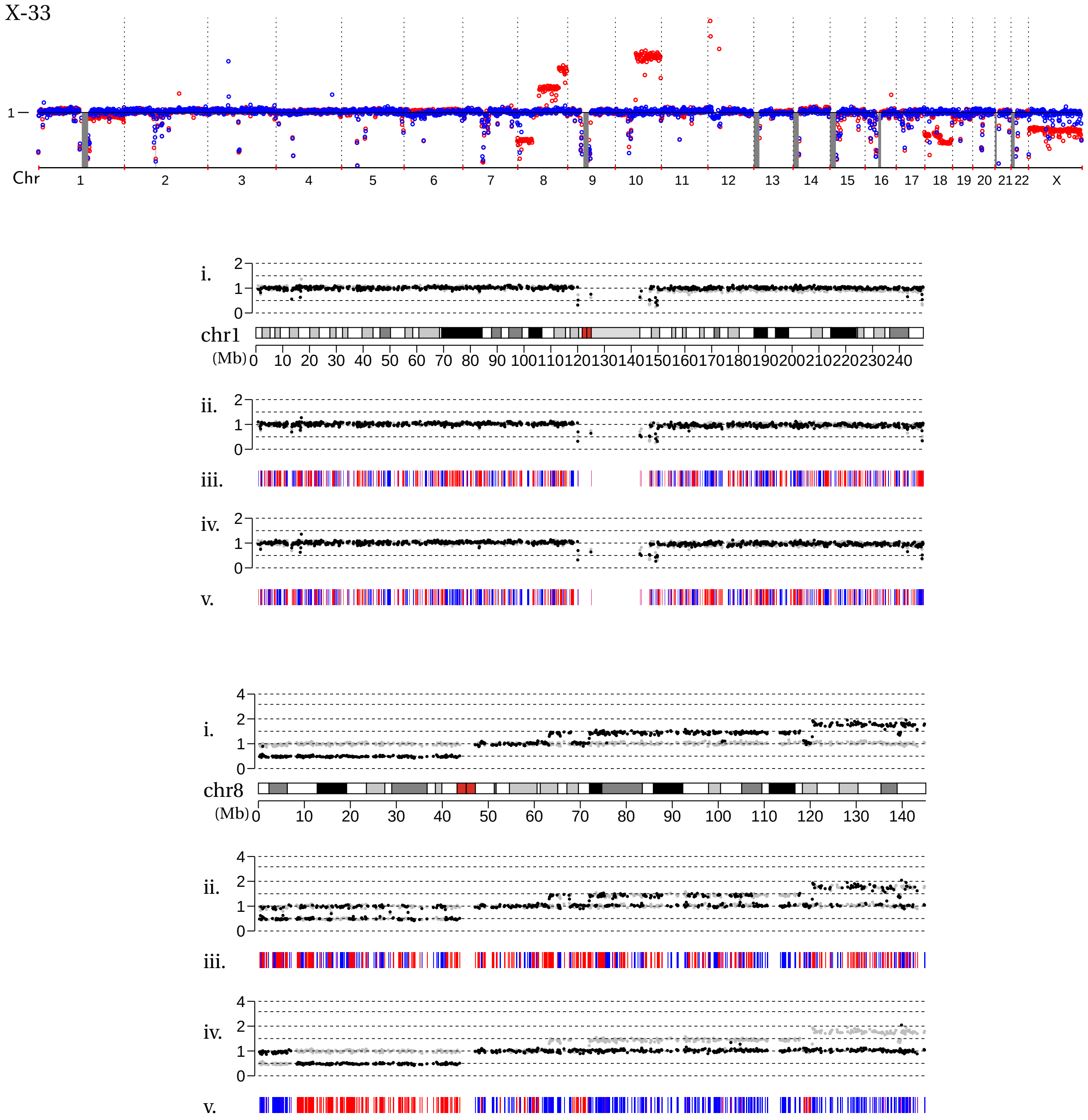

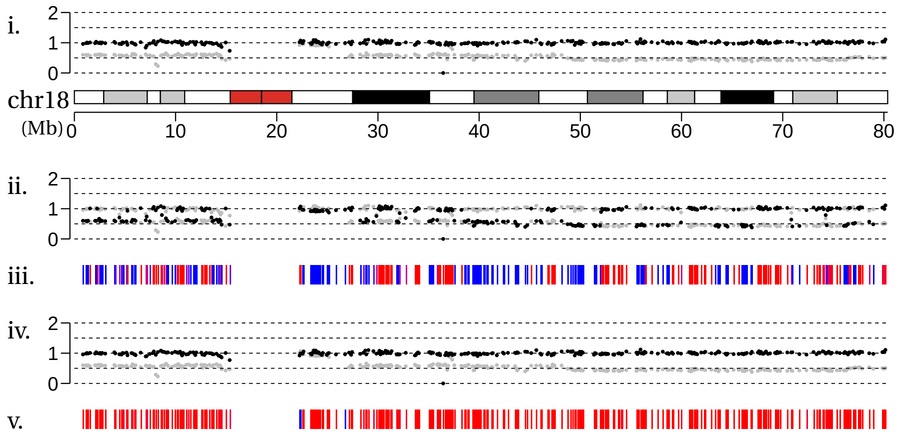

i.
chr18
(Mb)
0
10
20
30
40
50
60
70
80
ii.
iii.
iv.
v.

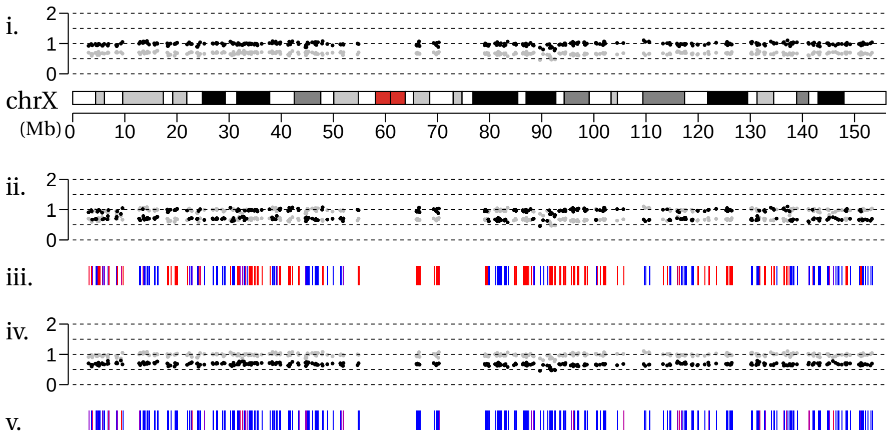

i.
chrX
(Mb)
0
10
20
30
40
50
60
70
80
90
100
110
120
130
140
150
ii.
iii.
iv.
v.

I-dox-1 | Allelic-imbalance based haplotype phasing on Chrs.2,7,8,11,15. Note the variation in allelic depths near sites of long-range switching errors on 2q and 7q.

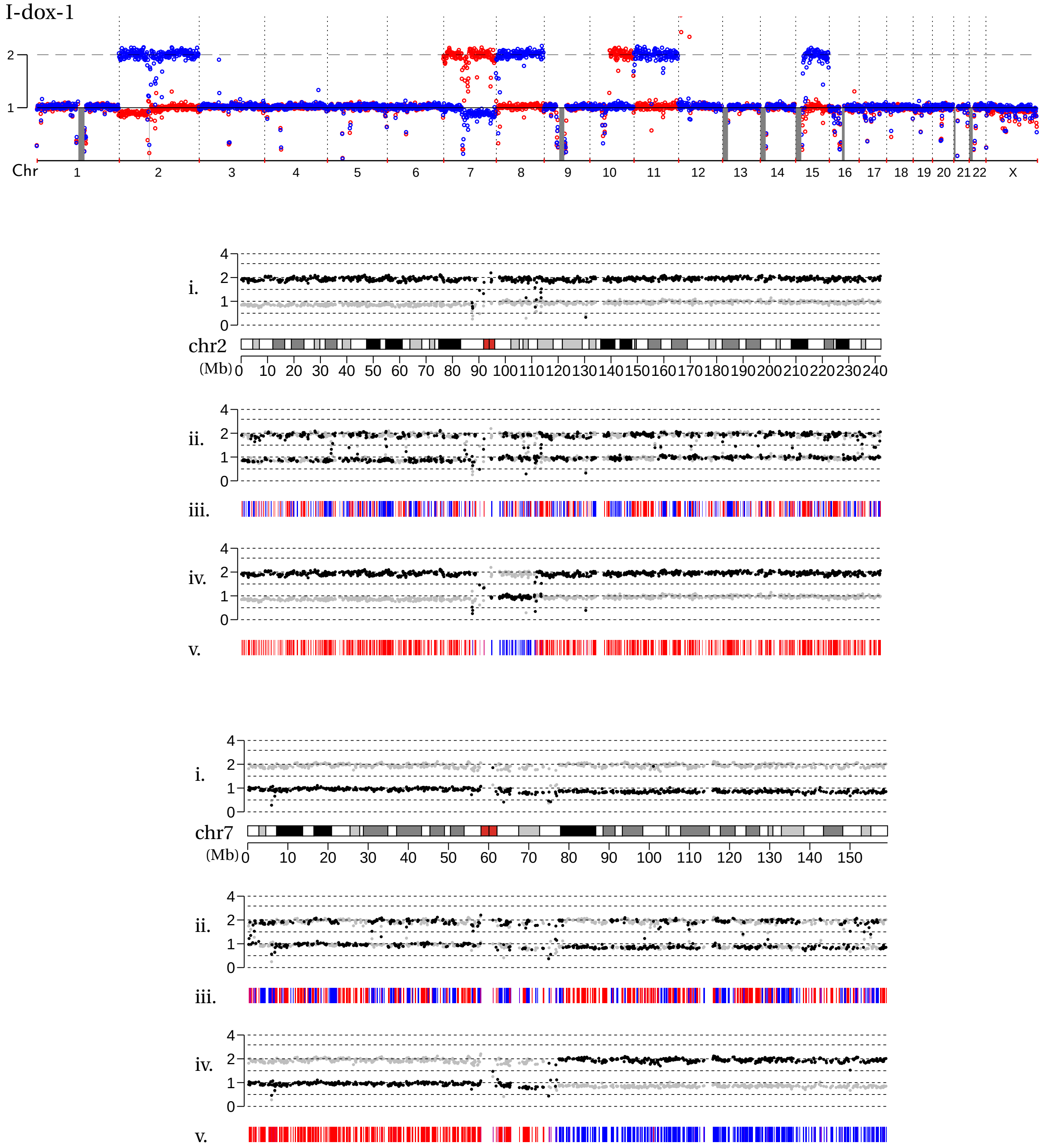

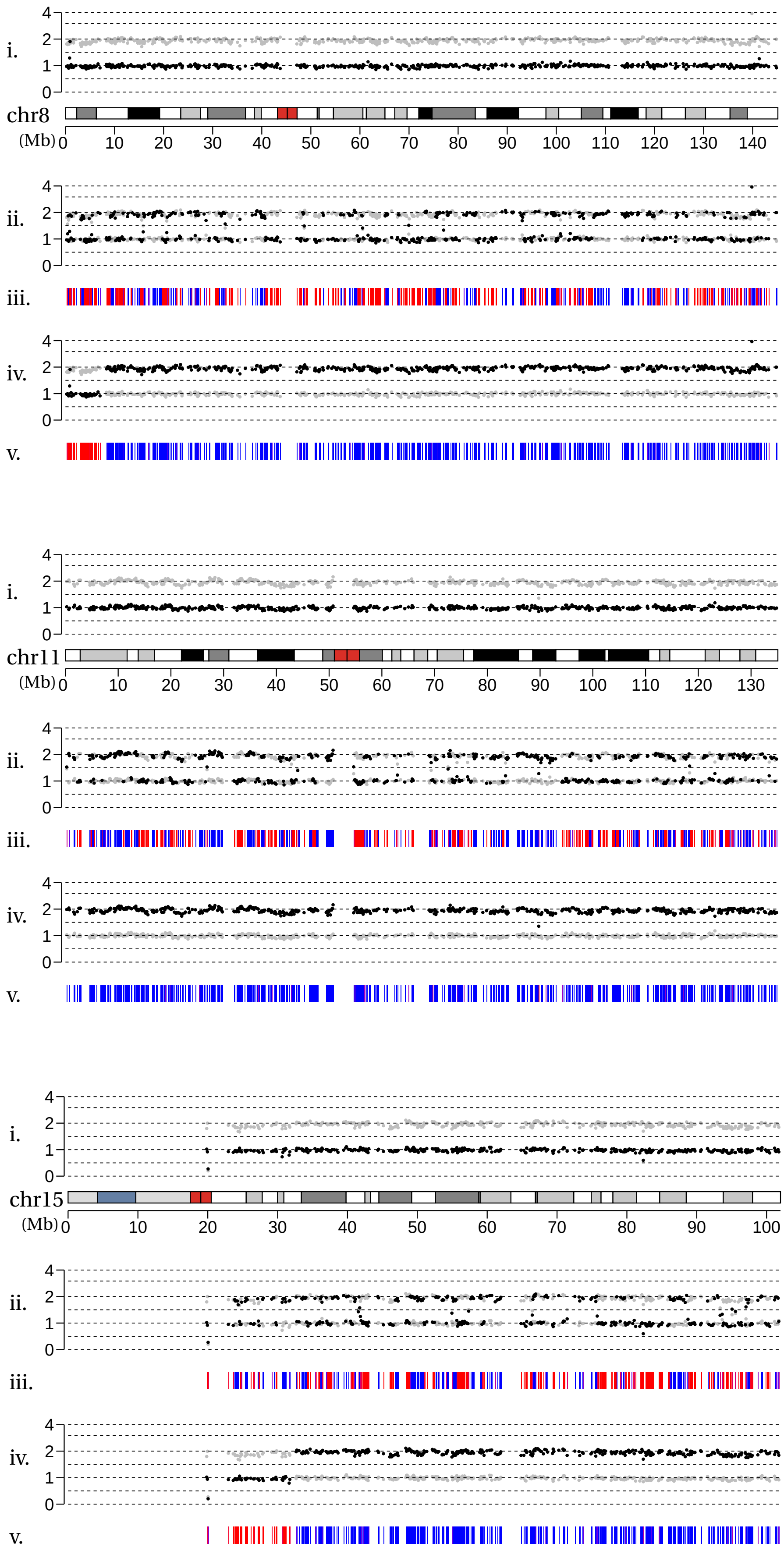
i.
ii.
iii.
iv.
v.
chr8
chr11
chr15
(Mb)
0 10 20 30 40 50 60 70 80 90 100 110 120 130 140
0 10 20 30 40 50 60 70 80 90 100 110 120 130
0 10 20 30 40 50 60 70 80 90 100
0 1 2 4

X-25 | Allelic-imbalance based haplotype phasing on Chrs.4p,8q,12q,13q,X. Note the correct haplotype phase inference in regions of 13q-terminal deletion and the flanking duplication.

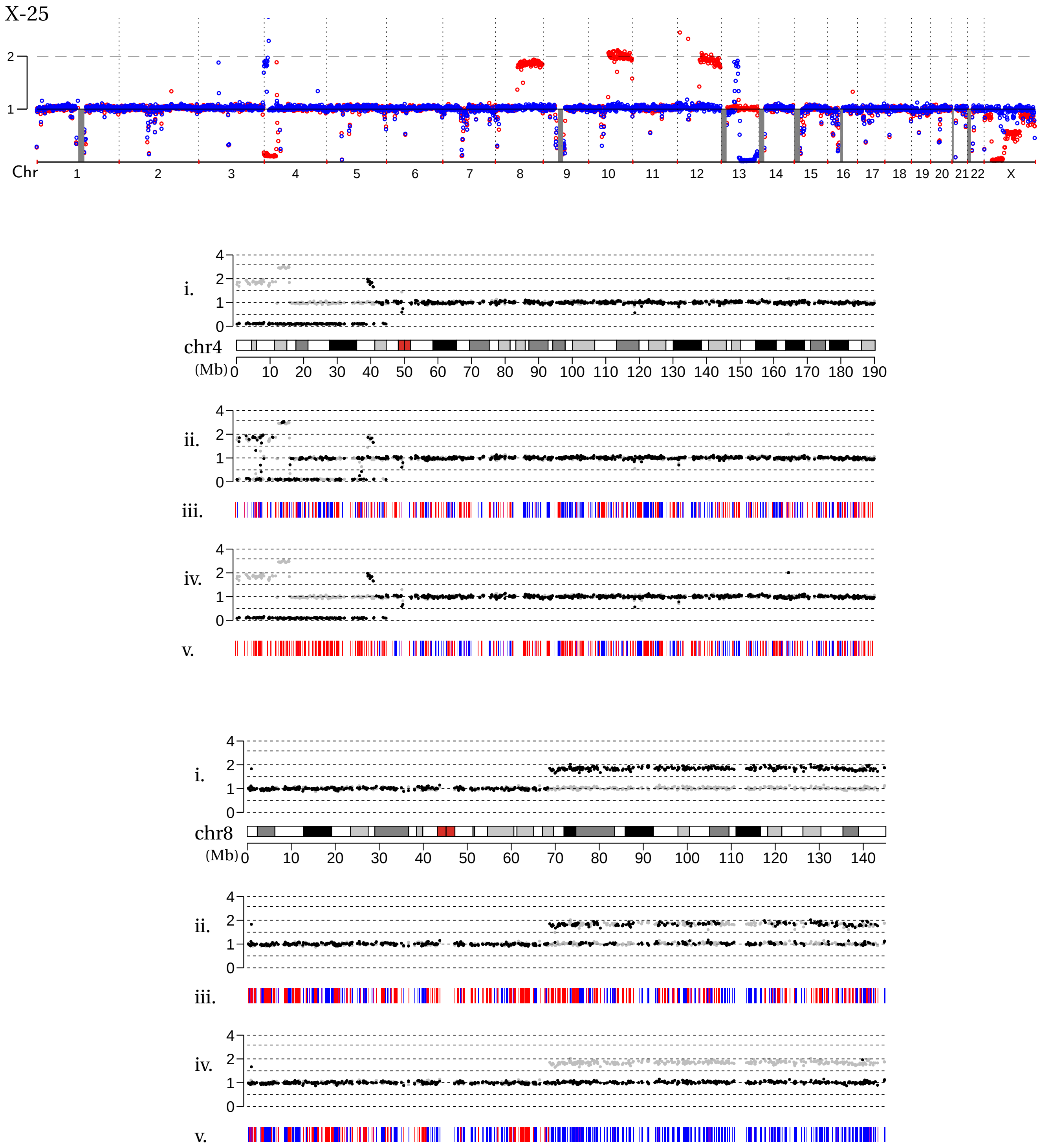

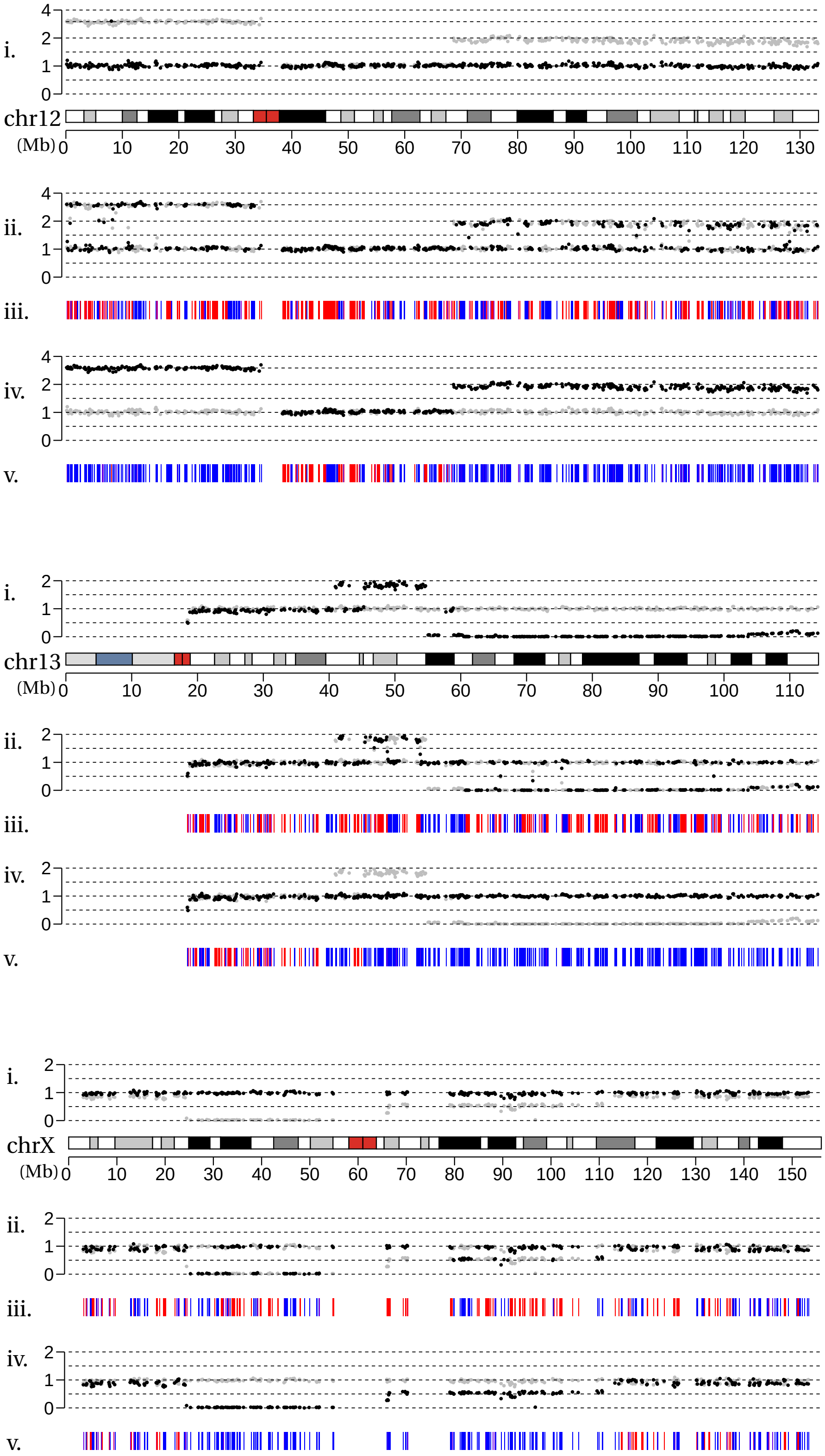
i.
chr12
(Mb)
ii.
iii.
iv.
v.
i.
chr13
(Mb)
ii.
iii.
iv.
v.
i.
chrX
(Mb)
ii.
iii.
iv.
v.

X-37 | Allelic-imbalance based haplotype phasing on Chrs.1,14q,15q,17p,18q,22q. The subclonal 1p and 22q losses cannot be resolved due to low clonality. Note the sloping copy-number patter at the 15q terminus.

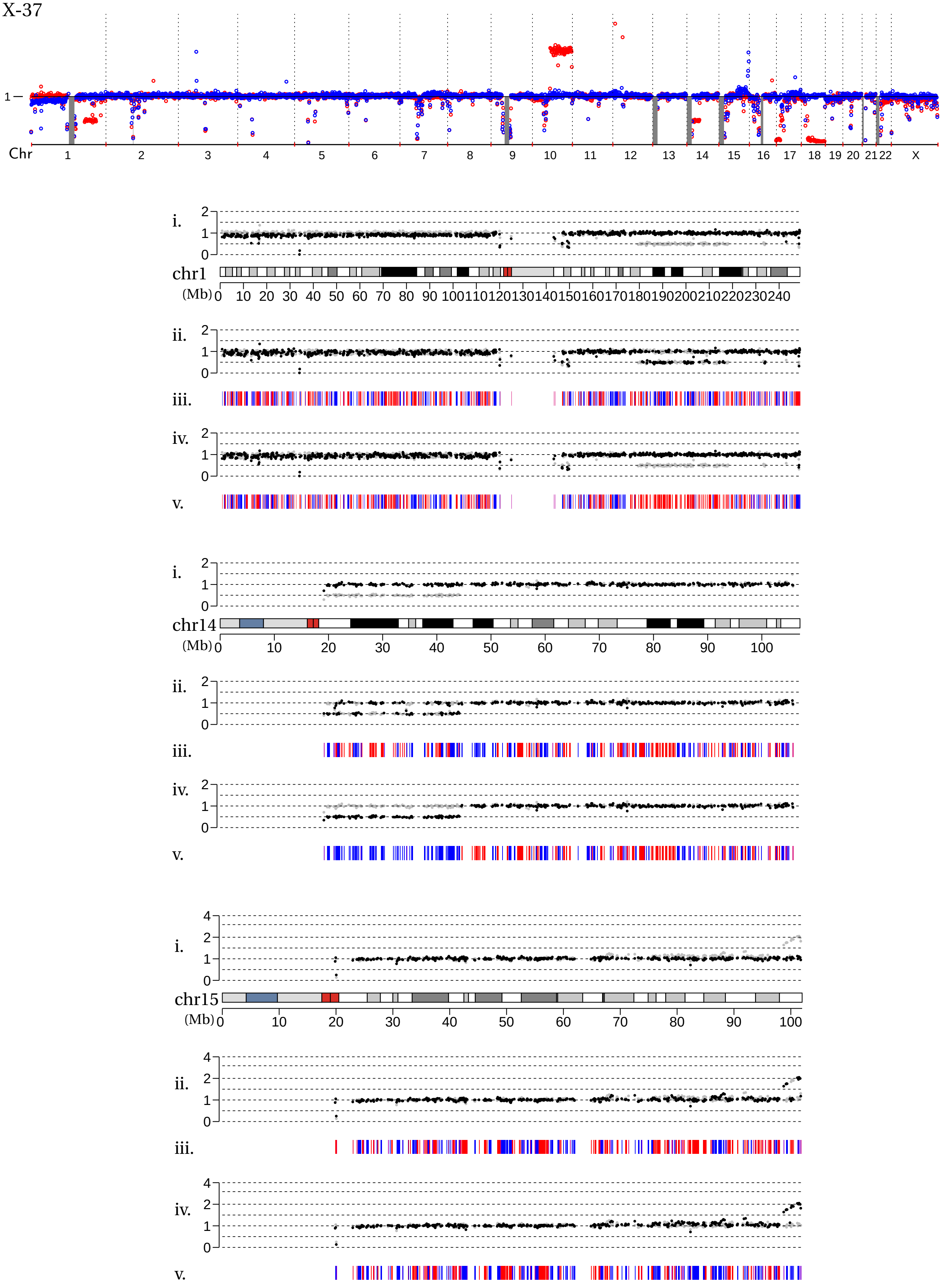

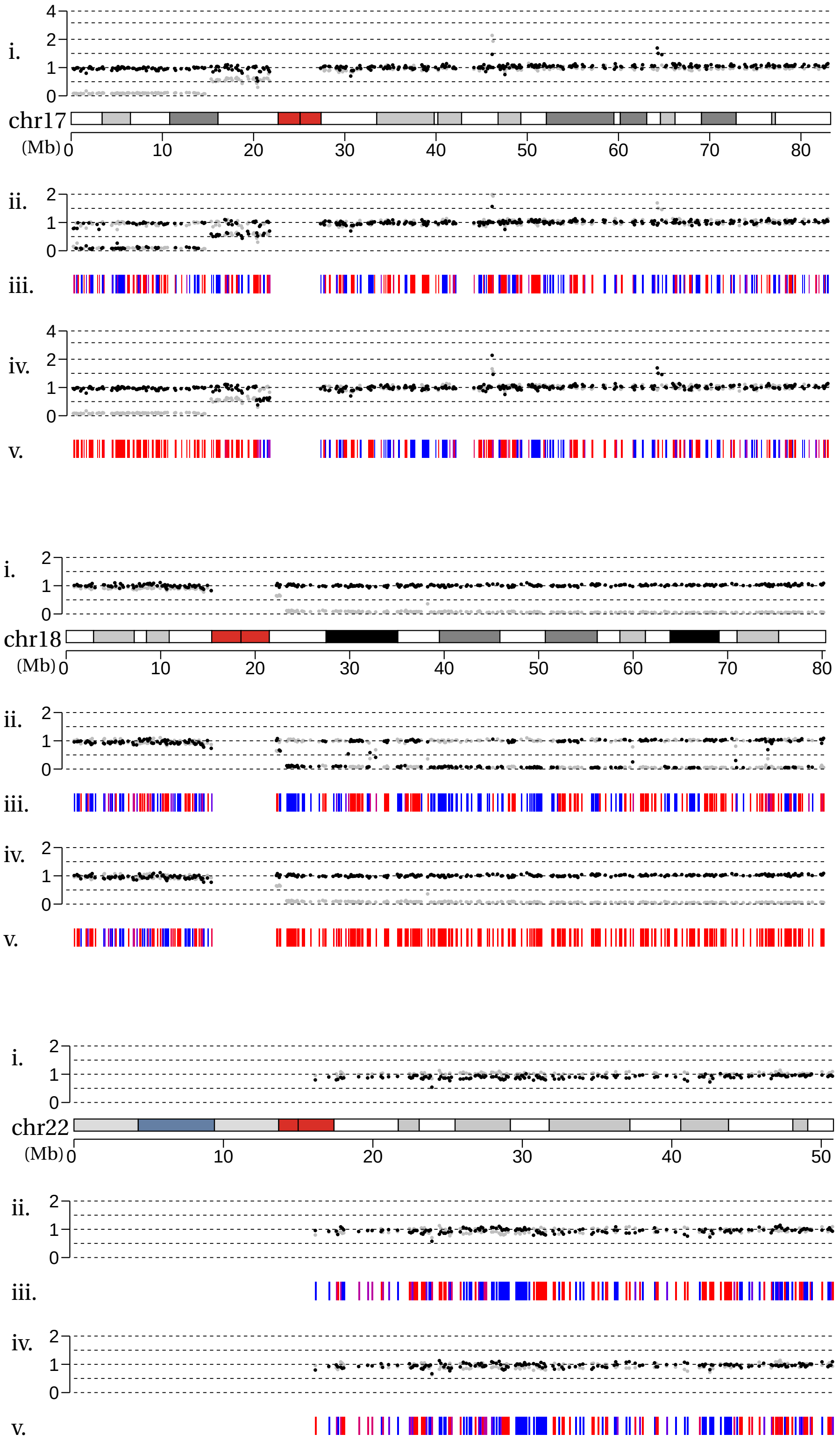
i.
chr17
(Mb)
ii.
iii.
iv.
v.
i.
chr18
(Mb)
ii.
iii.
iv.
v.
i.
chr22
(Mb)
ii.
iii.
iv.
v.

X-36 | Allelic-imbalance based haplotype phasing on Chrs.2,4,6,7,10p,18. Note sloping copy-number patterns on 2p (high copy-number homolog) and near the 6q terminus.

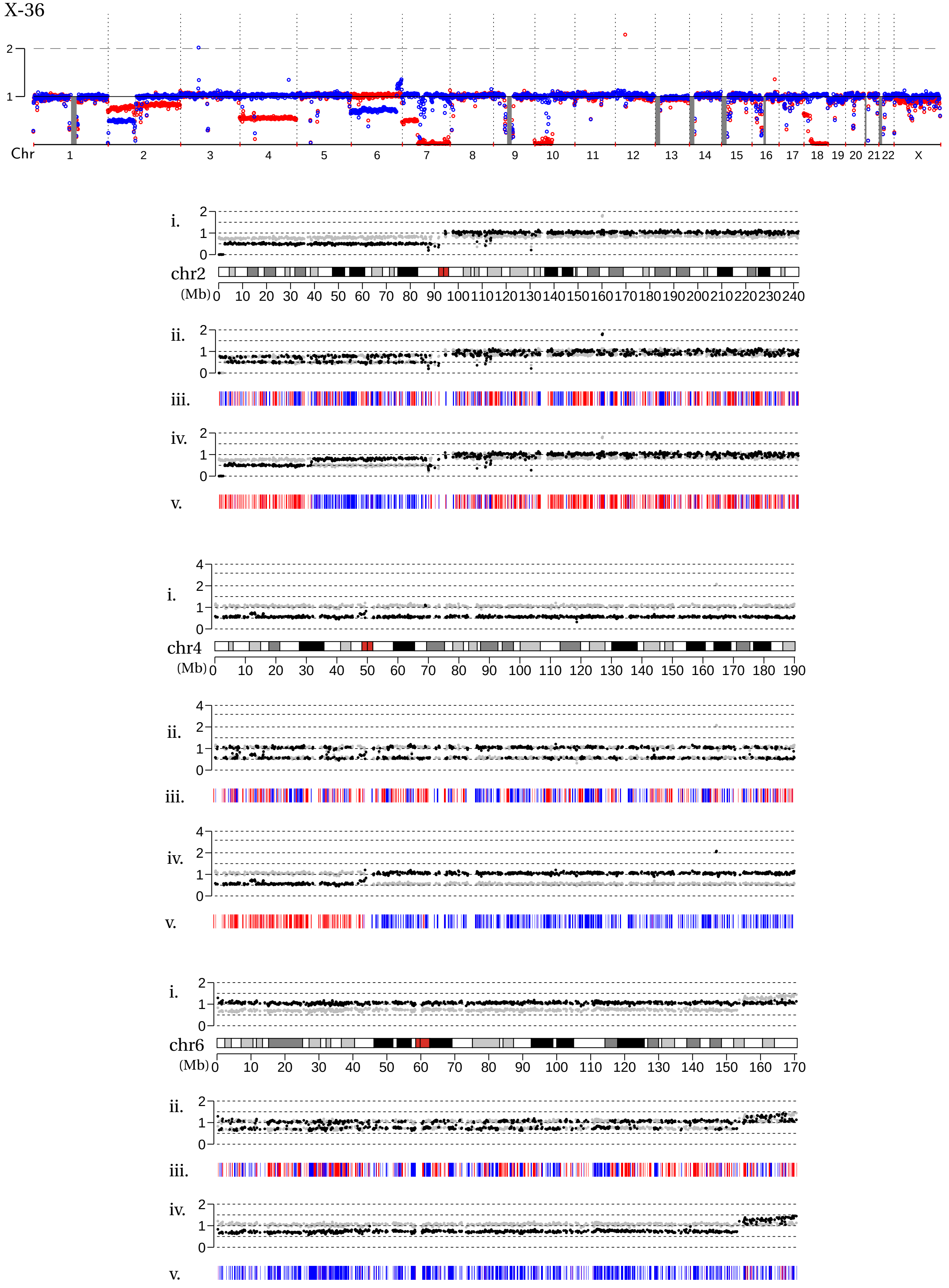

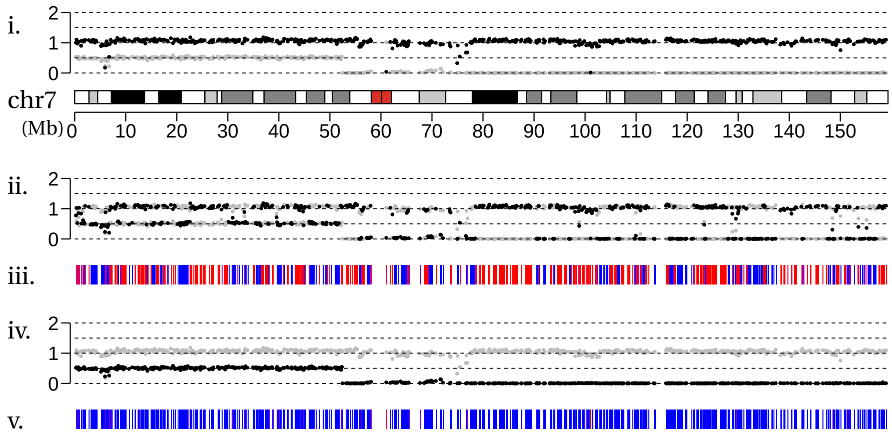
i.
ii.
iii.
iv.
v.
chr7
(Mb) 0 10 20 30 40 50 60 70 80 90 100 110 120 130 140 150

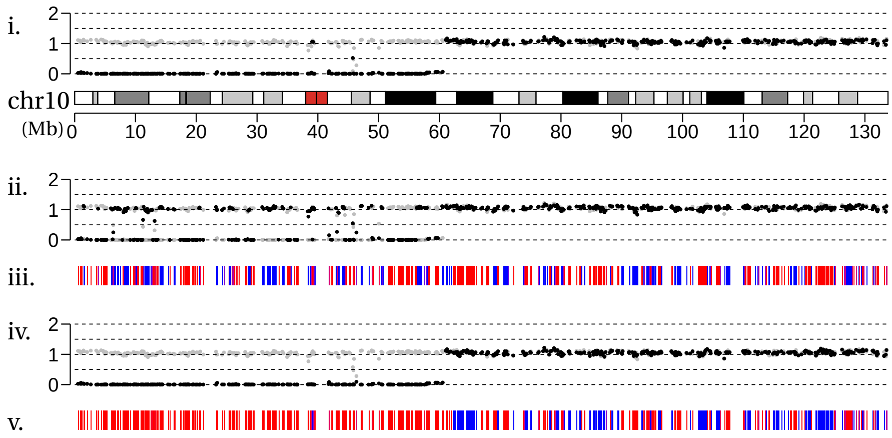
i.
ii.
iii.
iv.
v.
chr10
(Mb) 0 10 20 30 40 50 60 70 80 90 100 110 120 130

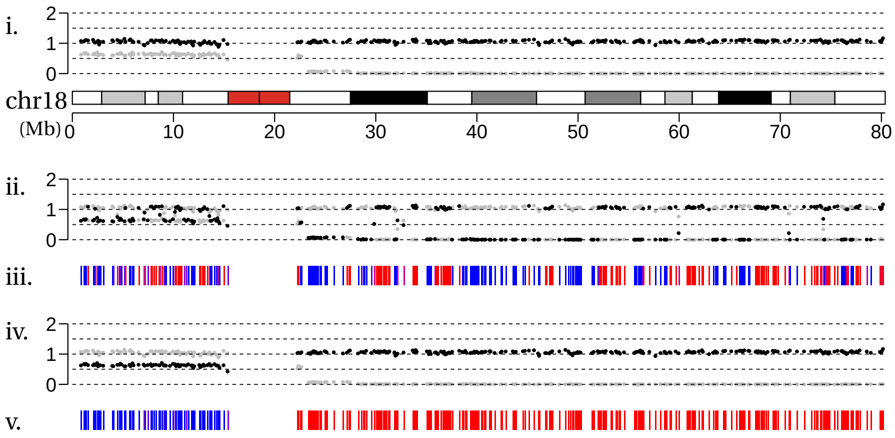
i.
ii.
iii.
iv.
v.
chr18
(Mb) 0 10 20 30 40 50 60 70 80

X-35 | Allelic-imbalance based haplotype phasing on Chrs.1q,5q,6p,8p,10,12p,16,18. Note sloping copy-number patterns on 5q, near the 6p terminus, and on Chr.18 (both arms).

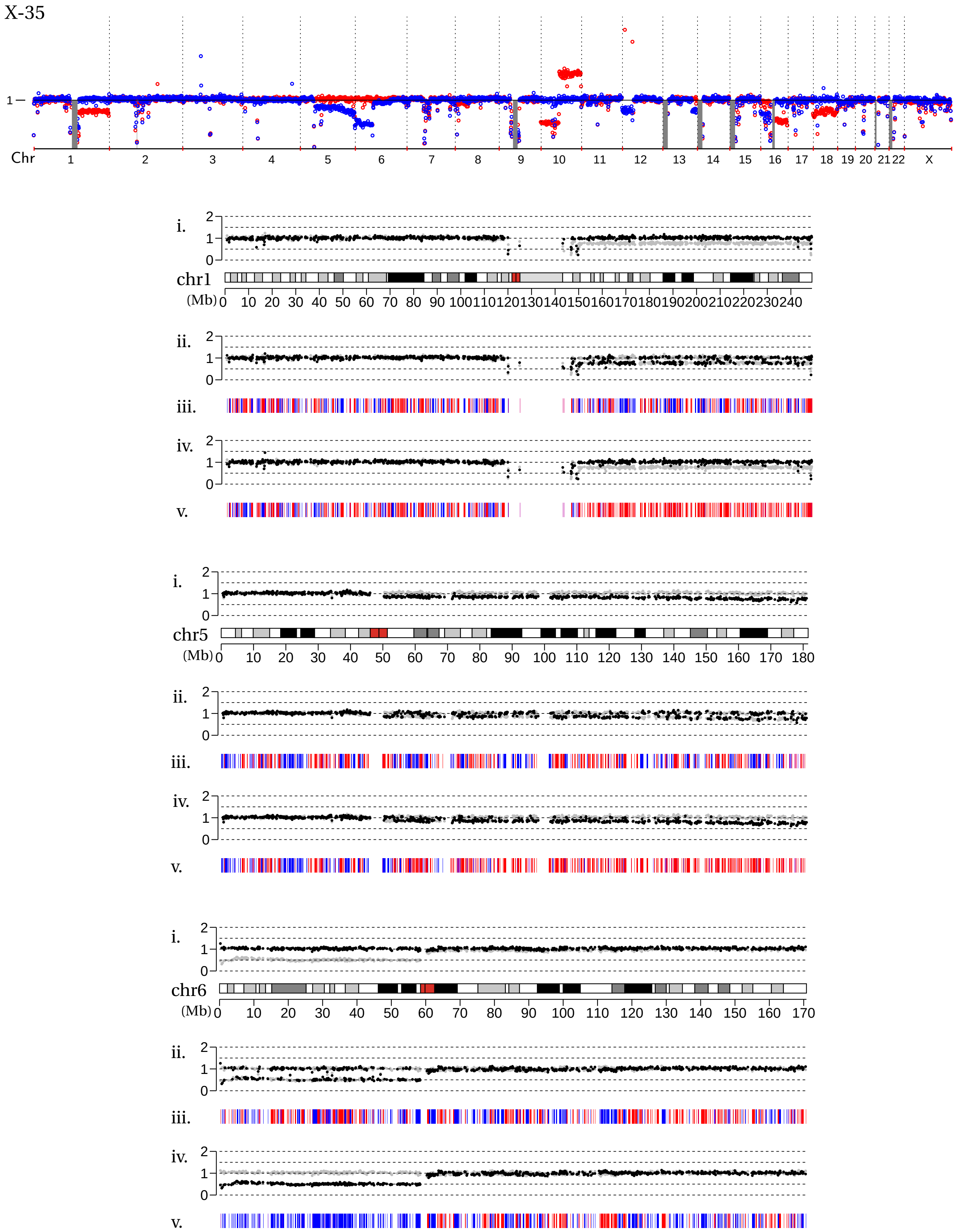

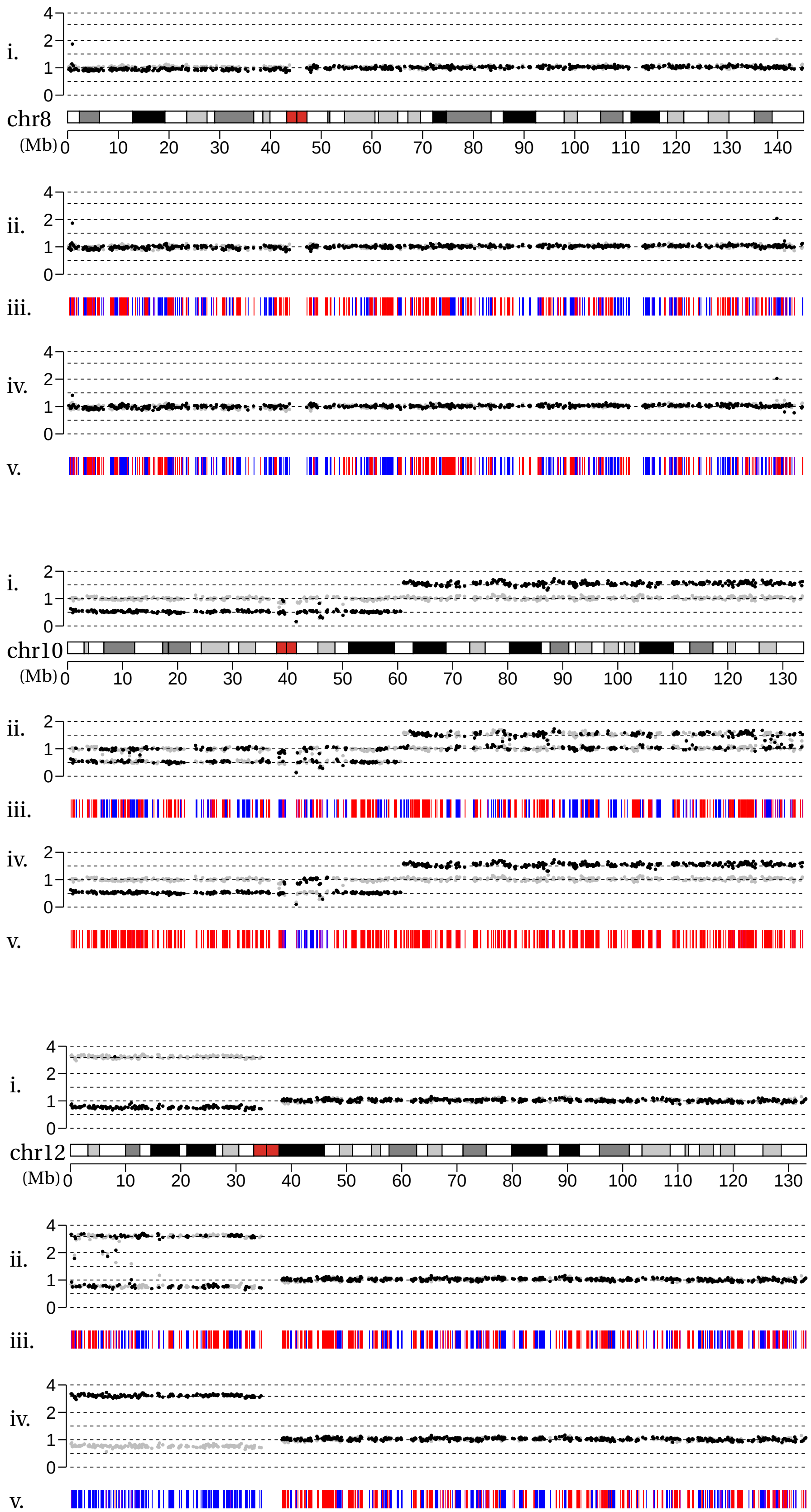
i.
ii.
iii.
iv.
v.
chr8
chr10
chr12
(Mb)
0
10
20
30
40
50
60
70
80
90
100
110
120
130
140

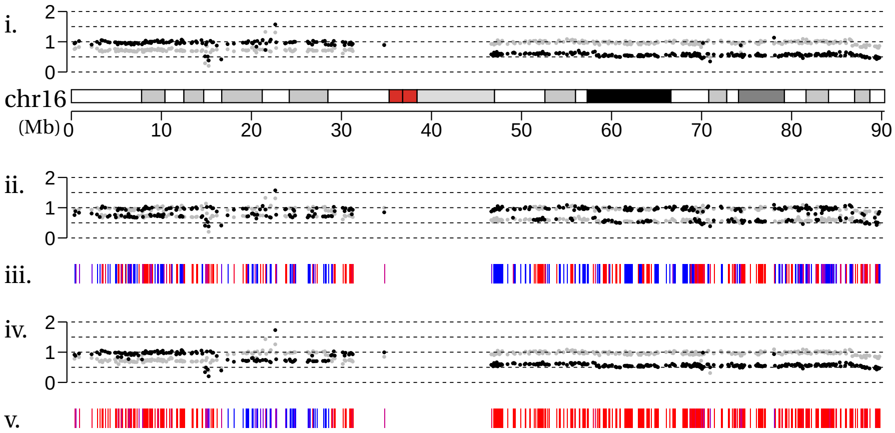
i.
ii.
iii.
iv.
v.
chr16
(Mb)
0 10 20 30 40 50 60 70 80 90
2
1
0

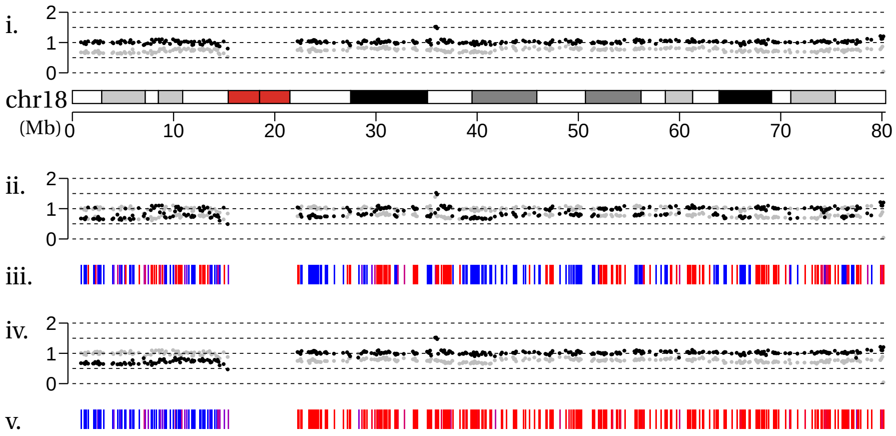
i.
ii.
iii.
iv.
v.
chr18
(Mb)
0 10 20 30 40 50 60 70 80
2
1
0
