# Supplementary material for: Genomic signatures of past and present chromosomal instability in Barrett’s esophagus and early esophageal adenocarcinoma
Source: Nat Commun. 2023 Oct 4;14:6203. doi: 10.1038/s41467-023-41805-6 (PMC10550953; doi:10.1038/s41467-023-41805-6)

25kb phased allelic depth calculated from *in silico* down-sampled allelic coverage

Patient 7, LGD2 (est. tumor cell fraction: 75%; ploidy: 1.95): 28×, 20 × 10×, 5× 1
Patient 7, HGD2 (est. tumor cell fraction: 60%; ploidy: 1.91): 30×, 20×, 10×, 5× 2
Patient 7, LGD1 (est. tumor cell fraction: 51%; ploidy: 1.94): 26×, 20×, 10×, 5× 3
Patient 7, EAC (est. tumor cell fraction: 40%; ploidy: 3.85): 23×, 20×, 10×, 5× 4
Mixtures of HGD2 and the normal reference from Patient 7 at 20× with est. tumor cell fractions of 40-10% 5
Mixtures of EAC and the normal reference from Patient 7 at 20× with est. tumor cell fractions of 40-10% . 7

Patient 7, LGD2. Purity: 75%; ploidy: 1.95 | Allelic depths in 25kb intervals calculated from down-sampled allelic coverage on Chr.18 with mean sequencing depths of 28×, 20×, 10×, 5× based on Eq. (1) in **Online Methods**. Left panels show the allelic depths along Chr.18: Black and gray dots represent the normalized depth of each allele ("allele A" and "allele B"). Right panels show the scatter plots and distributions of allelic copy-number states: X- and Y-axis represent allele A and allele B depths; dotted gray lines represent allelic depths (annotated with numbers in gray) corresponding to integer copy-number states 0, 1, 2, ...; the contour map represent the 2D distribution of allelic copy-number states, the histograms on each axis represent the 1D distribution of allelic copy-number states. The reduced allelic depth on 18q is inferred to be a clonal deletion (clonal fraction 75%). The separation between different allelic copy-number states (in both 2D and 1D distributions) ensures the accuracy of long-range haplotype phasing based on allelic depth differences.

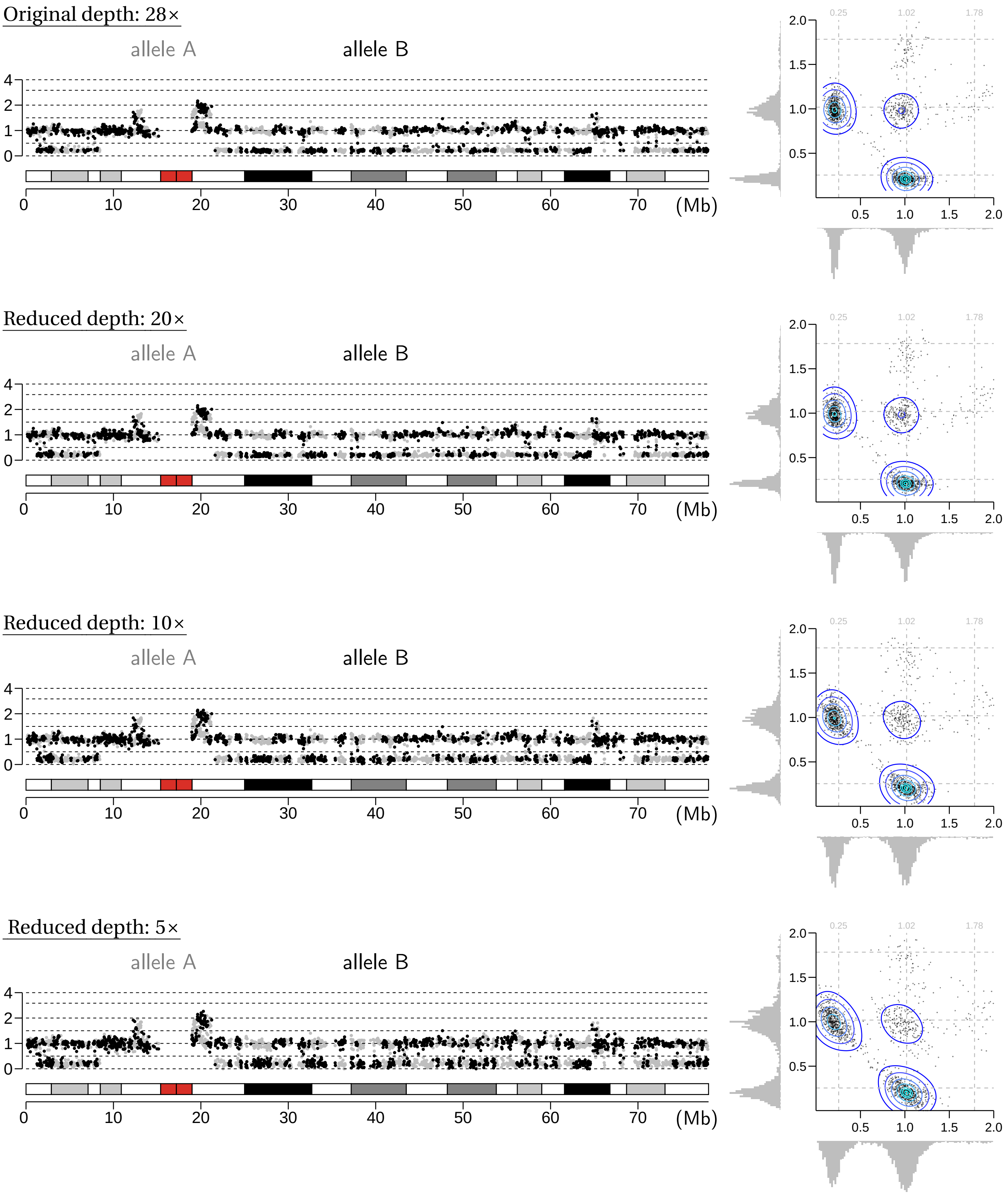

Patient 7, HGD2. Purity: 60%; ploidy: 1.91 | Allelic depths in 25kb intervals calculated from down-sampled allelic coverage on Chr.18 with mean sequencing depths of 30×, 20×, 10×, 5×.

Original depth: 30×

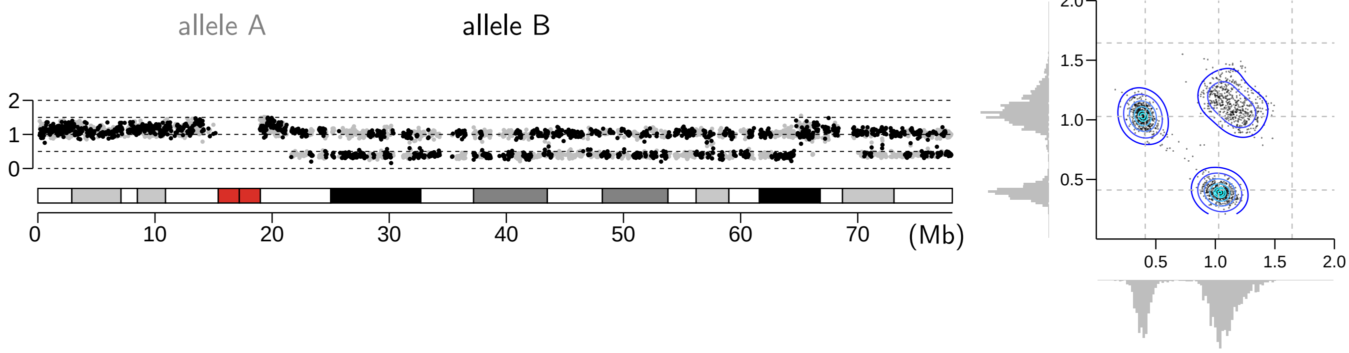

Reduced depth: 20×

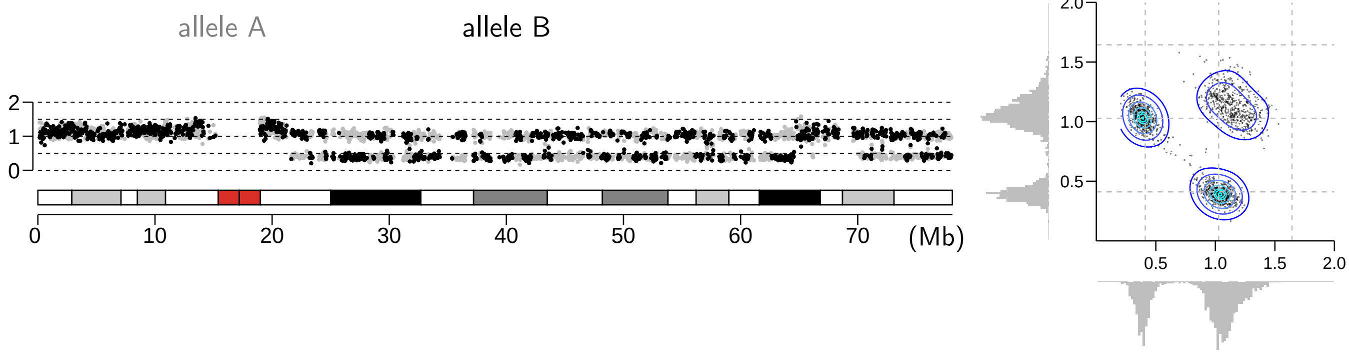

Reduced depth: 10×

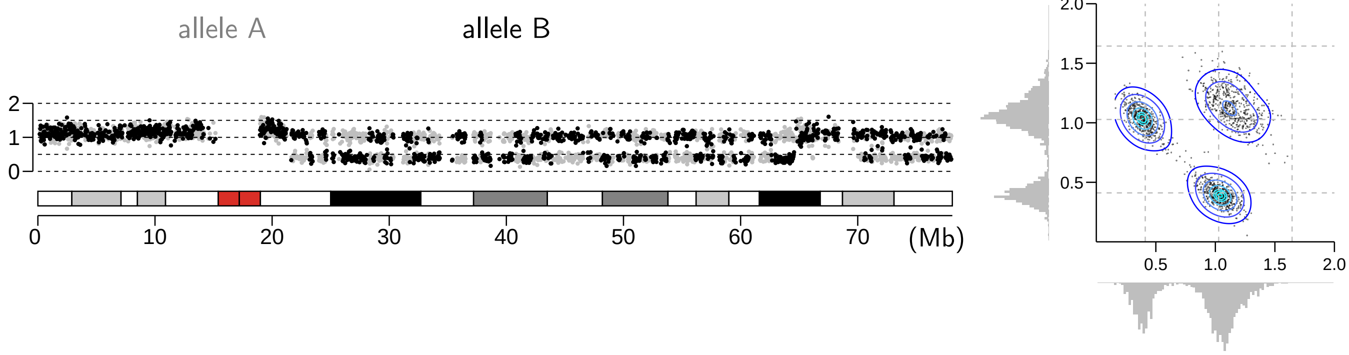

Reduced depth: 5×

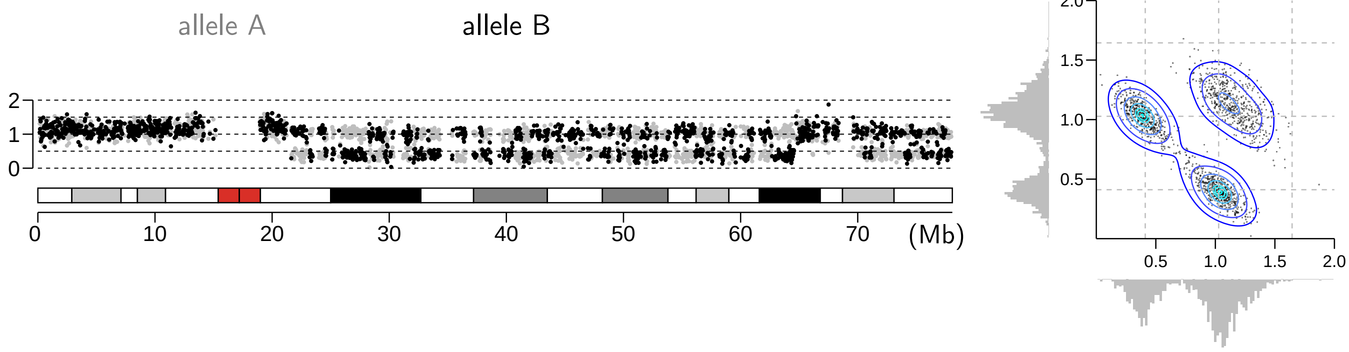

Patient 7, LGD1. Purity: 51%; ploidy: 1.94 | Allelic depths in 25kb intervals calculated from down-sampled allelic coverage on Chr.18 with mean sequencing depths of 26×, 20×, 10×, 5×

Original depth: 26×

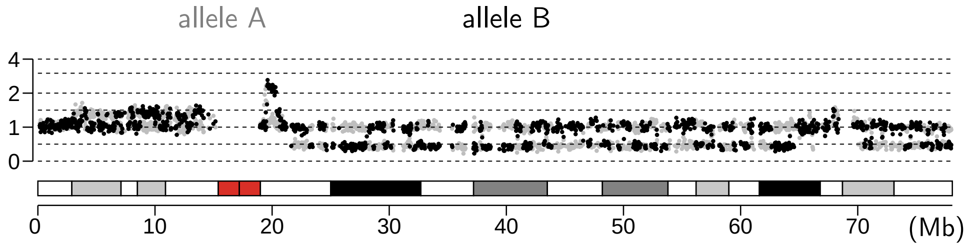

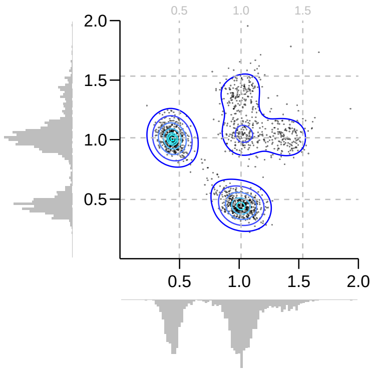

Reduced depth: 20×

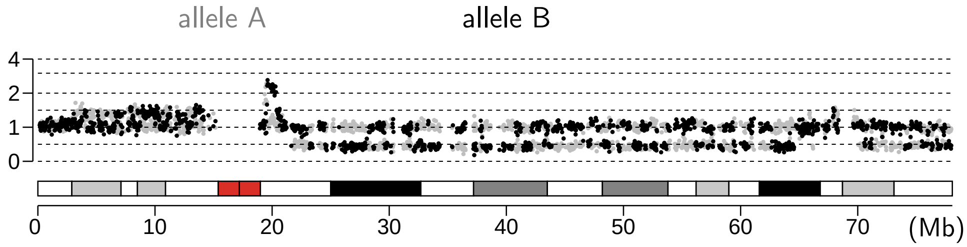

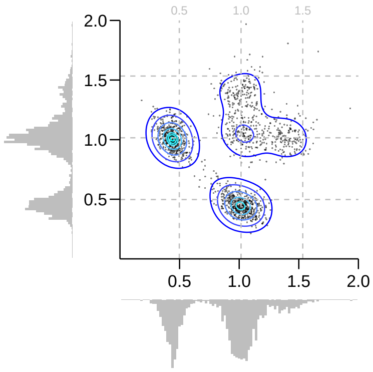

Reduced depth: 10×

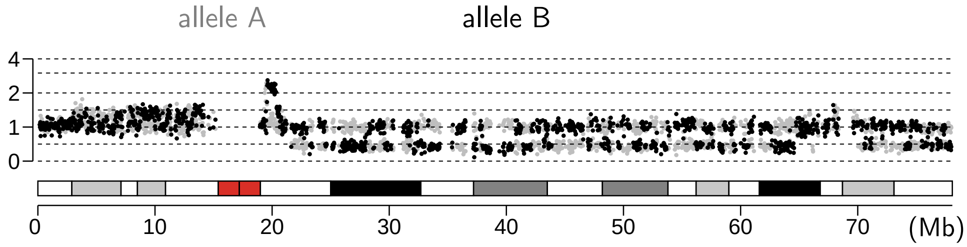

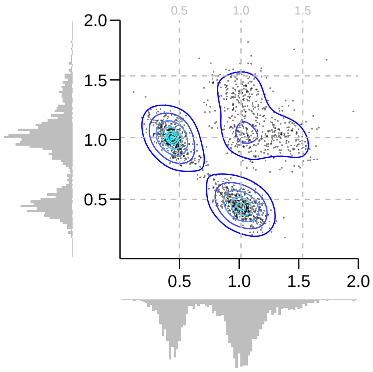

Reduced depth: 5×

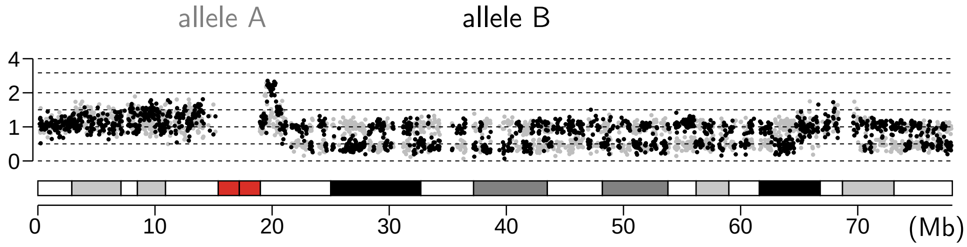

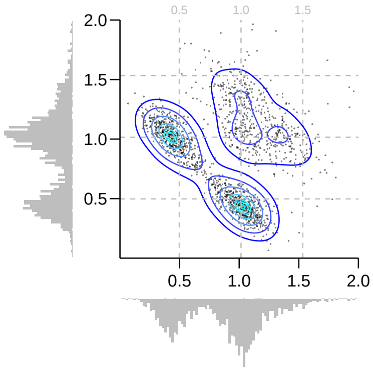

Patient 7, EAC. Purity: 40%; ploidy: 3.85 | Allelic depths in 25kb intervals calculated from down-sampled allelic coverage on Chr.18 with mean sequencing depths of 23×, 20×, 10×, 5×

Original depth: 23×

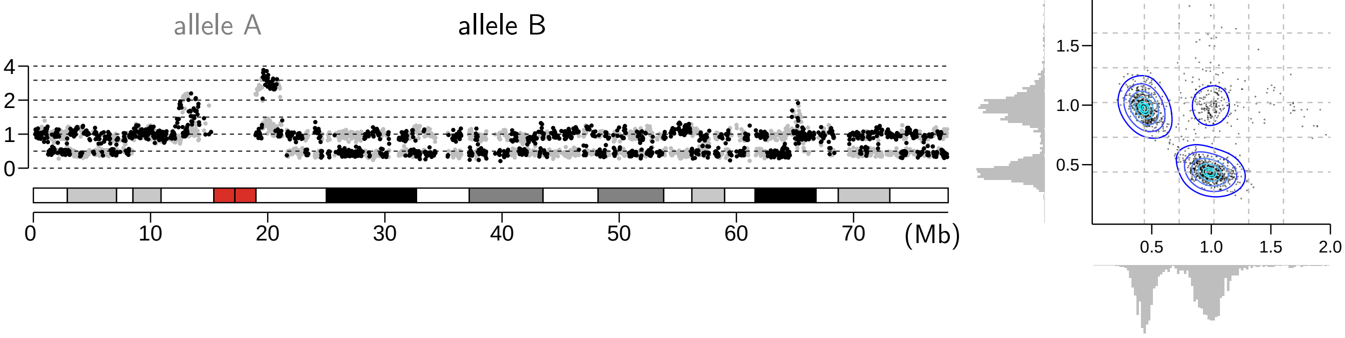

Reduced depth: 20×

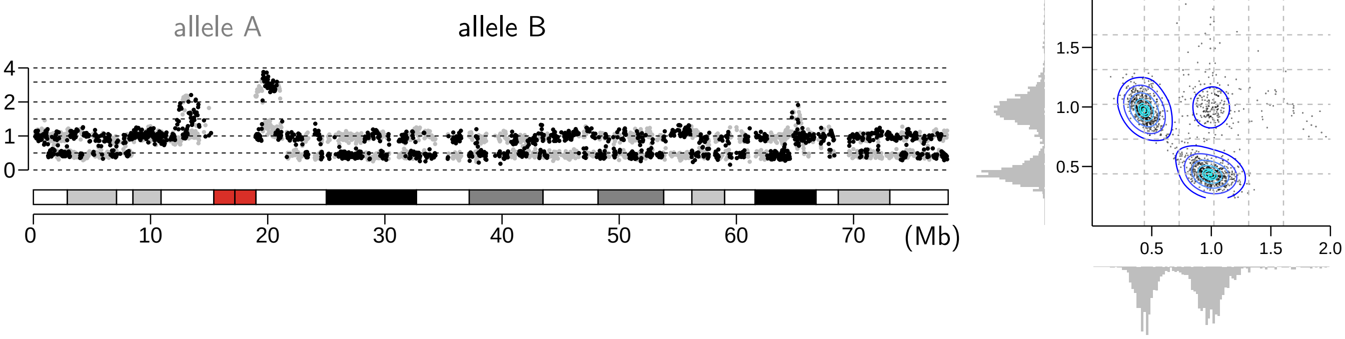

Reduced depth: 10×

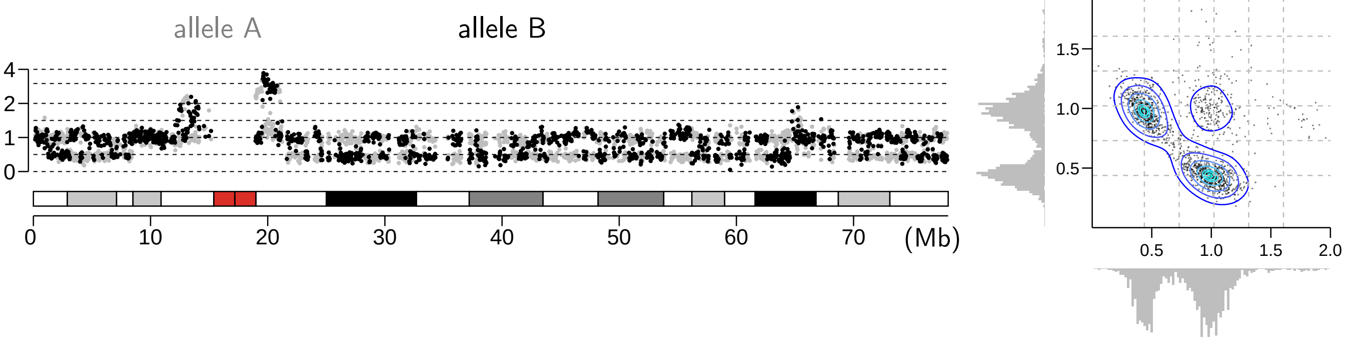

Reduced depth: 5×

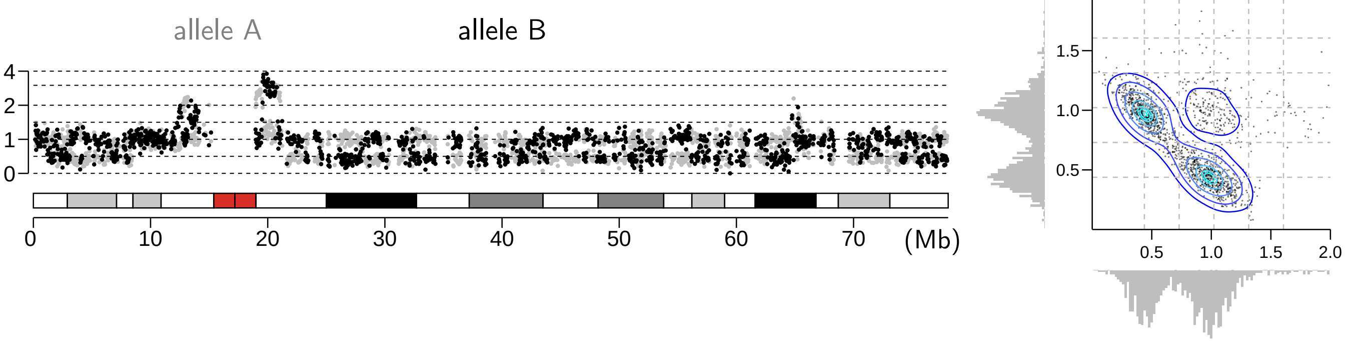

Mixture of Patient 7, HGD2 with Normal @ 20× depth | Allelic depths in 25kb intervals calculated from *in silico* mixtures of sequencing reads from the HGD2 sample and from the matching germline reference with 20× total depth and estimated tumor cell fractions of 40%, 30%, 20%, 10%, 18%,16%,14%,12%. Left and right panels are the same as before. Based on the allelic depth distributions, the lowest clonality of single-copy changes that can be resolved from allelic depth differences is 18-20%.

Estimated tumor purity: 40%

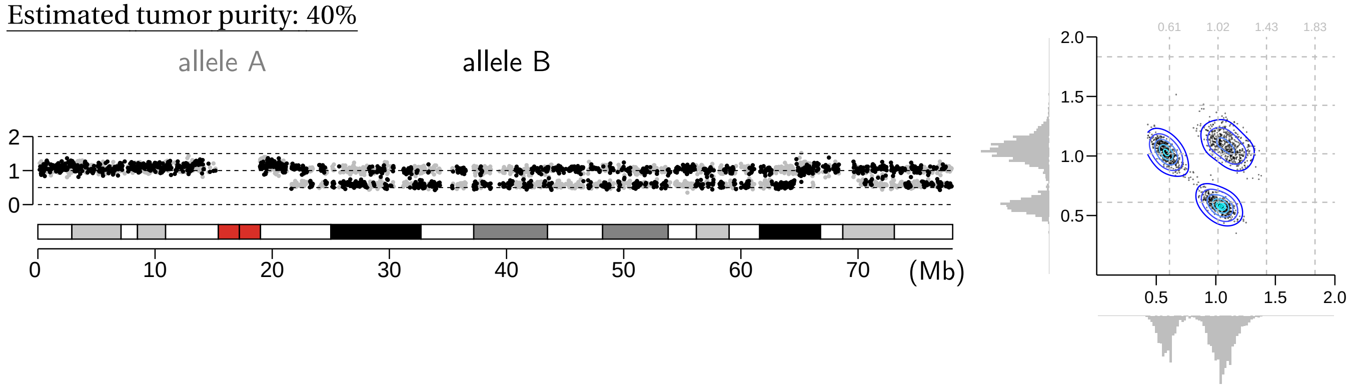

Estimated tumor purity: 30%

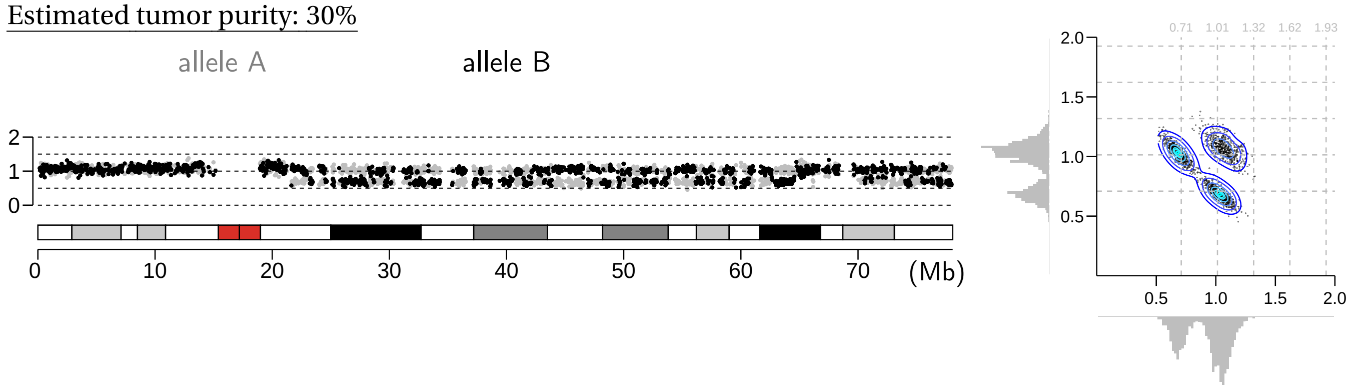

Estimated tumor purity: 20%

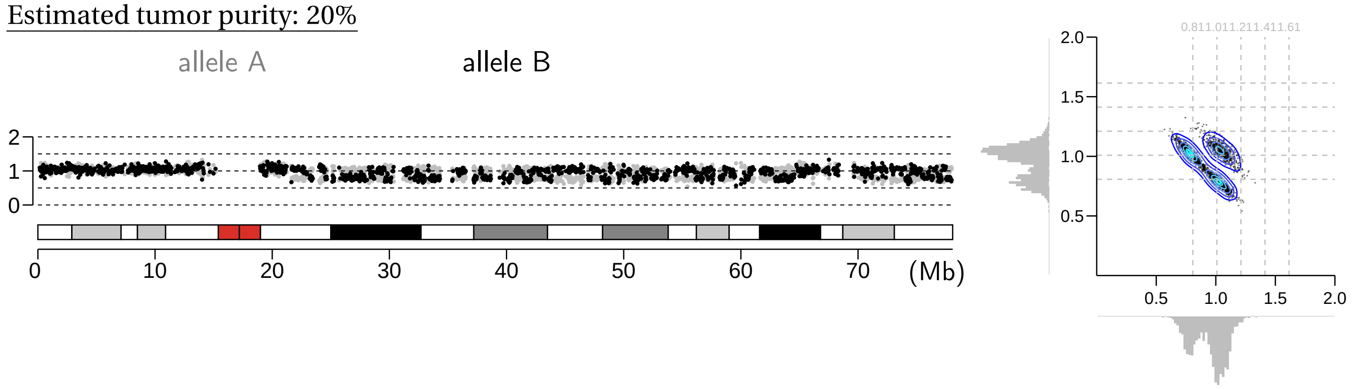

Estimated tumor purity: 10%

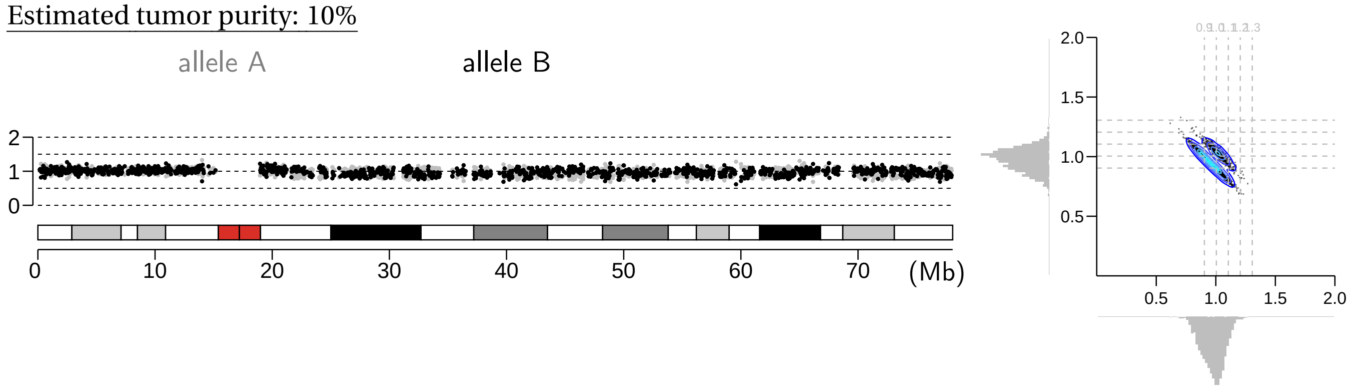

Estimated tumor purity: 18%

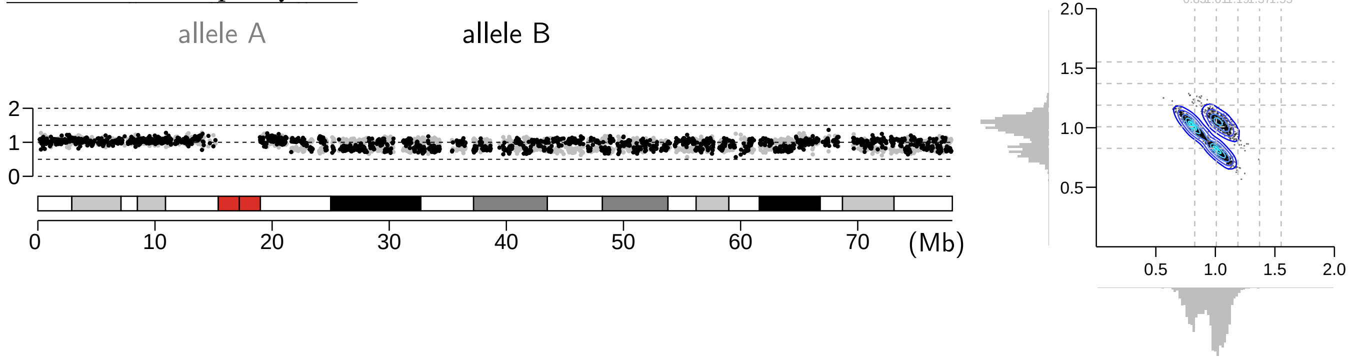

Estimated tumor purity: 16%

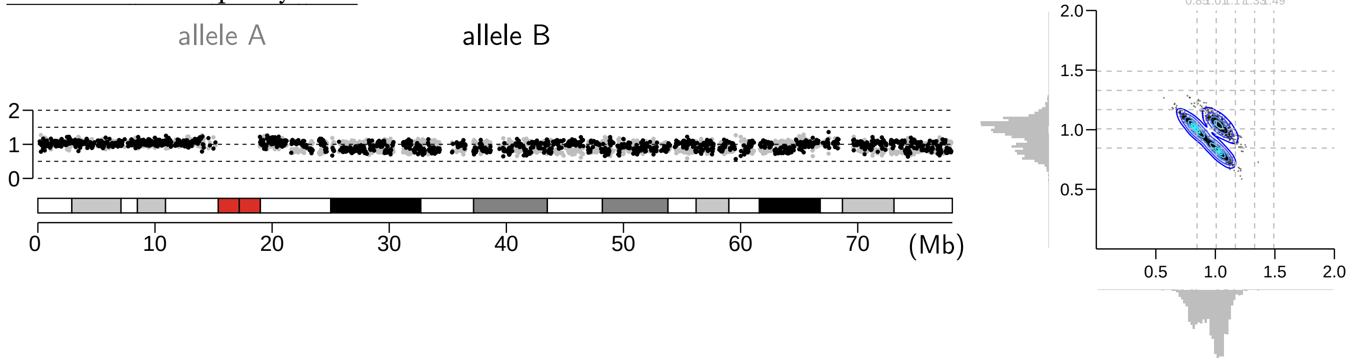

Estimated tumor purity: 14%

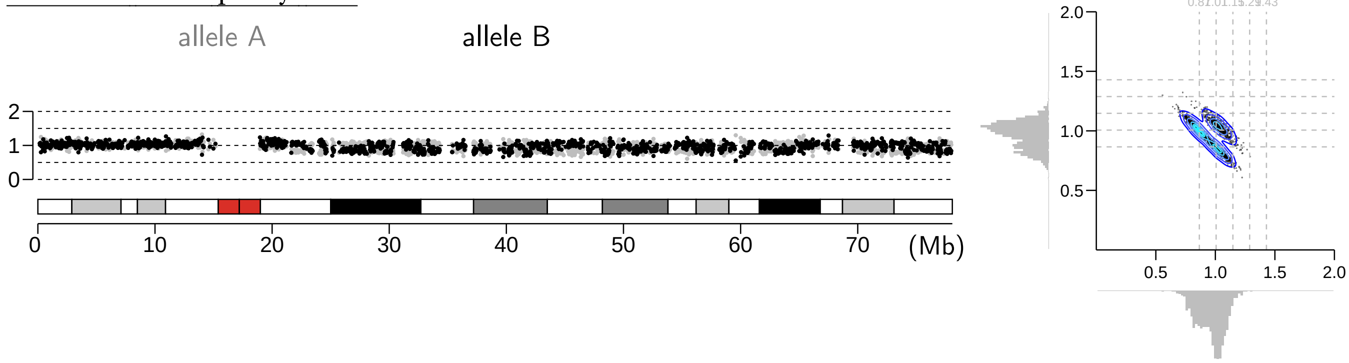

Estimated tumor purity: 12%

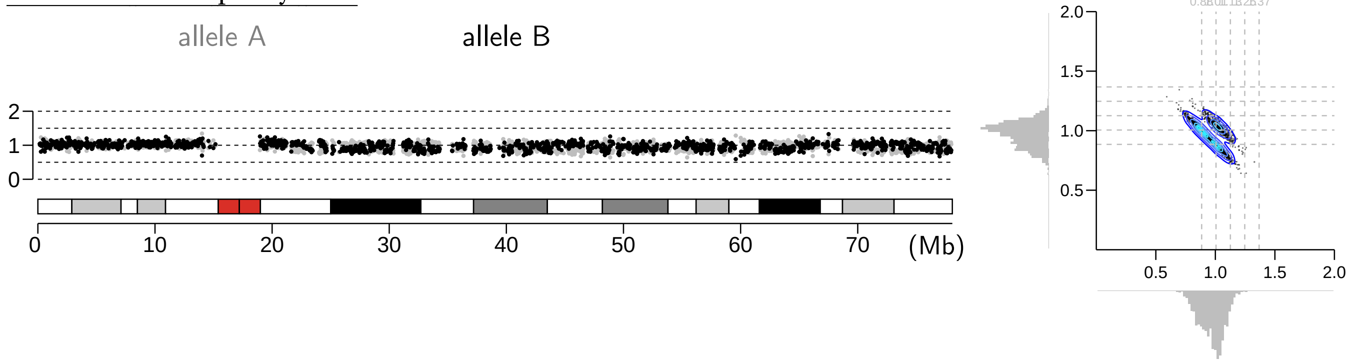

Mixture of Patient 7, EAC with Normal @ 20× depth | Allelic depths in 25kb intervals calculated from *in silico* mixtures of sequencing reads from the EAC sample and from the matching germline reference with 20× total depth and estimated tumor cell fractions of 40%, 30%, 20%, 10%, 18%,16%,14%,12%. Note that the deletion of 18q is inferred to have occurred prior to whole-genome duplication. Therefore, the difference in allelic depths reflects a two-copy difference in the allelic copy number.

Estimated tumor purity: 40%

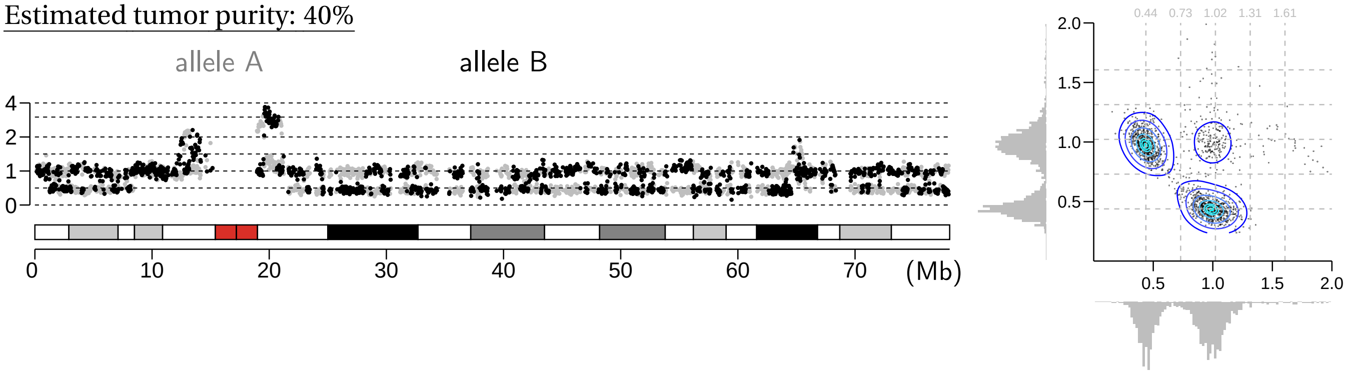

Estimated tumor purity: 30%

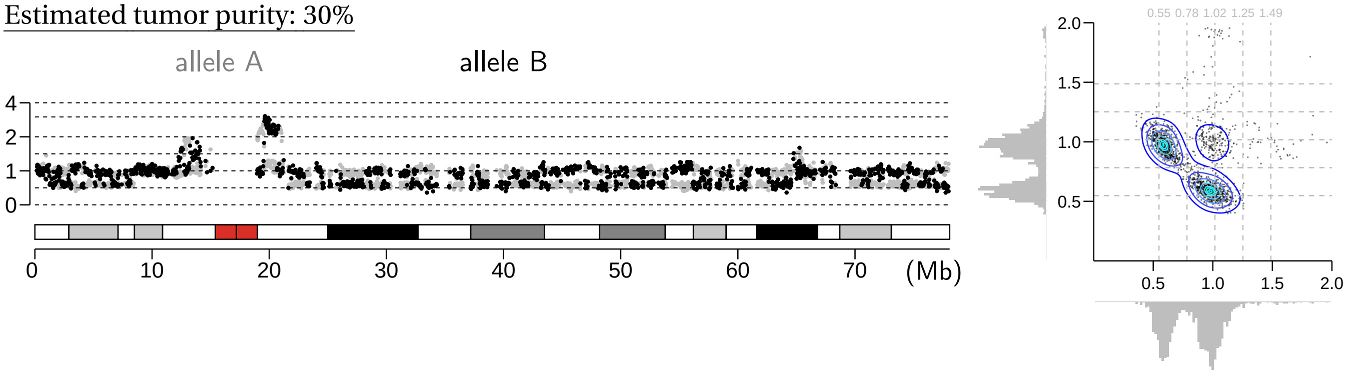

Estimated tumor purity: 20%

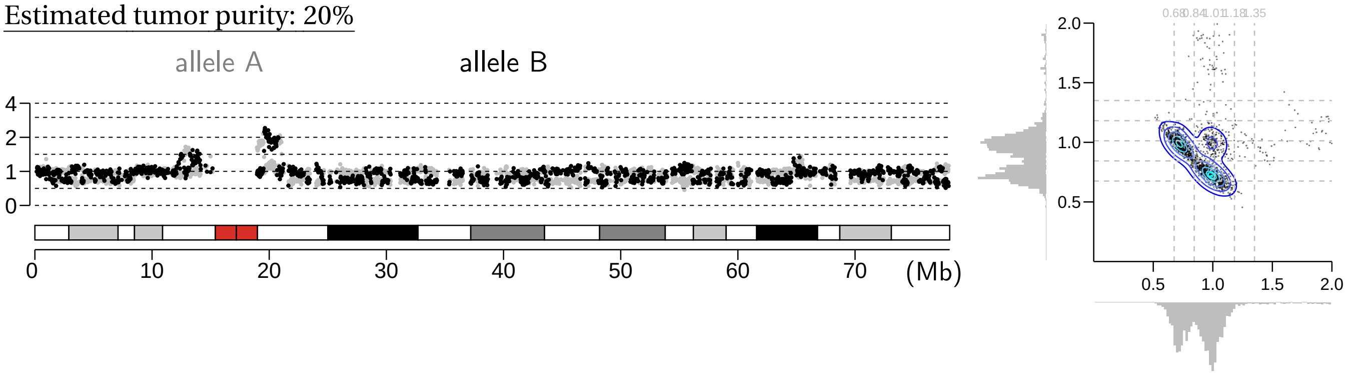

Estimated tumor purity: 10%

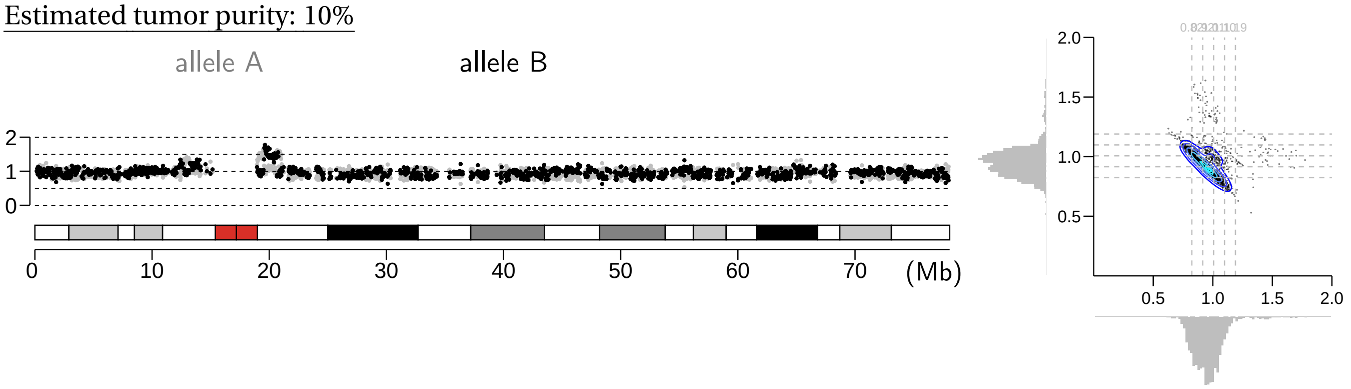

Estimated tumor purity: 18%

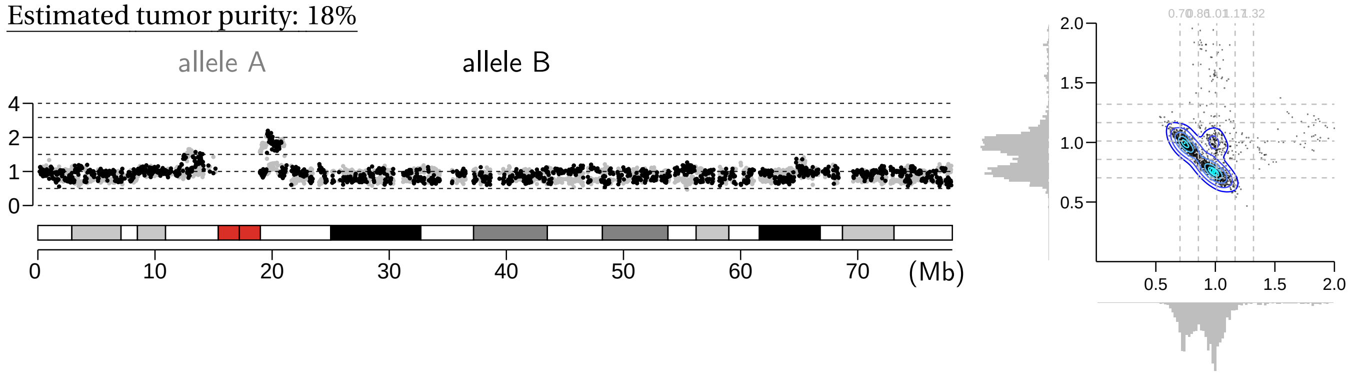

Estimated tumor purity: 16%

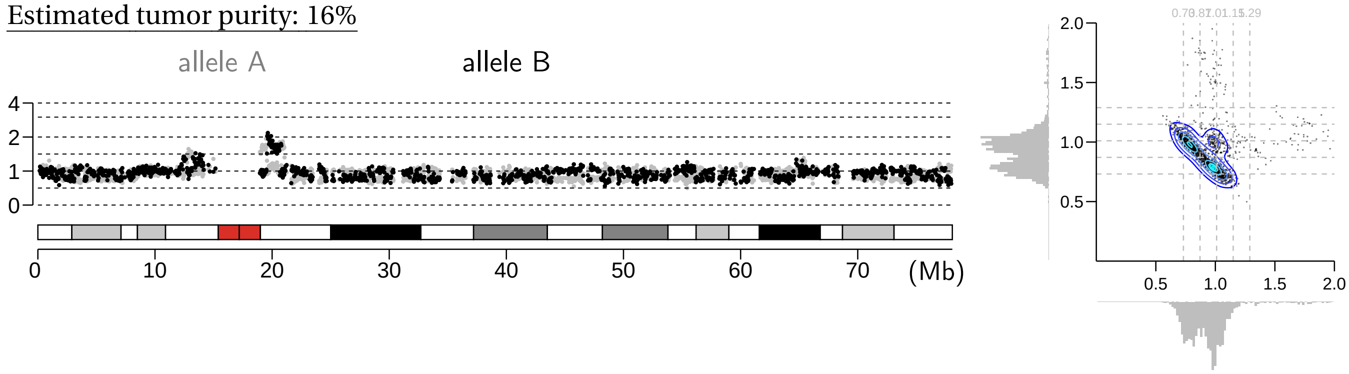

Estimated tumor purity: 14%

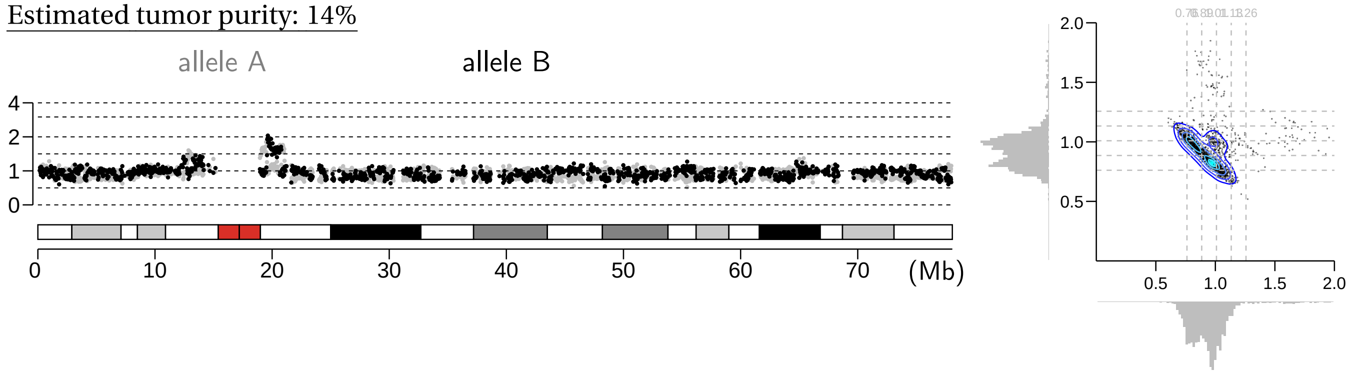

Estimated tumor purity: 12%

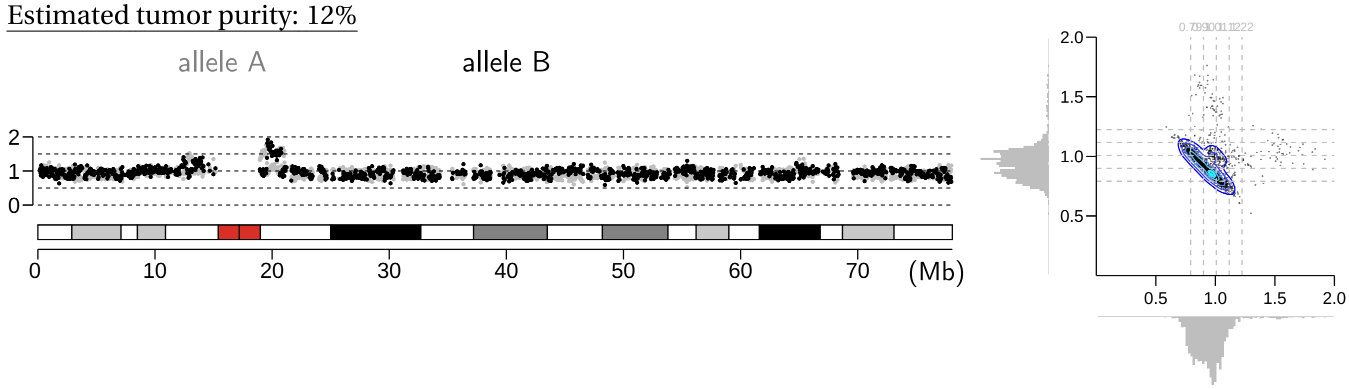

Supplement: Supplementary file 4 — Supplementary Data 1-10 [file 41467_2023_41805_MOESM4_ESM.zip › Supplementary.Data/Supplementary.Data.9.DownSamplePlots.pdf]
