# Supplementary material for: Genomic signatures of past and present chromosomal instability in Barrett’s esophagus and early esophageal adenocarcinoma
Source: Nat Commun. 2023 Oct 4;14:6203. doi: 10.1038/s41467-023-41805-6 (PMC10550953; doi:10.1038/s41467-023-41805-6)
Supplement: Supplementary file 5 — Reporting Summary [file 41467_2023_41805_MOESM5_ESM.pdf]

nature portfolio

Corresponding author(s): Cheng-Zhong Zhang, Matthew Stachler, and Adam Bass

Last updated by author(s): 8/17/2023

Reporting Summary

Nature Portfolio wishes to improve the reproducibility of the work that we publish. This form provides structure for consistency and transparency in reporting. For further information on Nature Portfolio policies, see our Editorial Policies and the Editorial Policy Checklist.

Statistics

For all statistical analyses, confirm that the following items are present in the figure legend, table legend, main text, or Methods section.

n/a	Confirmed	
☐	☒	The exact sample size (*n*) for each experimental group/condition, given as a discrete number and unit of measurement
☐	☒	A statement on whether measurements were taken from distinct samples or whether the same sample was measured repeatedly
☐	☒	The statistical test(s) used AND whether they are one- or two-sided<br>*Only common tests should be described solely by name; describe more complex techniques in the Methods section.*
☒	☐	A description of all covariates tested
☐	☒	A description of any assumptions or corrections, such as tests of normality and adjustment for multiple comparisons
☐	☒	A full description of the statistical parameters including central tendency (e.g. means) or other basic estimates (e.g. regression coefficient) AND variation (e.g. standard deviation) or associated estimates of uncertainty (e.g. confidence intervals)
☐	☒	For null hypothesis testing, the test statistic (e.g. *F*, *t*, *r*) with confidence intervals, effect sizes, degrees of freedom and *P* value noted<br>*Give P values as exact values whenever suitable.*
☒	☐	For Bayesian analysis, information on the choice of priors and Markov chain Monte Carlo settings
☒	☐	For hierarchical and complex designs, identification of the appropriate level for tests and full reporting of outcomes
☒	☐	Estimates of effect sizes (e.g. Cohen's *d*, Pearson's *r*), indicating how they were calculated

Our web collection on statistics for biologists contains articles on many of the points above.

Software and code

Policy information about availability of computer code

Data collection	No software or code was used for data collection.
Data analysis	Computational algorithms are described in detail in the Method section; implementations of the computational algorithms using scripting languages, including R and Matlab, are deposited into a Zenodo repository that also contains compiled results from each stage of the calculation. Usage of published or public bioinformatic packages is stated in Methods with references to either the publications or the repositories of the software packages. Sequencing reads were aligned to the NCBI Human Reference Genome Build GRCh37/hg19 using bwa (version 0.7.7). Standard aligning and processing used the standard pipeline established by the Genomics Platform at the Broad Institute, as described in the GATK best practice (https://gatk.broadinstitute.org/hc/en-us/articles/360035535912) utilizing GATK Mutect2 (version 4.0.1.2), Oncotator (version 1.9.9.0), HaplotypeCaller (GATK HaplotypeCaller v.4.0.12.0-6), GATK4 Somatic CNV ModelSegments pipeline (version 4.0.1.2), ABSOLUTE (version 1.5), Sanger Imputation Server for statistical phasing using EAGLE2 (version 2.0.5), and SvABA (version 1.1.3) as described and referenced in the manuscript. Single cell analysis utilized the above with addition of ASEReadCounter module from GATK4 (version 4.0.1.2).<br><br>All the algorithms and bioinformatic pipelines implemented in this study are described in Methods; scripts and codes are available from https://github.com/chunyangbao/NC_ESAD75

For manuscripts utilizing custom algorithms or software that are central to the research but not yet described in published literature, software must be made available to editors and reviewers. We strongly encourage code deposition in a community repository (e.g. GitHub). See the Nature Portfolio guidelines for submitting code & software for further information.

Data

Policy information about availability of data

All manuscripts must include a data availability statement. This statement should provide the following information, where applicable:

- Accession codes, unique identifiers, or web links for publicly available datasets
- A description of any restrictions on data availability
- For clinical datasets or third party data, please ensure that the statement adheres to our policy

Raw whole-genome sequencing data generated in the current study have been deposited into the database of Genotypes and Phenotypes (dbGaP) with accession code phs002706 [http://www.ncbi.nlm.nih.gov/projects/gap/cgi-bin/study.cgi?study_id=phs002706.v1.p1]. Sequencing data are released with controlled access according to the approved IRB Protocols and the study protocol of the sequencing experiment. Data management, including approval for data access and reuse, and duration of data availability, is managed by dbGaP. Longitudinal BE sequencing data were obtained from the European Genome-phenome Archive (EGA) under accession code EGAD00001006033 [https://ega-archive.org/datasets/EGAD00001006033] through a data access agreement approved by the International Cancer Genome Consortium. The following data/results have been also uploaded to Zenodo [https://zenodo.org/record/8265676] and are publicly available: For sequencing data of BE/EAC samples in the current cohort: (1) intermediate and final haplotype-specific DNA copy number data and plots (grouped by patient and shown for each chromosome); (2) structural rearrangements; (3) somatic short sequence variants (single-nucleotide substitutions and insertion/deletions); (4) DNA copy-number of single cells from a HGD lesion. For the longitudinal sequencing data, we only provided unphased DNA copy number plots of each chromosome in each sample from each patient.

Human research participants

Policy information about studies involving human research participants and Sex and Gender in Research.

Reporting on sex and gender	Sex and gender information was not reported directly though the biological sex can be inferred from the DNA copy-number data of sex chromosomes. We did not explicitly consider sex as a variable in the analysis as the sample size of the current study (15 individuals) is too small to assess sex-related differences. Additionally, the disease under study (esophageal adenocarcinoma) displays a significant bias of male incidence (~4:1) that limits the number of female samples available. All samples meeting histologic criteria were selected regardless of patient's sex. The representation of biological male and female patients in our cohort roughly matches the male:female incidence ratio from large-scale epidemiological analyses.
Population characteristics	Ethnic/age/ancestral information was not considered or reported in sample identification. All samples were collected from patients without prior treatment who had archived pathology samples within the UPMC or BWH hospital system. All ethnic, age (18+), and ancestral backgrounds were eligible.
Recruitment	The sample identification and collection procedure is described in detail in the Sample Identification subsection of Methods. Ethnic/ancestral information was not collected or considered during sample collection due to the rarity of samples and due to the disease being much more common in Caucasian males. All patients over 18 years of age were eligible.
Ethics oversight	All samples were collected following IRB approval from individual institutions (Brigham and Women's Hospital, University of Pittsburgh School of Medicine, and Mayo Clinic) listed in the Sample Identification section of Methods. The study was performed under IRB approval from Brigham and Women's Hospital.

Note that full information on the approval of the study protocol must also be provided in the manuscript.

Field-specific reporting

Please select the one below that is the best fit for your research. If you are not sure, read the appropriate sections before making your selection.

☒ Life sciences ☐ Behavioural & social sciences ☐ Ecological, evolutionary & environmental sciences

For a reference copy of the document with all sections, see nature.com/documents/nr-reporting-summary-flat.pdf

Life sciences study design

All studies must disclose on these points even when the disclosure is negative.

Sample size	The samples were identified from an exhaustive pathological re-analyses of endoscopic mucosal resection slides from > 500 patients. Due to the rarity of samples, we had chosen to analyze all identified samples to maximize the statistical power of the analysis.
Data exclusions	Only samples whose DNA libraries had low library complexity (from which one cannot generate sufficient sequencing data) were excluded. This was clearly stated in Lines 72-73 of the main text and in the Method.
Replication	The findings from the analyses of the primary data cohort (multi-focal BE/EAC samples) were replicated once with additional analyses of longitudinal BE sequencing data from a prior study.
Randomization	As no interventions or different groups were in the study, no randomization was performed.

Blinding	As the study was designed to only analyze patients with early esophageal adenocarcinoma and did not look at ourcome data, no blinding was performed.

Reporting for specific materials, systems and methods

We require information from authors about some types of materials, experimental systems and methods used in many studies. Here, indicate whether each material, system or method listed is relevant to your study. If you are not sure if a list item applies to your research, read the appropriate section before selecting a response.

Materials & experimental systems

n/a	Involved in the study
☒	☐ Antibodies
☒	☐ Eukaryotic cell lines
☒	☐ Palaeontology and archaeology
☒	☐ Animals and other organisms
☒	☐ Clinical data
☒	☐ Dual use research of concern

Methods

n/a	Involved in the study
☒	☐ ChIP-seq
☒	☐ Flow cytometry
☒	☐ MRI-based neuroimaging
